# Nickel-mediated N–N bond formation and N_2_O liberation *via* nitrogen oxyanion reduction†

**DOI:** 10.1039/d1sc02846d

**Published:** 2021-07-13

**Authors:** Daniel M. Beagan, Alyssa C. Cabelof, Maren Pink, Veronica Carta, Xinfeng Gao, Kenneth G. Caulton

**Affiliations:** Indiana University, Department of Chemistry 800 E. Kirkwood Ave. Bloomington IN 47401 USA caulton@indiana.edu

## Abstract

The syntheses of (DIM)Ni(NO_3_)_2_ and (DIM)Ni(NO_2_)_2_, where DIM is a 1,4-diazadiene bidentate donor, are reported to enable testing of bis boryl reduced N-heterocycles for their ability to carry out stepwise deoxygenation of coordinated nitrate and nitrite, forming O(Bpin)_2_. Single deoxygenation of (DIM)Ni(NO_2_)_2_ yields the tetrahedral complex (DIM)Ni(NO)(ONO), with a linear nitrosyl and κ^1^-ONO. Further deoxygenation of (DIM)Ni(NO)(ONO) results in the formation of dimeric [(DIM)Ni(NO)]_2_, where the dimer is linked through a Ni–Ni bond. The lost reduced nitrogen byproduct is shown to be N_2_O, indicating N–N bond formation in the course of the reaction. Isotopic labelling studies establish that the N–N bond of N_2_O is formed in a bimetallic Ni_2_ intermediate and that the two nitrogen atoms of (DIM)Ni(NO)(ONO) become symmetry equivalent prior to N–N bond formation. The [(DIM)Ni(NO)]_2_ dimer is susceptible to oxidation by AgX (X = NO_3_^−^, NO_2_^−^, and OTf^−^) as well as nitric oxide, the latter of which undergoes nitric oxide disproportionation to yield N_2_O and (DIM)Ni(NO)(ONO). We show that the first step in the deoxygenation of (DIM)Ni(NO)(ONO) to liberate N_2_O is outer sphere electron transfer, providing insight into the organic reductants employed for deoxygenation. Lastly, we show that at elevated temperatures, deoxygenation is accompanied by loss of DIM to form either pyrazine or bipyridine bridged polymers, with retention of a BpinO^−^ bridging ligand.

## Abstract 2

Deoxygenation of nitrogen oxyanions coordinated to nickel using reduced borylated heterocycles leads to N–N bond formation and N_2_O liberation. The nickel dimer product facilitates NO disproportionation, leading to a synthetic cycle.

## Graphical abstract

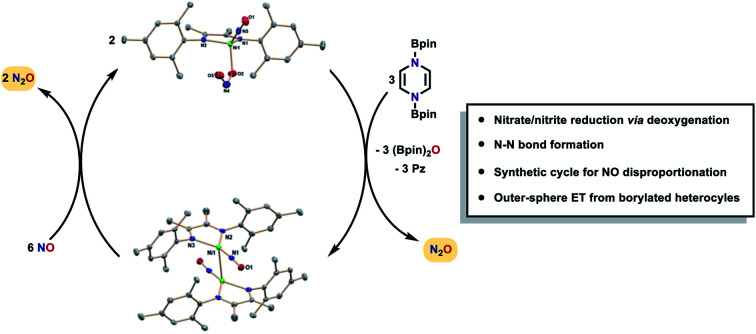

## Introduction

Reduction of CO_2_ is widely sought and has classically focused on deoxygenation to C_1_ products,^1–3^ with recent advances moving towards multi-carbon and value-added products which preserve terrestrial use of that carbon resource.^4–9^ There is a nitrogen analogue of this challenge, accomplished biologically through denitrification, which requires an N–N bond formation upon reduction of NO_*x*_^−^ back to dinitrogen. It is widely recognized that the natural nitrogen cycle has been perturbed as an unintended consequence of inexpensive Haber–Bosch ammonia used to enhance food production.^10–12^ Soluble nitrate is abundant in runoff from agricultural fields, and accumulates in poorly flushed rivers, bays and seacoasts which ultimately leads to eutrophication and oxygen-poor “dead zones.”^13–15^

The reduction of nitrogen oxidation states is biologically accomplished by a variety of enzymes^16–23^ and its synthetic conversion is essential to repurpose environmental pollutants into value-added compounds that might be derived from nitrogen oxyanions. An attractive route to nitrogen oxyanion reduction is through deoxygenation,^24–32^ and the oxygen can be captured by protons, metal electrophiles, or main group elements known for their oxophilicity. The same is true for CO_2_ reduction and both carbon and nitrogen reduction would be richer if they could form products with C–C and N–N bonds, respectively. Designing methods to encourage such element/element reductive coupling is a valuable pursuit.

**Scheme 1:**
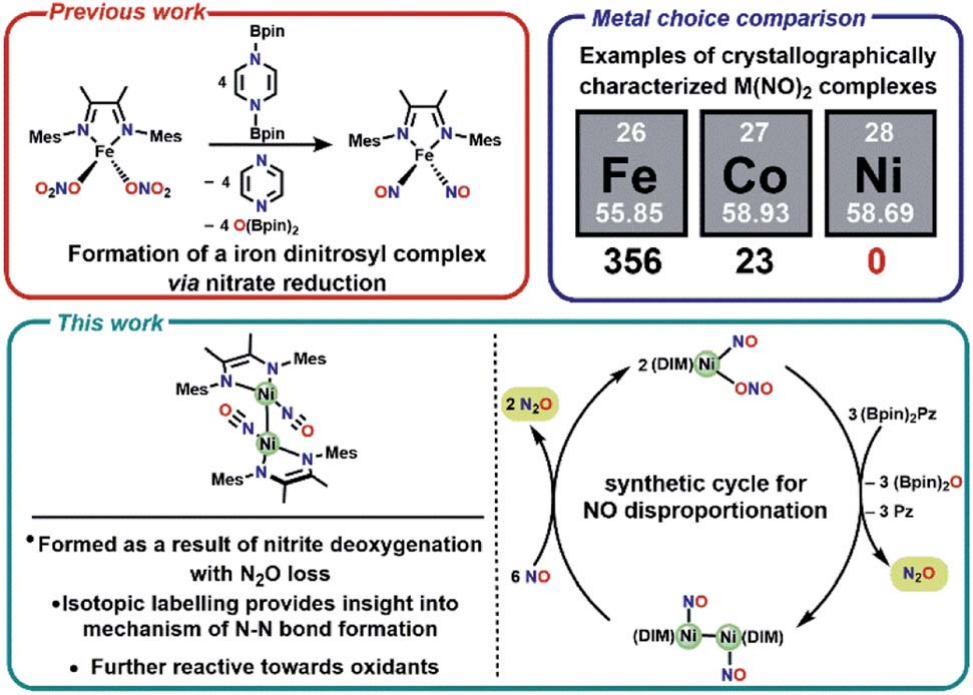
Previously reported nitrate reduction and motivation for utilizing Ni compounds (top), and summary of new reactivity presented (bottom).

One attractive strategy to promote N–N bond formation is to design complexes which pre-organize two nitrogen oxyanions at a single metal center and explore the deoxygenation chemistry of these systems. There are several reported examples of N–N bond formation at a single metal complex,^33–36^ and most terminal *cis*-hyponitrite complexes are reported with group 10 metals.^37–41^ The reagent bis-pinacolylboryl pyrazine (Scheme 1) has been shown to deoxygenate two nitrates coordinated to iron to yield a dinitrosyl iron complex.^31^ This reagent is electron rich (8π electron ring) and carries two electrophilic boron atoms making it a potent reductant with attractive kinetic reactivity, including polar B–N bonds.

We chose a bidentate ancillary ligand with a steric profile designed to allow for multidentate binding of nitrogen oxyanions if needed. These 1,4-diazadienes (DIM, Scheme 1) ligands are established to be redox active and can assist in the stabilization of low valent nickel complexes.^42–45^ Herein we show stepwise deoxygenation of oxyanions coordinated to nickel, where the location of nickel late in the 3d series disfavors two coordinated nitrosyls in comparison to Fe or Co (Scheme 1), and instead deoxygenation leads to N–N bond formation and nitrous oxide liberation.

## Results and discussion

### Nickel nitrate and nitrite complexes

**Scheme 2:**
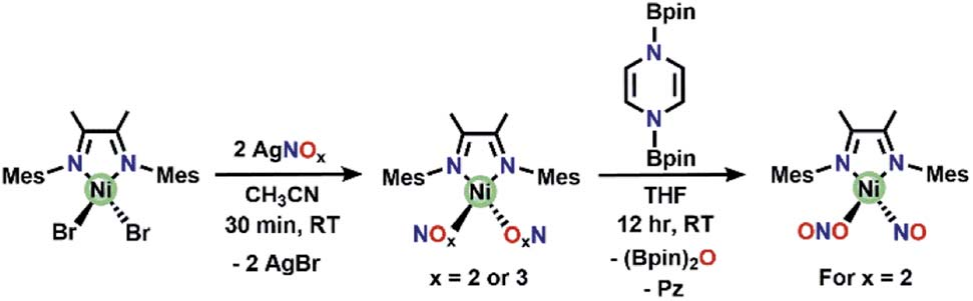
Synthesis of (DIM)Ni(NO_*x*_)_2_ complexes, as well as the synthesis of (DIM)Ni(NO)(ONO) from (DIM)Ni(NO_2_)_2_. The bis-nitrate and bis-nitrite complexes are O-bound and bidentate in the solid-state molecular structures (Fig. 1), we have drawn them as monodentate here for simplicity.

**Fig. 1:**
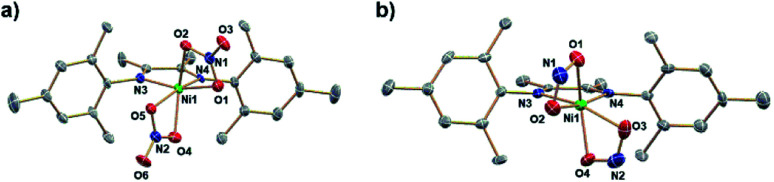
(a) ORTEP representation of the molecular structure (50% probabilities) of the nonhydrogen atoms of (a) (DIM)Ni(NO_2_)_3_ and (b) (DIM)Ni(NO_2_)_2_ (right), showing selected atom labelling (unlabelled atoms are carbon). Selected structural parameters for (DIM)Ni(NO_2_)_3_ (Å): Ni1–O1, 2.110(4); Ni1–O2, 2.093(4); Ni1–O4, 2.050(4); Ni1–O5, 2.097(4); Ni1–N3, 2.009(5); Ni1–N4, 2.058(4); O1–N1, 1.265(6); O2–N1, 1.273(6); O3–N1, 1.230(6); O4–N2, 1.298(6); O5–N2, 1.271(6); O6–N2, 1.255(6); and (DIM)Ni(NO_2_)_2_ (Å): Ni1–O1, 2.094(6); Ni1–O2, 2.091(6); Ni1–O3, 2.099(6); Ni1–O4, 2.072(6); Ni1–N3, 2.035(7); Ni1–N4, 2.038(7); O1–N1, 1.287(9); O2–N1, 1.269(9); N2–O3, 1.257(10); N2–O4, 1.290(11).

A precursor complex, (DIM)NiBr_2_, is synthesized from (DME)NiBr_2_ (DME = dimethoxyethane) and DIM in a 1 : 1 mole ratio in DCM and workup affords a red/brown solid. ^1^H NMR spectroscopy (Fig. S1†) of (DIM)NiBr_2_ reveals a paramagnetic species with resonances ranging from −22 to 36 ppm and is fully consistent with a *C*_2v_ symmetric product. Salt metathesis reactions of (DIM)NiBr_2_ with AgNO_3_ or AgNO_2_ in acetonitrile at room temperature quickly form the bis-nitrate and bis-nitrite complexes respectively with concurrent precipitation of AgBr (Scheme 2). The bis-nitrate and bis-nitrite complexes are also paramagnetic and twofold symmetric as judged by NMR spectroscopy (Fig. S3 and S5†) and their molecular structures (Fig. 1) exhibit bidentate binding of both nitrate and nitrite creating an octahedral coordination environment about the nickel metal centers.

### Single deoxygenation of (DIM)Ni(NO_2_)_2_

**Fig. 2:**
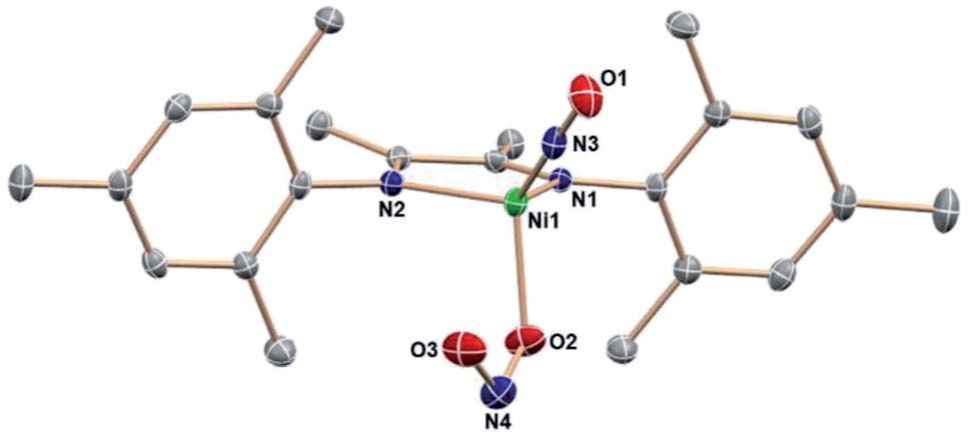
ORTEP representation of the molecular structure (50% probabilities) of the nonhydrogen atoms of (DIM)Ni(NO)(ONO) showing selected atom labelling (unlabelled atoms are carbon). Selected structural parameters: Ni1–N3, 1.637(2); Ni1–O2, 1.9952(19); Ni1–N2, 2.0075(19); Ni1–N1, 2.0204(19); O1–N3, 1.176(3), O2–N4, 1.280(3); O3–N4, 1.228(3); N4–O2–Ni1, 118.25(16); O1–N3–Ni1, 175.5(2).

Reaction of (DIM)Ni(NO_2_)_2_ with equimolar (Bpin)_2_Pz occurs to completion within 12 h at room temperature in THF with a color change from brown to green (Scheme 2). Analysis by ^1^H NMR spectroscopy shows complete consumption of the boryl reagent, formation of equimolar pyrazine and (Bpin)_2_O and a diamagnetic metal complex with the expected 4 : 6 : 6 : 12 intensity ^1^H NMR resonances of a single DIM-containing product. Workup by pentane wash to remove organic materials yields a green solid, and the molecular structure (Fig. 2) confirms the presence of the singly deoxygenated species (DIM)Ni(NO)(ONO), assigned as {Ni(NO)}^10^ in the Enemark–Feltham notation. The Ni1–N3–O1 angle is linear (175.5°), and the complex has a strong nitrosyl stretching frequency at 1785 cm^−1^, similar to other LNi(NO)(X) complexes.^46–49^ The nitrito linkage in (DIM)Ni(NO)(ONO) contrasts the N-bound nitrite linkage in (Me_2_-*ortho*-phenanthroline)Ni(NO)(NO_2_).^49^

### Deoxygenation of (DIM)Ni(NO)(ONO)

**Fig. 3:**
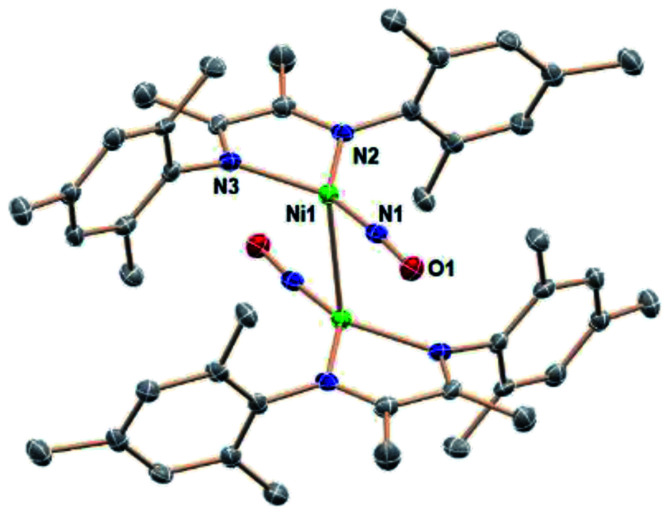
ORTEP representation of the molecular structure (50% probabilities) of the nonhydrogen atoms of centrosymmetric [(DIM)Ni(NO)]_2_, showing selected atom labelling (unlabelled atoms are carbon). Selected structural parameters: Ni1–N3, 1.960(4) Å; Ni1–N2, 1.893(4); Ni1–N1, 1.647(3); O1–N1, 1.193(4); O1–N1–Ni1 172.5(3)°.

The tetrahedral nickel(0) (DIM)Ni(NO)(ONO) complex reacts with (Bpin)_2_Pz (1 : 1.5 mole ratio) at room temperature in THF. Monitoring the reaction by ^1^H NMR spectroscopy shows the formation of boryl ether and pyrazine, as well as the depletion of signals for (DIM)Ni(NO)(ONO). Crystalline precipitate forms during the course of the reaction. Executing this reaction in CD_3_CN shows complete consumption of starting materials in less than four hours at room temperature. All DIM-containing product is insoluble based on the lack of any ligand resonances, but the pyrazine and boryl ether byproducts are detected by ^1^H NMR spectroscopy (Fig. S19†) in the correct stoichiometric ratio in the colorless solution. This confirms that no free ligand is formed upon deoxygenation, nor any degradation products containing the DIM ligand. The reaction executed in CD_2_Cl_2_ shows the same result, including no detectable ^1^H NMR signals of the product nickel complex. This solvent gives slightly larger crystals, which were used for the better of two single crystal structure determinations. The molecular structure (Fig. 3) as determined by SCXRD reveals the product to be [(DIM)Ni(NO)]_2_, a dimer with a Ni–Ni bond.

[(DIM)Ni(NO)]_2_ contains a crystallographic inversion center at the Ni–Ni midpoint and the Ni–Ni bond length (2.6103(10) Å) is short compared to other Ni–Ni bonds with no bridging ligands.^50–53^ There are no other examples of two Ni–NO units linked solely by a Ni–Ni bond. The Ni lies only 0.16 Å out of the three-nitrogen atom plane, and the Ni′/N1 distance is longer by 1.24 Å, indicating that the NO is not bridging. Analysis of the bond lengths in the DIM backbone (N–C–C–N) (Table S1†) reveals a long-short-long pattern (*vs.* the comparison compounds in Table S1†) consistent with increased electron density in the di-imine moiety. The C–C bond in particular is quite short (1.436 Å) compared to the other (DIM)Ni complexes, indicative of either a singly or doubly reduced ligand. The monomer (DIM)Ni(NO) is an odd-electron (radical) species which dimerizes to the crystallographically observed product. The Δ*G*° for dissociation of the dimer into two (DIM)Ni(NO) radical monomers is calculated to be 14.7 kcal mol^−1^, similar to reported values for dissociation of an iron carbonyl dimer.^54^ The MALDI-TOF spectrum (Fig. S18†) of [(DIM)Ni(NO)]_2_ has an *m*/*z* at 816.3, consistent with the intact dimer. The trans rotational conformation around the nickel–nickel bond is sterically favored. This conformation has the stabilizing effect of opposing the dipoles in each (DIM)Ni(NO) monomer and benefits from opposed NO^*δ*+^ to DIM^*δ*−^ attraction between monomer units. Because the dimeric product is insoluble in a wide range of solvents of polarity up to acetonitrile and dichloromethane, a ^1^H NMR spectrum could not be obtained. The solid state IR spectrum (Fig. S15†) shows a strong NO absorption at 1664 cm^−1^, a low value consistent with greater back donation to the nitrosyl than in (DIM)Ni(NO)(ONO). This can be attributed to the increased donor ability of the reduced DIM ligand as well as better orbital overlap.

**Scheme 3:**
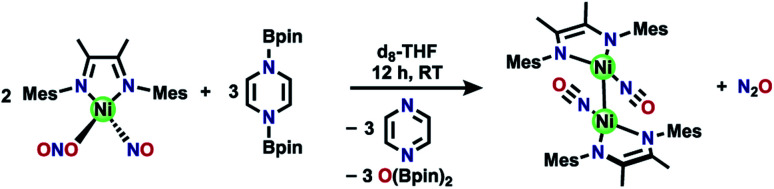
Synthesis of [(DIM)Ni(NO)]_2_ from (DIM)Ni(NO)(ONO) and (Bpin)_2_Pz.

The reaction of (DIM)Ni(NO)(ONO) with equimolar (Bpin)_2_Pz shows complete consumption of borylated reagent along with incomplete consumption of nickel starting material. In order to establish the correct stoichiometry, (DIM)Ni(NO)(ONO) was reacted with excess (3 : 1 mole ratio) (Bpin)_2_Pz in the presence of naphthalene as an internal standard. From this experiment, pyrazine is produced in a 1 : 1.5 mole ratio with respect to the (DIM)Ni(NO)(ONO) starting material, indicating a 2 : 3 stoichiometry of nickel to borylating reagent. Labelling studies are employed here for characterization, and later for mechanistic insight. The nitrosyl stretch shifts to 1625 cm^−1^ (Fig. S16†) for isotopically labelled [(DIM)Ni(^15^NO)]_2_, which is consistent with the expected reduced mass calculation. The reduced nitrogen containing byproduct upon deoxygenation of (DIM)Ni(NO)(ONO) was confirmed to be N_2_O *via* vacuum transfer of the headspace volatiles to a gas IR cell, which clearly show the P and R bands of N_2_O at 2224 cm^−1^ (Fig. S30†). All of this allows us to write a balanced reaction for the formation of [(DIM)Ni(NO)]_2_ with concomitant loss of N_2_O (Scheme 3).

Deoxygenation of isotopically labelled (DIM)Ni(^15^NO)(O^15^NO) results in the formation of (^15^N)_2_O, evidenced by a band at 2158 cm^−1^*via* gas IR (Fig. S30†). The deoxygenating agent employed in Scheme 3 can be replaced by the reduced 4,4′-bipyridine analogue, (Bpin)_2_Bpy, which reacts completely within 2 hours. The faster deoxygenation using (Bpin)_2_Bpy *vs.* (Bpin)_2_Pz is a consequence of reduced bipyridine being a more potent reductant than reduced pyrazine.^55^

### XPS of nickel complexes

XPS provides useful resolution of the chemical environment in these four molecules (see Fig. S44†). The nitrogen 1s region resolves nitrate, nitrite, nitrosyl and all the DIM nitrogen atoms, with progressively lower NO_*x*_ binding energy along that series. The oxygen 1s intensity decreases progressively from nitrate to nitrite to nitrosyl when integrated with respect to the nitrogen. Compound (DIM)Ni(NO)(ONO) shows two oxygen 1s binding energies with relative intensities of 1 : 2, indicating resolution of two of the three types of oxygens. The oxygen 1s binding energy decreases progressively from (DIM)Ni(NO_3_)_2_ to (DIM)Ni(NO_2_)_2_ to [(DIM)Ni(NO)]_2_, consistent with a lower nitrogen oxidation state being less electron withdrawing towards the oxygen. Although linear nitrosyl and nitrite are both formally trivalent nitrogen, the lower binding energy of oxygen by 0.57 eV in [(DIM)Ni(NO)]_2_ compared to (DIM)Ni(NO_2_)_2_ can be attributed to the increased backdonation into the nitrosyl of the dimer, consistent with the observed low *ν*_NO_ stretching frequency. The nitrogen 1s spectrum of [(DIM)Ni(NO)]_2_ resolves the nitrosyl from imine in the intensity ratio of 1 : 2. The Ni 2p spectrum shows a lower metal binding energy for [(DIM)Ni(NO)]_2_ compared to the bis-nitrate and bis-nitrite complexes, consistent with it having a lower metal oxidation state.

### Redox reactivity of [(DIM)Ni(NO)]_2_

**Scheme 4:**
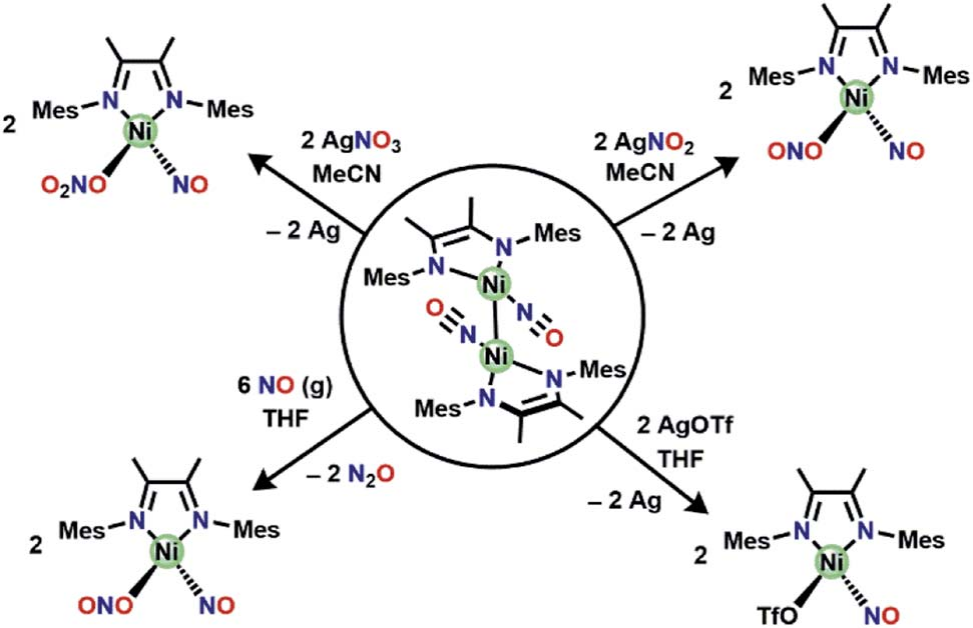
Summary of oxidative reactivity with [(DIM)Ni(NO)]_2_.

Despite the insolubility of [(DIM)Ni(NO)]_2_, it reacts readily (Scheme 4) with oxidants to cleave the Ni–Ni bond. The reaction between a slurry of [(DIM)Ni(NO)]_2_ with AgNO_2_ or AgNO_3_ results in homogeneity of the solution and a color change to green in both cases. Isolation of the green solid from the reaction with AgNO_2_ reveals the product to be the previously characterized (DIM)Ni(NO)(ONO) by ^1^H and ^13^C NMR spectroscopy along with the diagnostic NO stretch at 1785 cm^−1^. The corresponding reaction of the dimer and AgNO_3_ results in the formation of the {Ni(NO)}^10^ complex (DIM)Ni(NO)(ONO_2_), indicating that the one electron oxidation product of [(DIM)Ni(NO)]_2_ is susceptible to nucleophilic attack even by weak nucleophiles such as nitrate. It is important here to control the Ag : Ni stoichiometry because both (DIM)Ni(NO)(ONO) and (DIM)Ni(NO)(ONO_2_) are subject to further oxidation by silver salts, forming the bis-nitrite or bis-nitrate complexes respectively. The oxidations of the mixed oxyanion complexes result in liberation of an equivalent of nitric oxide, which was detected *via* gas phase IR (Fig. S34†). There is precedence for nitric oxide liberation upon one electron oxidation of [Ni(NO)(bipy)][PF_6_] with either AgPF_6_ or [NO][PF_6_] as the oxidant.^48^

**Fig. 4:**
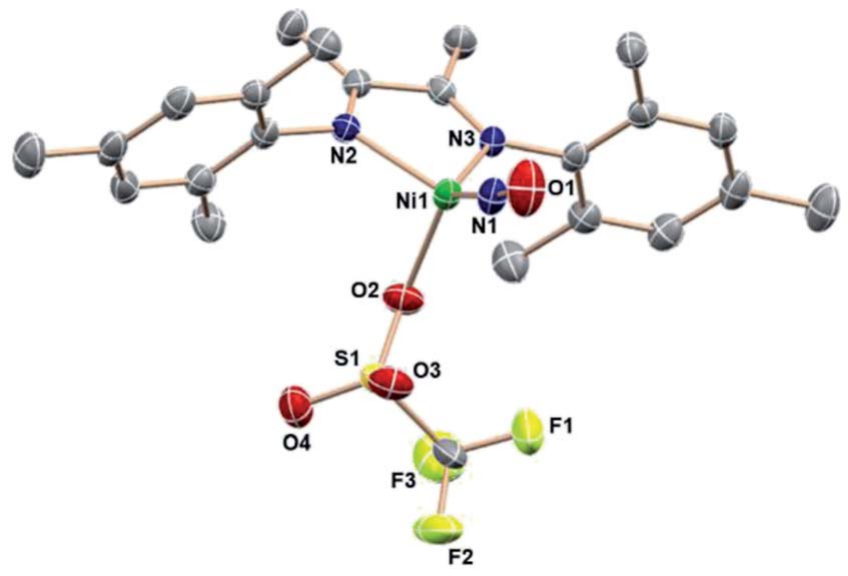
ORTEP representation of the molecular structure (50% probabilities) of the nonhydrogen atoms of (DIM)Ni(NO)(OTf), showing selected atom labeling (unlabelled atoms are carbon). Selected structural parameters (Å, °): Ni1–O2, 2.059(2); Ni1–N3, 1.994(2); Ni1–N2, 2.010(2); Ni1–N1, 1.630(2); O1–N1–Ni1, 173.1(3); N1–Ni1–O2, 124.22(11).

Even an oxidant with a more weakly nucleophilic triflate anion succeeds in this oxidative process. The reaction of [(DIM)Ni(NO)]_2_ with AgOTf in THF results in a color change to green and homogeneity of the solution. The ^19^F NMR spectrum (Fig. S21†) is indicative of one diamagnetic product, and the ^1^H NMR (Fig. S21†) spectrum indicates twofold symmetry. The solid state nitrosyl stretch appears at 1780 cm^−1^ (Fig. S22†) which is shifted to lower energy by 100 cm^−1^ compared to authentic 3-coordinate (bpy)Ni(NO)^+^ (bpy = bipyridine).^49^ The shift in *ν*_NO_ can be attributed to coordination of THF solvent to give the 4-coordinate species [(DIM)Ni(NO)(THF)]^+^. When the reaction of [(DIM)Ni(NO)]_2_ and AgOTf is repeated in the non-coordinating solvent DCM, the *ν*_NO_ is higher by 31 cm^−1^ in the solid state (Fig. S23†) and 45 cm^−1^ in a DCM solution (Fig. S24†). If the same material is redissolved in THF, the IR spectrum shows two nitrosyl stretches suggesting an equilibrium of THF binding to the nickel center. Crystals were grown *via* vapor diffusion of pentane into a concentrated THF solution and the solid state molecular structure (Fig. 4) shows the {Ni(NO)}^10^ complex (DIM)Ni(NO)(OTf), with triflate coordinated to nickel. However, based on spectroscopic data, the OTf is in equilibrium when dissolved in coordinating solvents.

With this weak nucleophile, the tetrahedron is distorted to flatten the (DIM)NiN(O) plane: the angle from the DIM N–C=C–N midpoint to the Ni center to N_1_ is 135.6°. Regardless of the abundance of THF as crystallization solvent, this neutral molecule is apparently the least soluble equilibrium participant.

### Nitric oxide disproportionation

With the knowledge that [(DIM)Ni(NO)]_2_ reacts rapidly with silver oxidants, we were interested in its reactivity with NO gas. A suspension of [(DIM)Ni(NO)]_2_ in THF was exposed to excess NO and there was an immediate color change to a homogeneous brown/green solution. Workup of the solution indicates that the nickel-containing product of this reaction is (DIM)Ni(NO)(ONO), which is established by a variety of spectroscopic techniques along with an independent unit cell determination of the molecular structure *via* SCXRD. Vacuum transfer of the volatiles generated during the reaction into a gas IR cell show the formation of N_2_O (Fig. S31†). This reaction therefore accomplishes disproportionation of nitric oxide to N_2_O and NO_2_, but the latter undergoes one electron reduction by nickel to yield the coordinated nitrito product (Scheme 4).

**Scheme 5:**
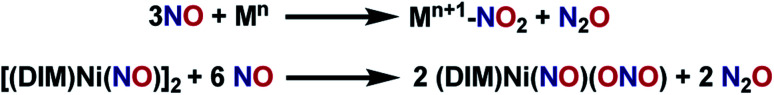
Metal-mediated nitric oxide disproportionation.

As evidenced by Scheme 5, two low valent nickel centers of [(DIM)Ni(NO)]_2_ mediate the disproportionation of 6 moles of NO resulting in the formation of two Ni-(ONO) complexes and two moles of N_2_O, a reaction which has precedence with first row transition metals.^49,56–63^ The reaction of [(DIM)Ni(NO)]_2_ with NO gas can be accomplished in the absence of solvent. Addition of excess nitric oxide to a solid sample of [(DIM)Ni(NO)]_2_ over the duration of 15 min, followed by extraction into THF indicates the formation of (DIM)Ni(NO)(ONO) *via* IR and ^1^H NMR spectroscopy. Attempts to detect an intermediate in this disproportionation reaction by solid state IR were investigated by exposing a solid sample of [(DIM)Ni(NO)]_2_ to 1–4 equivalents of nitric oxide. This resulted in up to 15% growth of (DIM)Ni(NO)(ONO) but no new intermediate terminal nitrosyl and no growth of other bands between 1000 and 1600 cm^−1^ (Fig. S37†). The fact that this reaction occurs cleanly in the solid state, where diffusive separation of intermediate nickel monomers is physically inhibited, makes it plausible that an intermediate is two identical (DIM)Ni(NO)_2_.

### Nitrogen isotope label studies

#### Preparation of isotopomers

**Fig. 5:**
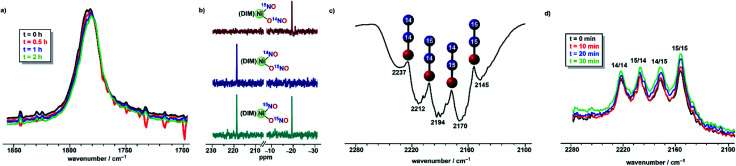
(a) Solution IR of the nitrosyl stretch in (DIM)Ni(^14^NO)(O^15^NO) monitored over time (b) ^15^N NMR of (DIM)Ni(^15^NO)(O^15^NO) (bottom), (DIM)Ni(^14^NO)(O^15^NO) (middle), and (DIM)Ni(^15^NO)(O^14^NO) (top) all in CD_2_Cl_2_ at −42 °C (c) gas phase IR spectrum of volatiles from the reaction of (DIM)Ni(^14^NO)(O^15^NO) and 3 equivalents of (Bpin)_2_Pz and (d) THF solution IR spectrum of the reaction of (DIM)Ni(^14^NO)(O^15^NO) and 3 equivalents of (Bpin)_2_Bpy.

We sought an isotopic crossover experiment with deoxygenation of (DIM)Ni(^14^NO)(O^14^NO) and (DIM)Ni(^15^NO)(O^15^NO) in a 1 : 1 mole ratio, where any detected ^14^N^15^NO or ^15^N^14^NO would indicate that the N–N bond formation occurs within a bimolecular Ni_2_ species. We isotopically labelled (DIM)Ni(^15^NO_2_)_2_ and found that it exhibits a broad ^15^N NMR chemical shift at 573.8 ppm that shows a *T*^−1^ dependence of chemical shift which is characteristic of paramagnetic complexes (Fig. S7†). Furthermore, the nitrite ligand stretches shift from 1295 cm^−1^ and 1210 cm^−1^ to 1276 cm^−1^ and 1180 cm^−1^ in the ^15^N isotopomer (Fig. S9†). Doubly labelled (DIM)Ni(^15^NO)(O^15^NO) can be synthesized by reductive borylation of the labelled (DIM)Ni(^15^NO_2_)_2_ starting material. Isotopically labelled (DIM)Ni(^15^NO)(O^15^NO) shows a diagnostic shift for both NO and nitrite IR signals (Fig. S13†). Curiously, ^15^N NMR of (DIM)Ni(^15^NO)(O^15^NO) in both THF and MeCN at room temperature shows only one signal at 3.6 ppm (Fig. S11†) despite chemically inequivalent nitrogen atoms. However, room temperature ^15^N NMR in DCM (Fig. S11†) shows the same signal at ∼3.6 ppm along with a broad signal at 206 ppm. Cooling this solution to −42 °C results in two sharp signals at −19.1 and 216.3 ppm in a 1 : 1 intensity ratio (Fig. 5). To confirm the assignment of the two signals, the isotopomers (DIM)Ni(^14^NO)(O^15^NO) and (DIM)Ni(^15^NO)(O^14^NO) were synthesized *via* the reaction of the appropriately labelled [(DIM)Ni(NO)]_2_ dimer with either AgNO_2_ or Ag^15^NO_2_ (see ESI† for isotopic details). At −42 °C in DCM, ^15^N NMR of (DIM)Ni(^14^NO)(O^15^NO) shows one sharp signal at 216.3 ppm, and (DIM)Ni(^15^NO)(O^14^NO) shows one sharp signal at −19.1 ppm (Fig. 5). The specific isotopomers confirm that the original unseen ^15^N NMR signal of (DIM)Ni(^15^NO)(O^15^NO) in THF and MeCN corresponds to the nitrito ligand, consistent with a dynamic process that exchanges the nitrito ligand between nickel complexes. This dynamic process is confirmed *via* mass spectrometry with a 1 : 1 THF solution of (DIM)Ni(^15^NO)(O^15^NO) and (DIM)Ni(^14^NO)(O^14^NO). The ESI+ spectrum is consistent with isotopic scrambling which shows a statistical mixture of the original doubly labelled and unlabelled compounds, together with equal amounts of (DIM)Ni(^14^NO)(O^15^NO) and (DIM)Ni(^15^NO)(O^14^NO) (Fig. S38†). The presence of the mixed labelled isotopomers is consistent with a dynamic process of intermolecular nitrite exchange between metal centers, and this dynamic process is supported by the line broadening ^15^N NMR observations above.

#### Insight into the N–N bond formation

The nitrito scrambling in a 1 : 1 mixture of (DIM)Ni(^15^NO)(O^15^NO) and (DIM)Ni(^14^NO)(O^14^NO) precludes any mechanistic insight obtained from a mixture of these two complexes. Instead, we focused on the individual isotopomers (DIM)Ni(^14^NO)(O^15^NO) and (DIM)Ni(^15^NO)(O^14^NO) to obtain mechanistic information about the formation of N_2_O. Both (DIM)Ni(^14^NO)(O^15^NO) and (DIM)Ni(^15^NO)(O^14^NO) are isotopically pure as observed by the nitrosyl stretching frequencies and ^15^N NMR (Fig. 5), and monitoring a solution of either isotopomer in THF shows no isotopic scrambling as judged by ^15^N NMR and IR spectroscopy (Fig. 5) among the two NO_*x*_ sites during the time scale relevant to the reactivity with (Bpin)_2_Pz. Focusing on one isotopomer, the reaction between (DIM)Ni(^14^NO)(O^15^NO) and (Bpin)_2_Pz in a 2 : 3 mole ratio provides insight into the mechanism based on the nature of the N_2_O produced. Vacuum transfer of the product volatiles to a gas IR cell reveals the presence of all four isotopomers, (^15^N)_2_O, (^14^N)_2_O, ^14^N^15^NO and ^15^N^14^NO (Fig. 5), indicating the N–N bond forming event occurs in a dinickel species. Moreover, the detection of both ^14^N^15^NO and ^15^N^14^NO demands an intermediate where both nitrogen atoms are chemically equivalent, which makes (DIM)Ni(NO)_2_ a plausible precursor.

Isotopomer (DIM)Ni(^14^NO)(O^15^NO) was then reacted with (Bpin)_2_Bpy which begins to react in the time of mixing. The solution infrared spectrum (Fig. 5) recorded within five minutes of combining the reagents showed all four isotopomers of nitrous oxide. This excludes any mechanism where nitrous oxide comes exclusively from (DIM)Ni(NO)(ONO) nitrite or exclusively from its nitrosyl nitrogen. The presence of ^15^N in both Nα and Nβ of nitrous oxide requires a transition state or intermediate where the two nitrogen atoms are symmetry-equivalent and thus equally likely to become Nα or Nβ in a subsequent N–N bond forming step. In addition, seeing both unlabelled and doubly ^15^N labelled N_2_O in this experiment indicates that the N–N bond forming event occurs after formation of a dinickel intermediate because N–N bond formation at a single nickel center would give only singly labelled N_2_O. Confirming the above, the solid state IR spectrum of [(DIM)Ni(NO)]_2_ formed from (DIM)Ni(^14^NO)(O^15^NO) and 3 equivalents of (Bpin)_2_Pz shows a diagnostic nitrosyl stretches for all three expected isotopomers, [(DIM)Ni(^14^NO)]_2_, [(DIM)Ni(^15^NO)]_2_, and (DIM)_2_Ni_2_(^14^NO)(^15^NO) (Fig. S17†), consistent with computationally predicted nitrosyl stretches for each isotopomer.

#### Evidence for outer sphere electron transfer

**Fig. 6:**
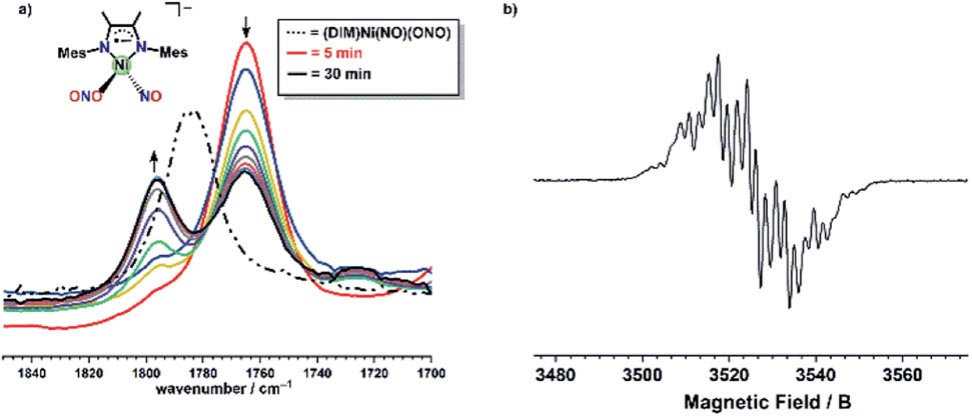
(a) Solution IR of (DIM)Ni(NO)(ONO) and (Bpin)_2_Bpy in a 1 : 1 mole ratio showing complete conversion to the reduced [(DIM)Ni(NO)(ONO)]^−^ species by the first spectrum collected (red trace), as well as the increase of the new nitrosyl stretch (solid black trace) after 30 minutes. The dotted black trace corresponds to the nitrosyl stretch of unreduced (DIM)Ni(NO)(ONO). (b) X-band EPR spectrum of [(DIM)Ni(NO)(ONO)]^−^ produced from OSET using (Bpin)_2_Bpy.

Closely monitoring the reaction by solution IR (Fig. 6) in THF shows that (DIM)Ni(NO)(ONO) is immediately converted to a new species with a *ν*_NO_ at 1765 cm^−1^, distinct from starting material (*ν*_NO_ = 1785 cm^−1^). The lower stretching frequency suggests a more electron rich complex, and the fast reaction suggests electron transfer from (Bpin)_2_Bpy to (DIM)Ni(NO)(ONO) to form the anion [(DIM)Ni(NO)(ONO)]^−^ as the identity of this first intermediate. To test this hypothesis, addition of equimolar decamethylcobaltacene (Cp^*^_2_Co) to (DIM)Ni(NO)(ONO) results in an immediate color change to dark green and solution IR after stirring for 10 minutes indicates the formation of the same [(DIM)Ni(NO)(ONO)]^−^ anion as judged by the growth of *ν*_NO_ at 1765 cm^−1^ (Fig. S40†). To further support this assignment, EPR spectroscopy of a solution of (DIM)Ni(NO)(ONO) after addition of Cp^*^_2_Co yields an EPR spectrum indicative of [(DIM)Ni(NO)(ONO)]^−^ where the electron resides in the DIM ligand backbone and couples to both nitrogen atoms and the six –CH_3_ protons (Fig. S39†). The EPR spectrum of (DIM)Ni(NO)(ONO) after addition of equimolar (Bpin)_2_Bpy (Fig. 6) is identical to the EPR obtained using Cp^*^_2_Co which confirms that outer sphere electron transfer from (Bpin)_2_Bpy to (DIM)Ni(NO)(ONO) is the first mechanistic step in reductive borylation.

The EPR spectrum obtained after a one electron reduction of (DIM)Ni(^15^NO)(O^15^NO) is identical to the EPR spectrum of doubly ^14^N complex, indicating that the nitrosyl and nitrito nitrogen atoms are not involved in the coupling observed. The DIM ligand-based reduction is corroborated by density functional theory (DFT) electronic structure analysis of [(DIM)Ni(NO)(ONO)]^−^ where the spin density and corresponding orbital diagram are consistent with a DIM ligand-based SOMO (Fig. S48†). Within 30 minutes of the reaction, there is a clear appearance of a second intermediate with *ν*_NO_ at 1795 cm^−1^ (Fig. 6). This intermediate is a result of downstream borylation, as this nitrosyl stretch at 1795 cm^−1^ does not appear following electron transfer from Cp^*^_2_Co.

#### Candidate intermediates by DFT calculation

After gaining experimental insight into the mechanism of the formation of [(DIM)Ni(NO)]_2_, we employed density functional theory at the B3LYP-D3BJ/6-311G(d,p) level of theory to evaluate candidate intermediates after single deoxygenation of (DIM)Ni(NO)(ONO), which initiates the reaction. For the intermediate with the chemical formula (DIM)Ni(N_2_O_2_), we optimized both a nickel dinitrosyl (singlet and triplet) and a nickel hyponitrite (only singlet) (Fig. S47†). The optimized *S* = 0 nickel dinitrosyl was found to be 37 kcal mol^−1^ higher in energy than the nickel hyponitrite and has one linear and one bent nitrosyl ligand. The *S* = 1 dinitrosyl was 7.3 kcal mol^−1^ lower in energy than the singlet, with two bent nitrosyl ligands (Ni–N–O = 139°) and pseudo *C*_2_ symmetry. Based on the corresponding orbitals and the spin density plot of *S* = 1 (DIM)Ni(NO)_2_, there is significant unpaired electron density on the nitrosyl ligands (Fig. S49†). Despite both dinitrosyl species being higher in energy than the calculated hyponitrite, experimental evidence suggests N–N bond formation does not happen at one nickel center. Therefore, we suspect the barrier for intramolecular hyponitrite formation from the (DIM)Ni(NO)_2_ intermediate is large. With the high energy of the dinitrosyl species, it is unlikely that (DIM)Ni(NO)_2_ would reach a concentration high enough for a bimolecular reaction between two dinitrosyl complexes, and (DIM)Ni(NO)_2_ was never observed spectroscopically. Although a transient dinitrosyl species is not a pre-requisite for N–N bond formation, one potential mechanistic pathway that coincides with experimental data is that the N–N bond is formed between transient (DIM)Ni(NO)_2_ and (DIM)Ni(NO)(ONO). Within this mechanistic pathway, we postulate that after the bimolecular collision, a series of two more deoxygenation events results in the formation of the [(DIM)Ni(NO)]_2_ dimer with liberation of N_2_O. While no intermediate bridging hyponitrite species was observed spectroscopically in the reaction, there is literature precedence for N_2_O formation from bridging hyponitrite complexes.^36,64^

#### Analogous nitrate deoxygenation

**Fig. 7:**
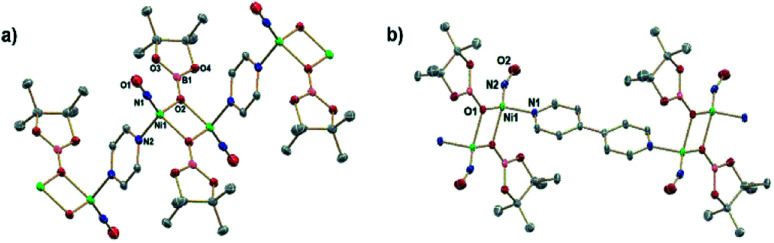
ORTEP representation of the molecular structures (50% probabilities) of the nonhydrogen atoms of (a) Ni_2_(NO)_2_(OBpin)_2_(Pz) and (b) Ni_2_(NO)_2_(OBpin)_2_(Bpy), showing selected atom labelling (unlabelled atoms are carbon). Selected structural parameters for Ni_2_(NO)_2_(OBpin)_2_(Pz) (Å): Ni1–N1, 1.634(3); Ni1–N2, 2.040(2); Ni1–O2, 1.988(2); N1–O1, 1.173(3); O1–N1–Ni1, 176.1(2); and Ni_2_(NO)_2_(OBpin)_2_(Bpy) (Å): Ni1–N1, 1.9914(17); Ni1–N2, 1.6386(19); Ni1–O1, 1.9960(16); N2–O2, 1.181(3); O2–N2–Ni1, 156.83(18).

Contrasting to the reactivity of (DIM)Ni(NO_2_)_2_, the analogous (DIM)Ni(NO_3_)_2_ is unreactive towards (Bpin)_2_Pz at room temperature. However, reaction of (DIM)Ni(NO_3_)_2_ with (Bpin)_2_Pz in d_8_-THF (mole ratio 1 : 4) at 80 °C for two hours results in a color change from brown to blue. Monitoring by ^1^H NMR spectroscopy (Fig. S28†) reveals complete consumption of (Bpin)_2_Pz and the paramagnetic (DIM)Ni(NO_3_)_2_ complex, as well as the growth of O(BPin)_2_ and pyrazine. Upon cooling the NMR sample to room temperature, X-ray quality crystals are present in the J-Young tube. Single crystal XRD of these crystals show that the molecular structure is polymeric (Fig. 7), with a repeat unit Ni_2_(NO)_2_(OBpin)_2_(Pz). Bond lengths within this structure are consistent with unreduced (neutral) pyrazine and the linear Ni–N–O is best described as tetrahedral Ni(0). Each OBpin ligand derived from deoxygenation of nitrate bridges two nickel centers creating a Ni_2_(OBpin)_2_ diamond core. We postulate that a zero valent nickel nitrosyl complex is a thermodynamic sink and having excess pyrazine in solution facilitates the crystal growth of this polymer. The OBpin ligand shows that borylation using (Bpin)_2_Pz can be stepwise and it is not limited to concerted double boryl transfer to one oxygen. The stepwise borylation is supported by the ability of the borylated N-heterocycles to engage in outer sphere electron transfer. There is precedence for OBpin as a ligand including OBpin derived from deoxygenation of CO_2_.^65–70^ The solid state IR spectrum (Fig. S29†) of Ni_2_(NO)_2_(OBpin)_2_(Pz) shows a strong NO stretch at 1810 cm^−1^. We propose that loss of DIM occurs because its bite angle (∼81°) is not ideal for the tetrahedral needs of nickel in competition with pyrazine available as another suitable donor. Free DIM is observed by ^1^H NMR spectroscopy (Fig. S28†). Reacting (DIM)Ni(NO_3_)_2_ with 4 moles of (Bpin)_2_Bpy as the deoxygenating agent at 80 °C proceeds analogously, forming Ni_2_(NO)_2_(OBpin)_2_(Bpy) (Fig. 7). The *τ*_4_ values for the nickel in the pyrazine and bipyridine linked polymers vary slightly: 0.73 and 0.67, respectively. The smaller *τ*_4_ value for the bipyridine derivative is accompanied by a smaller nitrosyl angle, with Ni1–N1–O1 being 156.84°, compared to 176.06° for the pyrazine-bridged analogue.

**Scheme 6:**
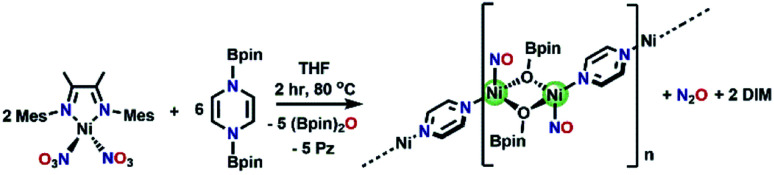
Synthesis of pyrazine linked polymer facilitated by N_2_O loss.

Much like in the synthesis of [(DIM)Ni(NO)]_2_, these polymers are deficient in nitrogen with respect to the bis-nitrate starting material. After reacting (DIM)Ni(NO_3_)_2_ with 4 moles of (Bpin)_2_Pz in THF at 80 °C for 2 hours, vacuum transfer of the headspace gas into a gas IR cell shows the characteristic doublet for N_2_O at 2224 cm^−1^ (Fig. S41†), indicating N–N bond formation. With N_2_O confirmed, as well as (Bpin)_2_O, pyrazine, and free DIM, a balanced reaction for this transformation is presented in Scheme 6. The formation of an N–N bond from the reaction of (DIM)Ni(NO)(ONO) with (Bpin)_2_Pz mirrors the N–N bond formation observed in Scheme 3; however, DIM now is replaced by bridging OBpin and bridging pyrazine or bipyridine ligands. From this result, we hypothesize that (DIM)Ni(NO)(ONO) is formed in the synthesis of polymeric Ni_2_(NO)_2_(OBpin)_2_(Pz), and DIM is lost as a result of heating after the nitrite/nitrosyl complex is formed. This is supported by the fact that (DIM)Ni(NO)(ONO) is thermally unstable, and heating in THF for 16 hours results in a color change from brown/green to blue. Monitoring this decomposition by ^1^H NMR shows the formation of many new DIM containing species, including free ligand, as judged by numerous new singlets in the aromatic region corresponding to the mesityl meta-H (Fig. S43†). Furthermore, these same polymeric structures can be obtained from the reactivity of (DIM)Ni(NO_2_)_2_ with either (Bpin)_2_Pz or (Bpin)_2_Bpy in a 1 : 2 mole ratio at 80 °C, once again showing elevated temperatures induce DIM loss and polymer formation.

## Conclusions

We have shown that reductive borylation on both (DIM)Ni(NO_2_)_2_ and (DIM)Ni(NO_3_)_2_ at elevated temperatures leads to loss of DIM and nitrous oxide formation, followed by the formation of either pyrazine or 4,4′-bipyridine bridged polymers. With milder conditions, single deoxygenation of (DIM)Ni(NO_2_)_2_ is facile to yield the mixed oxyanion compound (DIM)Ni(NO)(ONO). The (DIM)Ni(NO)(ONO) complex undergoes further deoxygenation to form the dimeric [(DIM)Ni(NO)]_2_, while also liberating nitrous oxide. Isotopic labelling studies indicate that the N–N bond formation event happens bimolecularly, with a *C*_2_ symmetric intermediate. The dimer is susceptible to oxidation with AgX (X = NO_3_^−^, NO_2_^−^, or OTf^−^) to form (DIM)Ni(NO)(X) compounds, and is competent to facilitate nitric oxide disproportionation. While mechanistically complex, we have experimental evidence that the first step in the reaction to form [(DIM)Ni(NO)]_2_ is outer sphere electron transfer from the electron rich (Bpin)_2_Bpy reductant.

## Data availability

The data supporting the findings of this study are available within the paper and its ESI files. Crystallographic data for the crystal structures has been deposited at the CCDC under 2083107 for (DIM)Ni(NO_3_)_2_, 2083108 for (DIM)Ni(NO_2_)_2_, 2083111 for (DIM)Ni(NO)(ONO), 2083110 for [(DIM)Ni(NO)]_2_, 2083109 for (DIM)Ni(NO)(OTf), 2083112 for (OBpin)_2_Ni_2_(NO)_2_(Pz), and 2083113 for (OBpin)_2_Ni_2_(NO)_2_(Bpy), and can be obtained from https://ccdc.cam.ac.uk/structures/.

## Author contributions

D. M. B. designed and performed the experiments and computations, interpreted the results and assisted with the manuscript. A. C. C. helped synthesize and characterize the nitrate/nitrite starting materials and the polymers, and assisted with the manuscript. M. P. and V. C. collected and refined the X-ray data. X. G. assisted with the nitrogen NMR experiments. K. G. C. helped design experiments, interpret results, and assisted with the manuscript writing.

## Conflicts of interest

There are no conflicts to declare.

## Supplementary Material

SC-012-D1SC02846D-s001

SC-012-D1SC02846D-s002

SC-012-D1SC02846D-s003

SC-012-D1SC02846D-s004

SC-012-D1SC02846D-s005
