# Supplementary material for: Nickel-mediated N–N bond formation and N2O liberation via nitrogen oxyanion reduction
Source: Chem Sci. 2021 Jul 13;12(31):10664–72. doi: 10.1039/d1sc02846d (PMC8356809; doi:10.1039/d1sc02846d)
Supplement: SC-012-D1SC02846D-s001 [file SC-012-D1SC02846D-s001.pdf]

Electronic Supplementary Material (ESI) for Chemical Science.
This journal is © The Royal Society of Chemistry 2021

Supplemental Information for
Nickel-mediated N-N Bond Formation and N_2O Liberation via Nitrogen Oxyanion Reduction

Daniel M. Beagan, Alyssa C. Cabelof, Maren Pink, Veronica Carta, Xinfeng Gao, Kenneth G. Caulton

Page

Experimental — **S3-S10**
- General — S4
- Synthesis of (DIM)$NiBr_2$ — S4
- Synthesis of (DIM)$Ni(NO_3)_2$ — S4
- Synthesis of (DIM)$Ni(NO_2)_2$ — S5
- Synthesis of (DIM)$Ni(NO_2)$(NO) — S6
- Synthesis of [(DIM)Ni(NO)]$_2$ — S6
- Synthesis of (DIM)Ni(NO)(OTf) — S7
- Synthesis of (DIM)Ni(NO)(ONO_2) — S7
- Synthesis of (DIM)Ni(^{15}NO)(ONO) — S8
- Synthesis of (DIM)Ni(NO)(O^{15}NO) — S8
- Synthesis of $(OBPin)_2Ni_2(NO)_2$(Pz) — S8
- Synthesis of $(OBPin)_2Ni_2(NO)_2$(Bpy) — S9

Spectral Data — **S11-S34**
- Figure S1: ^{1}H NMR of (DIM)$NiBr_2$ — S11
- Figure S2: FT-IR spectrum of (DIM)$NiBr_2$ — S11
- Figure S3a: ^{1}H NMR of (DIM)$Ni(NO_3)_2$ — S12
- Figure S3b: Variable temperature ^{1}H NMR of (DIM)$Ni(NO_3)_2$ — S12
- Figure S4: FT-IR spectrum of (DIM)$Ni(NO_3)_2$ — S13
- Figure S5a: ^{1}H NMR of (DIM)$Ni(NO_2)_2$ — S13
- Figure S5b: Variable temperature ^{1}H NMR of (DIM)$Ni(NO_2)_2$ — S14
- Figure S6: ^{15}N NMR spectrum of (DIM)Ni($^{15}NO_2$)$_2$ — S14
- Figure S7: Variable temperature ^{15}N NMR spectrum of (DIM)Ni($^{15}NO_2$)$_2$ — S15
- Figure S8: FT-IR spectrum of (DIM)$Ni(NO_2)_2$ — S15
- Figure S9: FT-IR spectrum of (DIM)$Ni(NO_2)_2$ overlay with (DIM)Ni($^{15}NO_2$)$_2$ — S16
- Figure S10: ^{1}H NMR of (DIM)Ni(NO)(ONO) — S16
- Figure S11: ^{15}N NMR spectrum of (DIM)Ni(^{15}NO)(O^{15}NO) in CD_2Cl_2 and d_8-THF — S17
- Figure S12: FT-IR spectrum of (DIM)Ni(NO)(ONO) — S17
- Figure S13: FT-IR spectrum of (DIM)Ni(NO)(ONO) overlay with (DIM)Ni(^{15}NO)(O^{15}NO) (KBr) — S18
- Figure S14: FT-IR spectrum of (DIM)Ni(NO)(ONO) overlay with (DIM)Ni(^{15}NO)(O^{15}NO) (solution) — S18
- Figure S15: FT-IR spectrum of [(DIM)Ni(NO)]$_2$ — S19
- Figure S16: FT-IR spectrum of [(DIM)Ni(^{15}NO)]$_2$ — S19
- Figure S17: FT-IR spectrum of [(DIM)Ni($^{14/15}$NO)]$_2$ — S20
- Figure S18: MALDI-TOF spectrum of [(DIM)Ni(NO)]$_2$ — S20
- Figure S19: ^{1}H NMR of (DIM)Ni(NO)(ONO) + $(Bpin)_2$Pz — S21
- Figure S20: ^{1}H NMR of (DIM)Ni(NO)(ONO) + $(Bpin)_2$Bpy — S21
- Figure S21: ^{1}H and ^{19}F NMR of (DIM)Ni(NO)(OTf) — S22
- Figure S22: FT-IR spectrum of (DIM)Ni(NO)(OTf) — S22
- Figure S23: FT-IR spectrum of (DIM)Ni(NO)(OTf) from THF vs. CH_2Cl_2 (KBr) — S23
- Figure S24: FT-IR spectrum of (DIM)Ni(NO)(OTf) from THF vs. CH_2Cl_2 (solution) — S23
- Figure S25: ^{1}H NMR of (DIM)Ni(NO)(ONO_2) — S24
- Figure S26: FT-IR spectrum of (DIM)Ni(NO)(ONO_2) — S24
- Figure S27: ^{1}H NMR of $(OBPin)_2Ni_2(NO)_2$(Pz) — S25
- Figure S28: ^{1}H NMR of the reaction to form $(OBPin)_2Ni_2(NO)_2$(Pz) — S25
- Figure S29: FT-IR spectrum of $(OBPin)_2Ni_2(NO)_2$(Pz) — S26
- Figure S30: FT-IR spectrum of N_2O formation from (DIM)Ni(NO)(ONO) + $(Bpin)_2$Pz — S26
- Figure S31: FT-IR spectrum of N_2O formation from [(DIM)Ni(NO)]$_2$ + NO — S27

Figure S32: FT-IR spectrum of the solid from $[(DIM)Ni(NO)]_2$ + NO——S27
Figure S33: ^1H NMR spectrum of the solid from $[(DIM)Ni(NO)]_2$ + NO——S28
Figure S34: FT-IR spectrum of NO from the oxidation of (DIM)Ni(NO)(ONO) with $AgNO_2$——S28
Figure S35: ^1H NMR spectrum of $(DIM)Ni(NO_2)_2$ after (DIM)Ni(NO)(ONO) + $AgNO_2$——S29
Figure S36: ^1H NMR spectrum of $(DIM)Ni(NO_3)_2$ after $[(DIM)Ni(NO)]_2$ + 2 $AgNO_3$——S29
Figure S37: FT-IR spectrum of solid state $[(DIM)Ni(NO)]_2$ + 1-4 NO——S30
Figure S38: ESI mass spectrum of (DIM)Ni(NO)(ONO) + (DIM)Ni(^{15}NO)(^{15}ONO)——S30
Figure S39: EPR spectrum of (DIM)Ni(NO)(ONO) + Cp^*_2Co——S31
Figure S40: FT-IR (solution) spectrum of (DIM)Ni(NO)(ONO) + Cp^*_2Co——S31
Figure S41: FT-IR spectrum of N_2O formation from $(DIM)Ni(NO_3)_2$ + $(Bpin)_2Pz$——S32
Figure S42: FT-IR spectrum of N_2O formation from $(DIM)Ni(NO_3)_2$ + $(Bpin)_2Bpy$——S32
Figure S43: ^1H NMR spectrum of (DIM)Ni(NO)(ONO) after thermal decomposition——S33
Figure S44: XPS spectra of various nickel complexes——S34

Crystallographic data **S35-S47**
Data for $(DIM)Ni(NO_3)_2$ ——S35-S36
General details ——S35
Data collection ——S35
Structure solution and refinement ——S36
Table S2: Crystal data and structure refinement for $(DIM)Ni(NO_3)_2$ ——S36
Data for $(DIM)Ni(NO_2)_2$ ——S36-S38
General details ——S37
Data collection ——S37
Structure solution and refinement ——S37
Table S3: Crystal data and structure refinement for $(DIM)Ni(NO_2)_2$ ——S37-S38
Data for (DIM)Ni(NO)(ONO) ——S38-S39
General details ——S38
Data collection ——S38
Structure solution and refinement ——S38
Table S4: Crystal data and structure refinement for $(DIM)Ni(NO_2)(NO)$ ——S39
Data for $[(DIM)Ni(NO)]_2$ ——S39-S41
General details ——S39
Data collection ——S40
Structure solution and refinement ——S40
Table S5: Crystal data and structure refinement for $[(DIM)Ni(NO)]_2$ ——S40
Data for (DIM)Ni(NO)(OTf) ——S41-S43
General details ——S41
Data collection ——S41
Structure solution and refinement —— S41
Table S6: Crystal data and structure refinement for (DIM)Ni(NO)(OTf) ——S42
Data for $(OBPin)_2Ni_2(NO)_2(Pz)$ ——S43-S45
General details ——S43
Data collection ——S43
Structure solution and refinement ——S43
Table S7: Crystal data and structure refinement for $(OBPin)_2Ni_2(NO)_2(Pz)$ ——S44
Figure S45: Structure of $(OBPin)_2Ni_2(NO)_2(Pz)$ ——S45
Data for $(OBPin)_2Ni_2(NO)_2(Bpy)$ ——S45-S47
General details ——S45
Data collection ——S45
Structure solution and refinement ——S46
Table S8: Crystal data and structure refinement for $(OBPin)_2Ni_2(NO)_2(Bpy)$ ——S46
Figure S46: Structure of $(OBPin)_2Ni_2(NO)_2(Bpy)$ ——S47

DFT
Figure S47: Calculated isomers of $(DIM)Ni(N_2O_2)$——S48
Figure S48: Corresponding orbitals and spin density for $[(DIM)Ni(NO)(ONO)]^-$——S49
Figure S49: Corresponding orbitals and spin density for triplet $(DIM)Ni(NO)_2$——S49

Experimental.

General. All manipulations were carried out under an atmosphere of ultra-high purity nitrogen using standard Schlenk techniques or in a glovebox under N_2. Solvents were purchased from commercial sources, purified using Innovative Technology SPS-400 PureSolv solvent system or by distilling from conventional drying agents and degassed by the freeze-pump-thaw method thrice prior to use. Glassware was oven-dried at 150 °C overnight and flame dried prior to use. Celite was dried under vacuum at 130 °C overnight.

NMR. ^1H NMR spectra were recorded in various deuterated solvents (dried over CaH_2) at 25 ^oC on a Varian Inova-400 or 500 spectrometer (^1H: 400.11 MHz, 500.11 MHz, respectively). ^{13}C and ^{15}N NMR data were recorded on a Varian Inova 500 spectrometer, and the ^{15}N NMR data was referenced to CH_3NO_2 δ = 0 ppm. Proton chemical shifts are reported in ppm versus solvent protic impurity and referenced to $SiMe_4$.

Mass Spectrometry. Mass spectrometry analyses were performed in an Agilent 6130 MSD (Agilent Technologies, Santa Clara, CA) quadrupole mass spectrometer equipped with a Multimode (ESI and APCI) source. MALDI-Mass Spectrometry was performed using a Bruker Autoflex III on $[(DIM)Ni(NO)]_2$ by first mixing the solid sample with the TCNQ matrix and steel beads in an Eppendorf tube, and the combined powders were tapped onto the MALDI target. The mass was externally calibrated using a mix of standard peptides as calibrants.

Infrared Spectroscopy. All IR spectra were collected using a Nicolet 6700. Gas phase IR spectra were collected with a custom made glass-IR cell with KBr salt plates pressed onto the cell using epoxy resin.

X-ray Photoelectron Spectroscopy. XPS spectra were recorded on a PHI Versa Probe II instrument equipped with monochromatic Al K(alpha) source. Instrument base pressure was ca. 1.8 × 10-10 Torr. The X-ray power of 65 W at 15 kV was used for all experiments with 260 micron beam size at X-ray incidence and take off angle of 45°. The spectrometer dispersion was adjusted to give a BE of 284.8 eV for the C 1s line of adventitious (aliphatic) carbon present. The PHI dual beam charge neutralization system was used during all measurements. The high resolution Ni 2p, O 1s and C 1s spectra were taken with a minimum of 10 - 60 scans using a 0.1 eV step and 23.5 eV pass energy; the HR N 1s using 46.95 eV pass energy. The ultimate Versa Probe II instrumental resolution was determined to be better than 0.125 eV using the Fermi edge of the valence band for metallic gold. The resolution with charge neutralization system was <0.68 eV FWHM on PET. All XPS spectra were recorded using PHI software SmartSoft –XPS v2.6.3.4 and processed using PHI MultiPack v9.3.0.3 and/or CasaXPS v.2.3.14. The XPS spectra were fitted using a combination of Gaussian and Lorentzian line shapes with 30-50% of Lorentzian contents. Shirley and/or iterated Shirley background was used to fit experimental data.

Reagents. $(DME)NiBr_2$ was purchased from Strem and used without further purification. $Na^{15}NO_2$ was purchased from Cambridge Isotope Laboratories and dried at 80 °C under vacuum overnight before use. $Ag^{15}NO_2$ was synthesized via salt metathesis of $Na^{15}NO_2$ and $AgNO_3$ in H_2O, and dried at 80 °C under vacuum overnight before use. $(Bpin)_2$, pyrazine, $AgNO_3$, and AgOTf were purchased from commercial sources and used without further purification. $(Bpin)_2Pz$ and $(Bpin)_2Bpy$ were synthesized according to established literature procedures.[1,2] Nitric oxide was synthesized on demand in a large Schlenk flask according to a literature procedure via the solid state reaction of $FeSO_4\bullet(H_2O)_7$ with $NaNO_2$ and used without further purification.[3]

Synthesis of (DIM)$NiBr_2$.

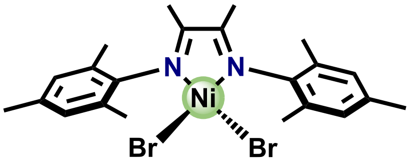

DIM (0.705 g, 2.22 mmol) was dissolved in DCM (5 mL) and added to a slurry of (DME)$NiBr_2$ (DME = dimethoxyethane, 0.684 g, 2.22 mmol) in 5 mL of DCM. This caused a color change to deep orange in 20 minutes. The reaction was stirred for 12 hours with no further color changes. All volatiles were removed under reduced pressure, giving rise to a deep orange solid, which was washed with 3 x 5mL diethyl ether. A crude orange powder was isolated in 82% yield.

^{1}H NMR of (DIM)$NiBr_2$: (400 MHz, CD_2Cl_2, 298K): δ(ppm) 35.12 (s, 6H), 28.10 (s, 12H), 24.21 (s, 4H), -21.31 (s, 6H).

Synthesis of (DIM)$Ni(NO_3)_2$.

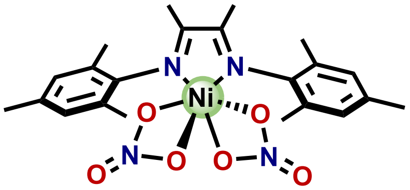

In a 20 mL scintillation vial, (DIM)$NiBr_2$ (0.0867 g, 0.161 mmol) was dissolved in 5 mL of acetonitrile. To this solution, silver nitrate (0.0574 g, 0.338 mmol, dissolved in 2 mL acetonitrile) was added dropwise, causing an immediate color change from orange to yellow-brown with immense precipitation of silver bromide. The reaction was stirred for 30 minutes with no additional color changes, upon which AgBr was filtered off giving rise to a homogenous yellow-brown solution. All volatiles were removed to give a tan powder, which was then redissolved in THF and filtered through a plug of celite. Removal of volatile material under reduced pressure gave rise to a pale yellow powder, isolated in 76% yield. Crystals suitable for single crystal X-ray diffraction were grown by diffusion of pentane vapors into a concentrated DCM solution.

^{1}H NMR of (DIM)$Ni(NO_3)_2$: (400 MHz, CD_2Cl_2, 298K): δ(ppm) 20.99 (s, 6H), 16.38 (s, 4H), 15.49 (s, 12H), -11.85 (s, 6H).
IR: $\mathbf{\nu}_{NO}$ = 1266 cm^{-1}, 1486 cm^{-1}
High Resolution Mass Spectrometry: (ESI+) m/z calculated: 440.15 m/z observed: 440.15 $[(DIM)Ni(NO_3)_2 - NO_3]^+$ m/z calculated: 944.28 m/z observed: 944.28 $[(DIM)_2Ni_2(NO_3)_4 - NO_3]^+$

Synthesis of (DIM)$Ni(NO_2)_2$.

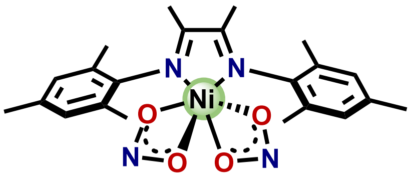

In a 20 mL scintillation vial, (DIM)$NiBr_2$ (0.0431 g, 0.080 mmol) was dissolved in 5 mL of acetonitrile. To this solution, silver nitrite (0.0258 g, 0.168 mmol, dissolved in 2 mL acetonitrile) was added dropwise, causing a slight color change to dark orange and immediate precipitation of byproduct silver bromide. The reaction was stirred for 30 minutes with no additional color changes, upon which AgBr was filtered off giving rise to a homogenous yellow-brown solution. All volatiles were removed to give a yellow powder, which was then redissolved in THF and filtered through a plug of celite. Removal of volatile material under reduced pressure gave rise to a dark yellow powder, isolated in 84% yield. Crystals suitable for single crystal X-ray diffraction were grown by diffusion of pentane vapors into a concentrated DCM solution.

Synthesis of (DIM)Ni($^{15}NO_2$)$_2$. In a 20 mL scintillation vial, (DIM)$NiBr_2$ was dissolved in 5 mL of dichloromethane. To this stirring solution was added $Na^{15}NO_2$ as a slurry in dichloromethane, as well as a catalytic amount of 18-crown-6 to help solubilize the sodium nitrite. After stirring for 48 hours, the resultant brown solution was filtered through a celite plug to remove the NaBr formed during the course of the metathesis. The filtrate was dried in vacuo to afford a yellow/brown solid, which was washed with pentane (2 x 3 mL) and ether (2 x 3 mL). The washed product was spectroscopically pure by ^{1}H NMR.

^{1}H NMR of (DIM)Ni(NO_2)$_2$: (400 MHz, d^3-MeCN, 298K): δ(ppm) 19.31 (s, 6H), 15.89 (s, 4H), 14.95 (s, 12H), -9.86 (s, 6H).
IR: ν_{NO} = 1211 cm^{-1}, 1476 cm^{-1}
High Resolution Mass Spectrometry: (ESI+) m/z calculated: 424.15 m/z observed: 424.15 [(DIM)Ni(NO_2)$_2$ – NO_2]$^+$ m/z calculated: 894.3 m/z observed: 894.3 [(DIM)$_2$Ni$_2$(NO_2)$_4$ – NO_2]$^+$

Synthesis of (DIM)Ni(NO)(ONO).

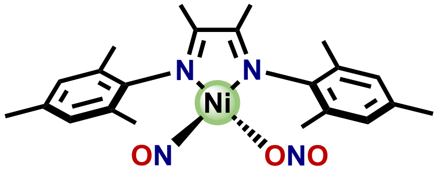

To a J-Young tube containing (DIM)Ni(NO_2)$_2$ (0.032 g, 0.068 mmol) in d_8-THF was added $(Bpin)_2$Pz (0.023 g, 0.068 mmol) also dissolved in d_8-THF. After heating for 6 hours at 45 °C, all starting material was consumed and one, new diamagnetic product was formed. The reaction was accompanied by a slight color change from brown to brown-green. All volatiles were removed in vacuo, and the green solid was rinsed with pentane (3 x 5 mL) to afford a green powder in 80% yield. Crystals suitable for single X-ray diffraction were grown by diffusion of pentane vapors into a concentrated THF solution.

Synthesis of (DIM)Ni(^{15}NO)(O^{15}NO). The same procedure above was followed for the synthesis of the isotopically labelled (DIM)Ni(^{15}NO)(O^{15}NO) complex, starting from isotopically labelled (DIM)Ni($^{15}NO_2$)$_2$ as the metal starting reagent.

^{1}H NMR of (DIM)Ni(NO_2)(NO): (400 MHz, d_8-THF, 298K): δ(ppm) 7.03 (s, 4H), 2.34 (s, 6H), 2.16 (s, 12H), 2.10 (s, 6H).
^{15}N NMR of (DIM)Ni(^{15}NO)(O^{15}NO): (400 MHz, d_8-THF, 298K): δ(ppm) 5.39 (s, 1N)
^{15}N NMR of (DIM)Ni(^{15}NO)(O^{15}NO): (400 MHz, CD_2Cl_2, 231K): δ(ppm) -19.05 (s, 1N), 216.18 (s, 1N)

IR: $\mathbf{v}_{NO}$ = 1785 cm^{-1}, $\mathbf{v}_{NO}$ = 1745 cm^{-1} for (DIM)Ni(NO)(O^{15}NO)
High Resolution Mass Spectrometry: (ESI+) m/z calculated: 408.15 m/z observed: 408.15 [(DIM)Ni(NO)(ONO) – NO_2]$^+$ m/z calculated: 862.3 m/z observed: 862.3 [(DIM)$_2$Ni$_2$(NO)$_2$(ONO)$_2$ – NO_2]$^+$

Synthesis of [(DIM)Ni(NO)]$_2$.

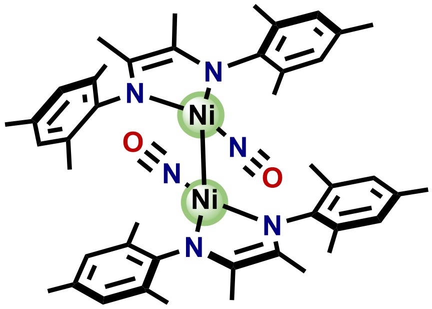

To a J-Young tube containing (DIM)Ni(NO)(NO_2) (0.027 g, 0.06 mmol) in d$_8$-THF was added (Bpin)$_2$Pz (0.039 g, 0.12 mmol) also dissolved in d$_8$-THF. After spinning for 12 hours at room temperature, all starting material was consumed and free pyrazine and boryl ether are observed by ^{1}H NMR. The product crystallizes while being agitated in an NMR tube. The crystalline product was isolated in 75% yield by pouring out the mother liquor, and rinsing the dark solid with pentane and ether. [(DIM)Ni(NO)]$_2$ is remarkably insoluble, and crashes out as crystalline product when the reaction is performed in dichloromethane, acetonitrile, and toluene as well. Independent X-ray crystal structures were obtained from a THF reaction and a DCM reaction, of which the DCM reaction structure gives better data and is the one reported in this manuscript.

Synthesis of [(DIM)Ni(^{15}NO)]$_2$. The same procedure above was followed for the synthesis of the isotopically labelled [(DIM)Ni(^{15}NO)]$_2$, starting from isotopically labelled (DIM)Ni(^{15}NO)(O^{15}NO) as the metal starting reagent.

IR: $\mathbf{v}_{NO}$ = 1664 cm^{-1} for [(DIM)Ni(NO)]$_2$ and 1630 cm^{-1} for [(DIM)Ni(^{15}NO)]$_2$
High Resolution MALDI-TOF mass spectrometry: (ESI+) m/z calculated: 816.3 m/z observed: 816.9 {[(DIM)Ni(NO)]$_2$}$^+$ and m/z calculated: 800.32 m/z observed: 800.3 {[(DIM)Ni(NO)]$_2$-O}$^+$.

Synthesis of (DIM)Ni(NO)(OTf)

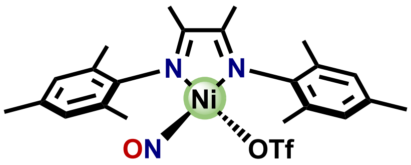

A slurry of freshly prepared [(DIM)Ni(NO)]$_2$ in THF was filtered through a celite plug, upon which the [(DIM)Ni(NO)]$_2$ precipitate stayed on top of the celite pad. THF (2 x 3 mL) and

acetonitrile (2 x 3 mL) were pushed through the celite plug to wash any leftover organic byproducts from the synthesis of [(DIM)Ni(NO)]$_2$. The THF and acetonitrile washings filtered through the celite plug were colorless. An equimolar solution of AgOTf in 5 mL of acetonitrile was prepared, the AgOTf solution was filtered through [(DIM)NiNO]$_2$ on the celite plug, and the resulting filtrate was green. The reduced silver was seen on the top of the celite plug, and the filtrate was dried in vacuo. The resultant green solid was rinsed with pentane (2 x 3 mL), and crystallized in 71% yield from vapor diffusion of pentane into a concentrated THF solution containing (DIM)Ni(NO)(OTf).

^{1}H NMR of (DIM)Ni(NO)(OTf): (400 MHz, d_8-THF, 298K): δ(ppm) 7.26 (s, 4H), 2.48 (s, 6H), 2.23 (s, 18H).
IR: $\boldsymbol{\nu}_{NO}$ = 1778 cm^{-1} (KBr), $\boldsymbol{\nu}_{NO}$ = 1825 cm^{-1} (solution, CH_2Cl_2), $\boldsymbol{\nu}_{NO}$ = 1778 cm^{-1} (solution, THF)
High Resolution Mass Spectrometry: (ESI+) m/z calculated: 408.15 m/z observed: 408.15 [(DIM)Ni(NO)(OTf) – OTf]$^+$

Synthesis of (DIM)Ni(NO)(NO_3)

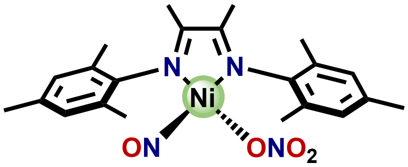

A sample of [(DIM)Ni(NO)]$_2$ was isolated on top of a celite plug in the same way reported above. An equimolar solution of $AgNO_3$ in 5 mL of acetonitrile was prepared, the $AgNO_3$ solution was filtered through the celite plug carrying [(DIM)NiNO]$_2$, and the resulting filtrate was light brown. The oxidized silver was seen on the top of the celite plug, and the filtrate was dried in vacuo to afford a green solid. The solid was rinsed with both pentane (2 x 3 mL) and ether (2 x 3 mL), and redissolved in a minimal amount of THF. To this concentrated solution was added pentane to precipitate a fine green powder in 68% yield.

^{1}H NMR of (DIM)Ni(NO)(NO_3): (400 MHz, d_8-THF, 298K): δ(ppm) 7.03 (s, 4H), 2.32 (s, 6H), 2.25 (s, 12H), 2.03 (s, 6H).
IR: $\boldsymbol{\nu}_{NO}$ = 1776 cm^{-1} (KBr)
High Resolution Mass Spectrometry: (ESI+) m/z calculated: 408.15 m/z observed: 408.15 [(DIM)Ni(NO)(NO_3) – NO_3]$^+$ m/z calculated: 878.3 m/z observed: 878.3 [(DIM)$_2$Ni$_2$(NO)$_2$(NO_3)$_2$ – NO_3]$^+$

Synthesis of (DIM)Ni(^{15}NO)(ONO) and (DIM)Ni(NO)(O^{15}NO)

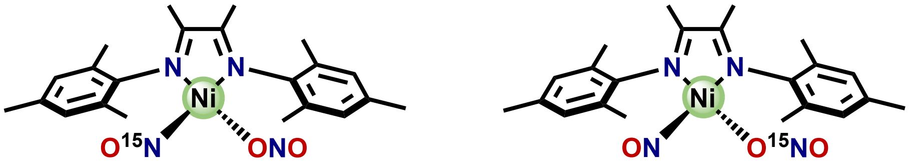

For the synthesis of (DIM)Ni(NO)(O^{15}NO), a sample of [(DIM)Ni(NO)]$_2$ was isolated on top of a celite plug as reported above. An equimolar solution of Ag$^{15}NO_2$ in 5 mL of acetonitrile was prepared, the Ag$^{15}NO_2$ solution was filtered through the celite plug, and the resulting filtrate was light brown. The oxidized silver was seen on the top of the celite plug, and the filtrate was dried in vacuo to afford a green solid. The solid was rinsed with both pentane (2 x 3 mL) and ether (2 x 3 mL), and redissolved in a minimal amount of THF. To this concentrated solution was added pentane to precipitate a fine green powder in 84% yield.

The synthesis of the other isotopomer, (DIM)Ni(^{15}NO)(ONO), followed the same experimental procedure as the synthesis of (DIM)Ni(NO)(O^{15}NO). However, the incorporation of ^{15}N at the nitrosyl position requires the starting dimer to be isotopically labelled: [(DIM)Ni(^{15}NO)]$_2$. To the isotopically labelled dimer was added equimolar unlabelled $AgNO_2$ to afford the corresponding (DIM)Ni(^{15}NO)(ONO) compound in 79% yield.

Synthesis of (OBPin)$_2$Ni$_2$(NO)$_2$(Pz).

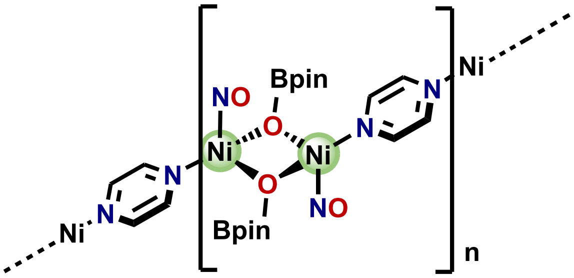

To a J-Young tube containing (DIM)Ni(NO_2)$_2$ (0.035 g, 0.074 mmol) in d$_8$-THF was added (Bpin)$_2$Pz (0.049 g, 0.148 mmol) also dissolved in d$_8$-THF. After heating for 3 hours at 80 °C, all starting material was consumed, and the solution changed color from brown to blue. Upon cooling to room temperature, blue crystalline precipitate forms in the J-Young tube. The solution is subsequently filtered in the glovebox through celite, upon which the crystalline precipitate stays at the top of the celite. The product was rinsed with pentane and ether through the celite to ensure removal of all organic byproducts. The crystalline product was washed through the celite using dichloromethane, and all volatiles were removed to afford a blue solid. This blue solid was washed again with pentane (3 x 5 mL), to afford the product as a blue solid isolated in 73% yield.

^{1}H NMR of (OBPin)$_2$Ni$_2$(NO)$_2$(Pz): (400 MHz, CD_2Cl_2, 298K): δ(ppm) 8.9 (bs, 4H), 1.5 (s, 24H)
IR: ν_{NO} = 1810 cm^{-1}

Synthesis of (OBPin)$_2$Ni$_2$(NO)$_2$(Bpy).

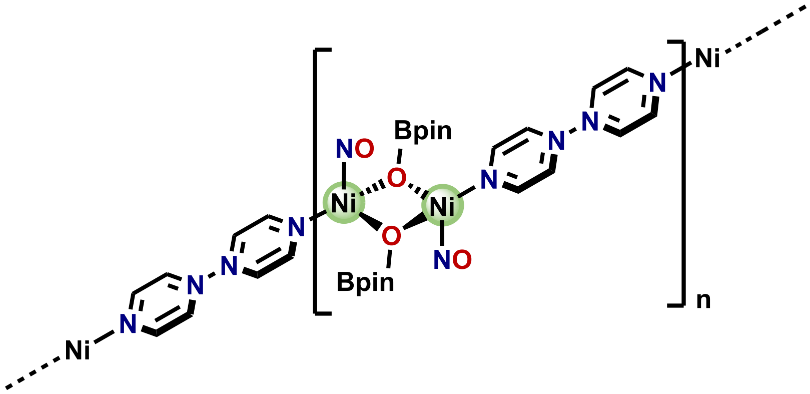

To a J-Young tube containing (DIM)Ni(NO_2)$_2$ (0.035 g, 0.074 mmol) in d_8-THF was added (Bpin)$_2$Bpy (0.069 g, 0.148 mmol) also dissolved in d_8-THF. After spinning for 5 hours at room temperature, all starting material was consumed, and the solution changed color from brown to blue. After taking the NMR tube off the spinner blue crystalline precipitate forms in the J-Young tube. The solution is subsequently filtered in the glovebox through celite, upon which the crystalline precipitate stays at the top of the celite. The product was rinsed with pentane and ether through the celite to ensure removal of all organic byproducts. The low solubility of the bipyridine based polymer precluded the collection of a ^{1}H NMR spectrum.

Experimental procedure for detection of nitrous oxide after (OBPin)$_2$Ni$_2$(NO)$_2$(Bpy) and (OBPin)$_2$Ni$_2$(NO)$_2$(Pz) formation. Both (OBPin)$_2$Ni$_2$(NO)$_2$(Pz) and (OBPin)$_2$Ni$_2$(NO)$_2$(Bpy) were synthesized according to the established experimental procedure above, but the reactions were carried out in a sealed Schlenk flask to allow for accumulation of gaseous byproducts. Once the polymeric compounds had formed, the Schlenk flask was attached to a gas manifold, which also contained an evacuated gas IR cell. The entire gas manifold was evacuated except the headspace of the reaction flask. The manifold was closed from the vacuum, and a condensation arm built on the side of the gas IR cell was placed in liquid nitrogen. The reaction flask was opened to the static vacuum, allowing any gaseous byproducts formed during the reaction to condense into the gas IR cell for subsequent spectroscopy.

Experimental procedure for detection of nitrous oxide using gas IR following deoxygenation of (DIM)Ni(NO)(ONO). The deoxygenation of (DIM)Ni(NO)(ONO) was performed using (Bpin)$_2$Pz in a sealed Schlenk flask, according to the experimental procedure above. The solution was stirred overnight at room temperature to ensure complete consumption of starting materials. Once the reaction was complete, the Schlenk flask containing the reaction mixture was attached to a gas manifold, and the transfer of gaseous byproducts into the gas IR cell was performed according to the general procedure outlined above.

Experimental procedure for detection of nitrous oxide using solution IR following deoxygenation of (DIM)Ni(NO)(ONO). To a solution of (DIM)Ni(NO)(ONO) dissolved in THF in a scintillation vial was added (Bpin)$_2$(Bpy), also dissolved in THF. This solution was mixed using a pipette 5 times, before being quickly transferred to a solution IR cell. The solution was then periodically monitored by IR, showing N_2O formation at the earliest scan, with little change in the

intensity of the N_2O bands during the course of the reaction due to saturation of the THF solution.

Experimental procedure for oxidation of (DIM)Ni(NO)(X) (X = NO_2^- or NO_3^-) and detection of liberated nitric oxide. To an acetonitrile solution of either (DIM)Ni(NO)(ONO) or (DIM)Ni(NO)(ONO_2) was added $AgNO_2$ or $AgNO_3$, respectively, also in acetonitrile in a 1:1 mole ratio. This solution was placed in a sealed Schlenk flask, and the resultant solution was stirred at room temperature overnight. The nitric oxide can be detected via vacuum transfer of the volatiles to a gas IR cell as outlined by the general procedure above. After detection of nitric oxide, the reaction solutions are dried in vacuo to afford a brown/purple solid. The solids were washed with pentane (2 x 3 mL) and ether (2 x 3 mL) to afford spectroscopically pure (DIM)Ni(NO_2)$_2$ or (DIM)Ni(NO_3)$_2$ as judged by ^{1}H NMR spectroscopy.

Experimental procedure for nitric oxide disproportionation with a slurry of [(DIM)Ni(NO)]$_2$. A sample of dimeric [(DIM)Ni(NO)]$_2$ was isolated and suspended as a slurry in THF. This slurry was placed in a Schlenk flask and transferred to the gas manifold. After two freeze-pump-thaw cycles, excess nitric oxide was condensed into the Schlenk flask containing the frozen slurry placed in a liquid nitrogen bath. After the addition of nitric oxide and upon warming to room temperature, the slurry became a homogenous green/brown solution. After stirring for 30 minutes, the volatiles from the reaction flask were vacuum transferred to a gas IR cell according to the general procedure outlined above, showing N_2O formation as well as excess nitric oxide. The reaction solution was dried in vacuo, and the green solid was washed with pentane (2 x 3 mL) and ether (2 x 3 mL) to afford spectroscopically pure (DIM)Ni(NO)(ONO) as judged by ^{1}H NMR spectroscopy and the diagnostic v_{NO} stretching frequency.

Experimental procedure for nitric oxide disproportionation with [(DIM)Ni(NO)]$_2$ as a solid powder. A sample of dimeric [(DIM)Ni(NO)]$_2$ was isolated and mixed with dry KBr. The dimer/KBr solid mixture was transferred to a Schlenk flask and transferred to the gas manifold. After evacuation of the Schlenk flask, two equivalents of nitric oxide were condensed into the Schlenk flask placed in a liquid nitrogen bath. After warming to room temperature and allowing the flask to stay at room temperature for 20 minutes, the Schlenk flask was evacuated, the flask was transferred into the glovebox, and a sample of the dimer/KBr mixture that had been exposed to nitric oxide was used to make a KBr disc for IR spectroscopy. The Schlenk flask was returned to the gas manifold and two more equivalents of nitric oxide were condensed into the Schlenk flask. 30 minutes after the addition of the third and fourth equivalents of nitric oxide, the Schlenk flask was once again evacuated and brought back into the glovebox, upon which an aliquot of the KBr mixture was used for collection of a second IR spectrum. The process of NO addition and assay by solid state infrared spectroscopy was repeated two more times (4 times total to give an 8:1 mole ratio of NO to dimer). No intermediates were seen by solid state IR spectroscopy, and the only diagnostic change observed was the growth of v_{NO} for (DIM)Ni(NO)(ONO).

Spectral data.

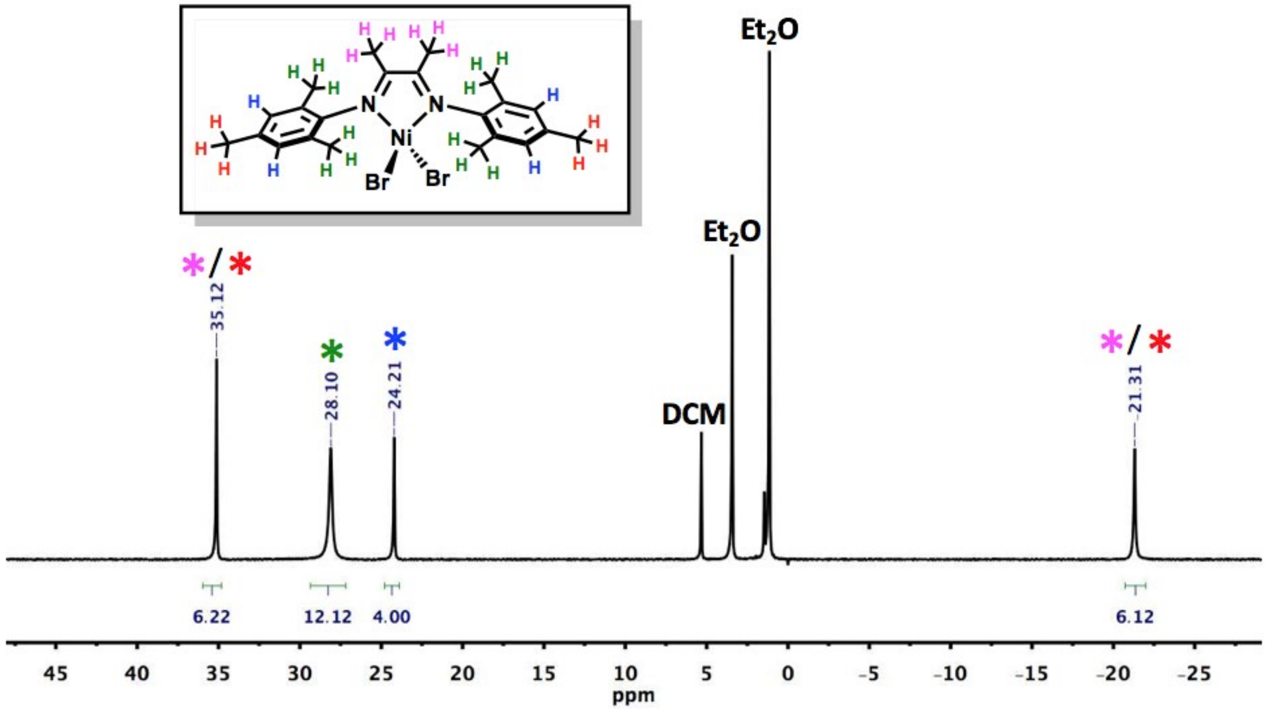

Figure S1: ^{1}H NMR (400 MHz, CD_2Cl_2, 298K) of (DIM)$NiBr_2$

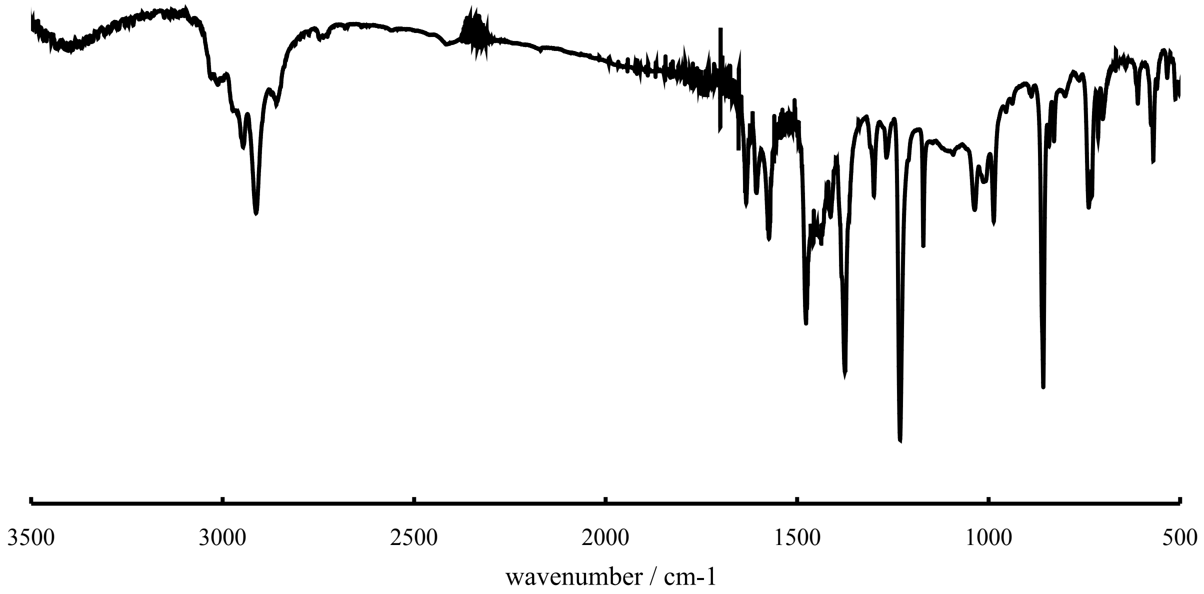

Figure S2: FT-IR spectrum of (DIM)$NiBr_2$

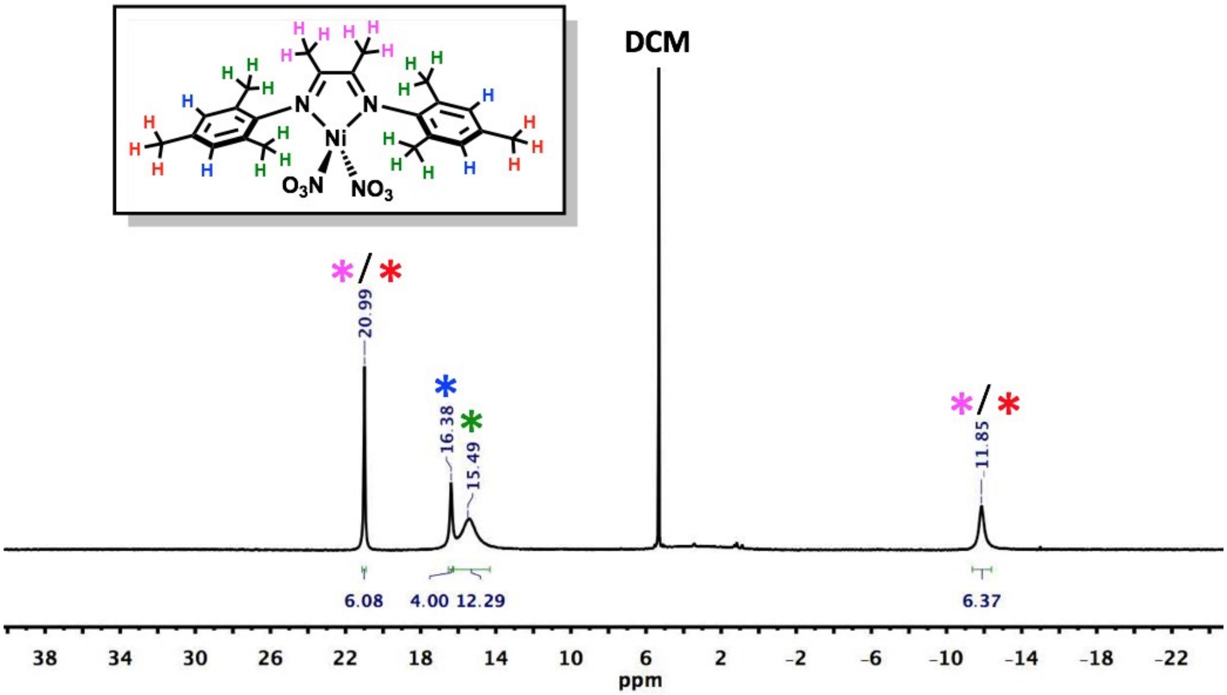

Figure S3a: ^{1}H NMR (400 MHz, CD_2Cl_2, 298K) of (DIM)Ni(NO_3)$_2$ (nitrate ligands drawn as monodentate here for simplicity)

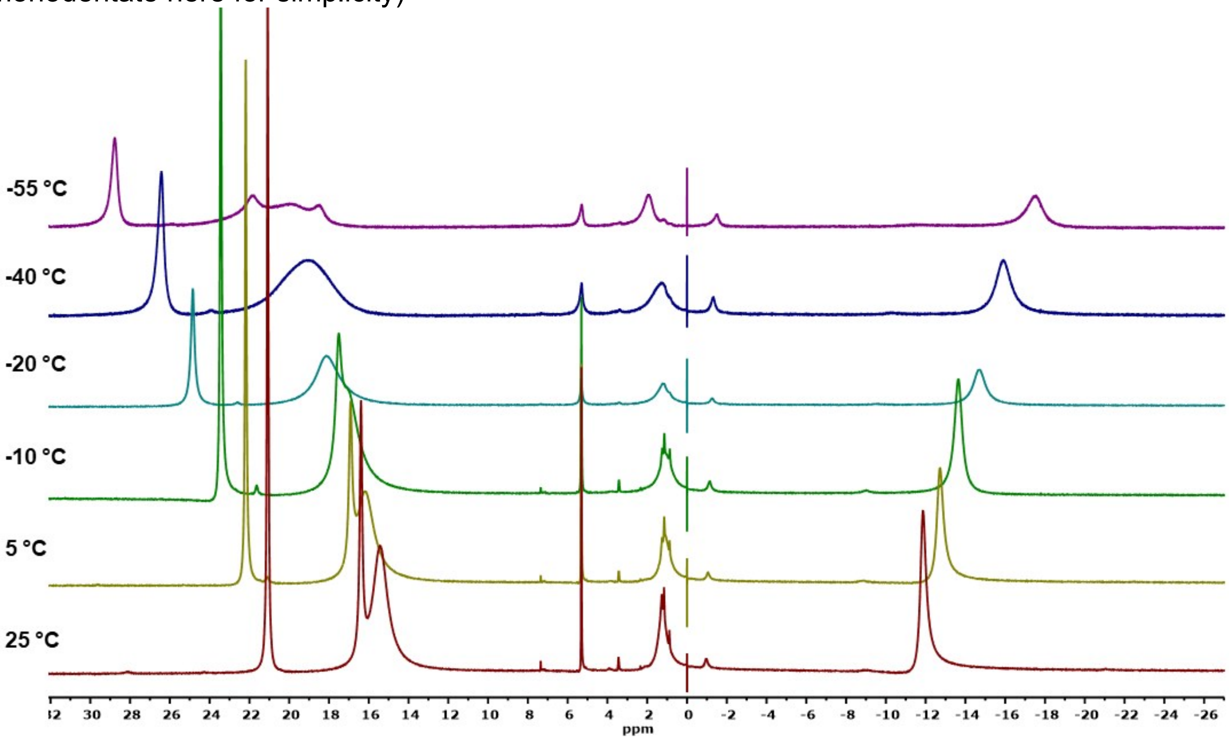

Figure S3b: Variable temperature ^{1}H NMR of (DIM)Ni(NO_3)$_2$. See Figure S5b for a discussion of the VT NMR data.

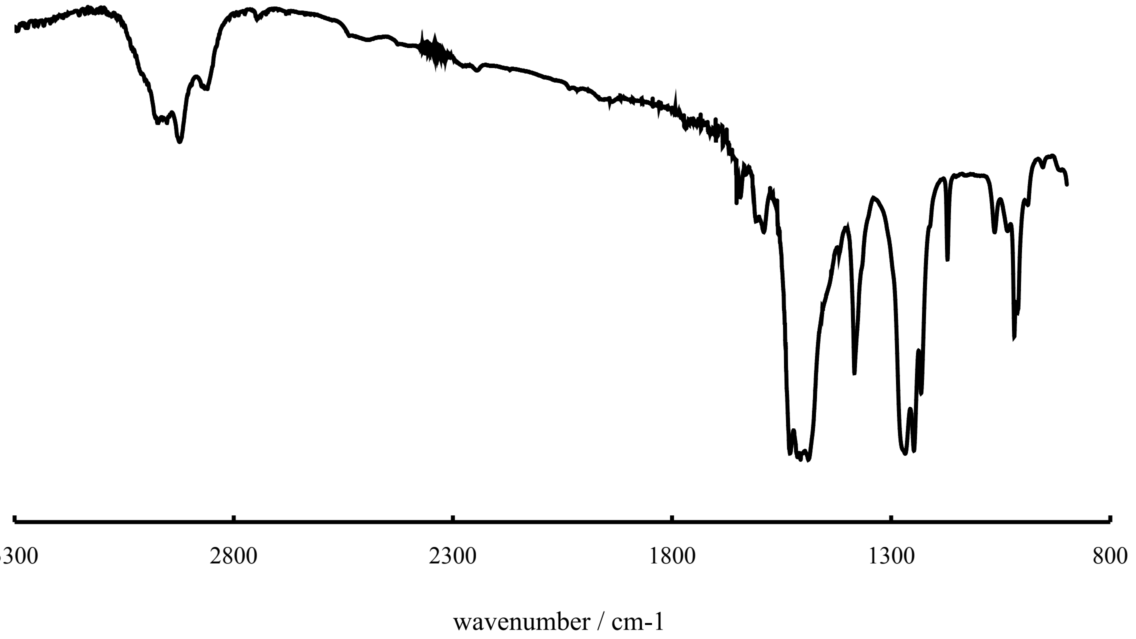

Figure S4: FT-IR spectrum of (DIM)Ni(NO_3)$_2$

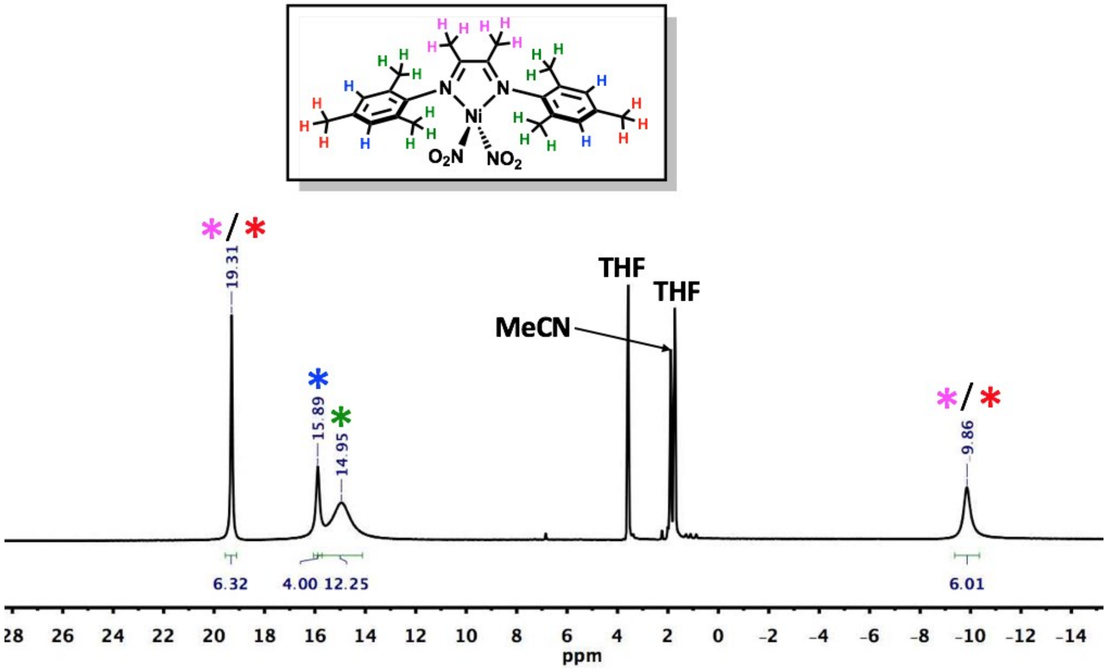

Figure S5a: ^{1}H NMR (400 MHz, d^3-MeCN, 298K) of (DIM)Ni(NO_2)$_2$ (nitrite ligands drawn as monodentate here for simplicity)

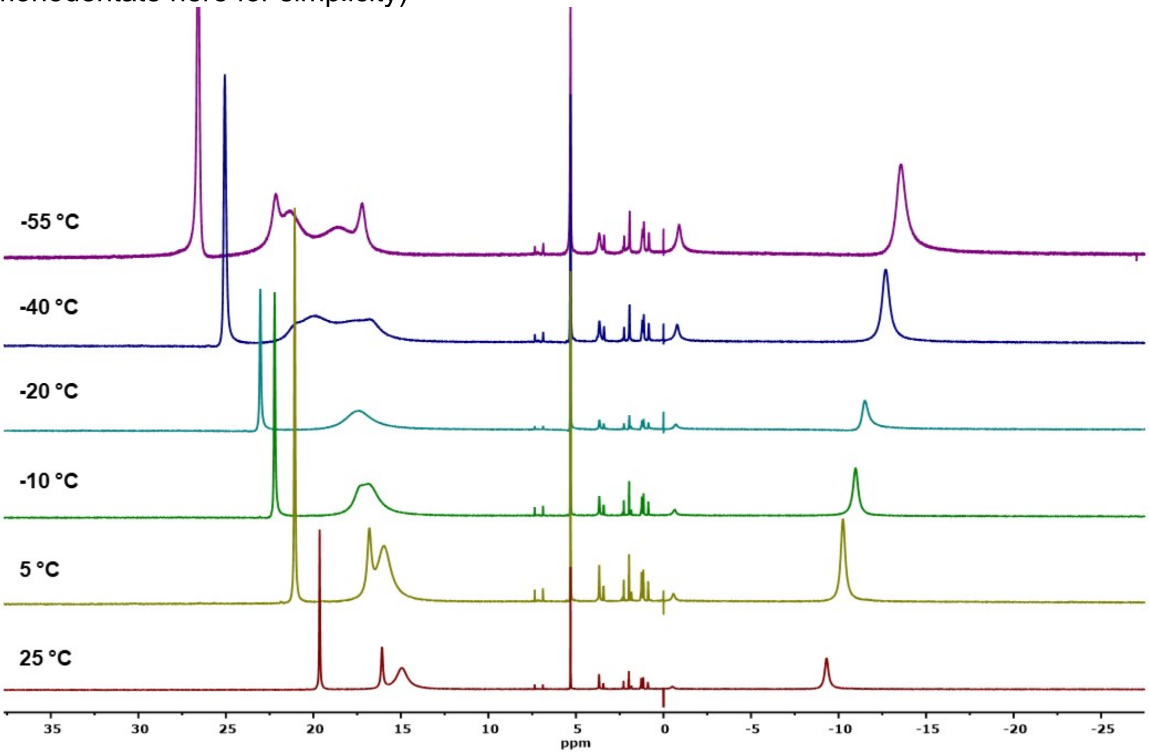

Figure S5b: Variable temperature ^{1}H NMR of (DIM)Ni(NO_2)$_2$. Upon cooling, the signals for the ortho -CH_3 on the mesityl arm and the meta C-H proton (as judged by relative integrations) both shift downfield; however, the -CH_3 has a larger shift. This leads to accidental degeneracy, best seen at -20 °C. Further cooling alleviates this accidental degeneracy, but also begins to split the ortho -CH_3 and meta C-H signals into two signals each. This can be attributed to decreased rapid rotation of the mesityl groups at lower temperatures, leading to inequivalent ortho -CH_3 and meta C-H groups on each mesityl arm. The lack of rapid rotation does not affect the para -CH_3 groups, or the -CH_3 on the DIM backbone, so these signals do not split. This same pattern is observed for the bis-nitrate complex; however, it is more well resolved in the bis-nitrite.

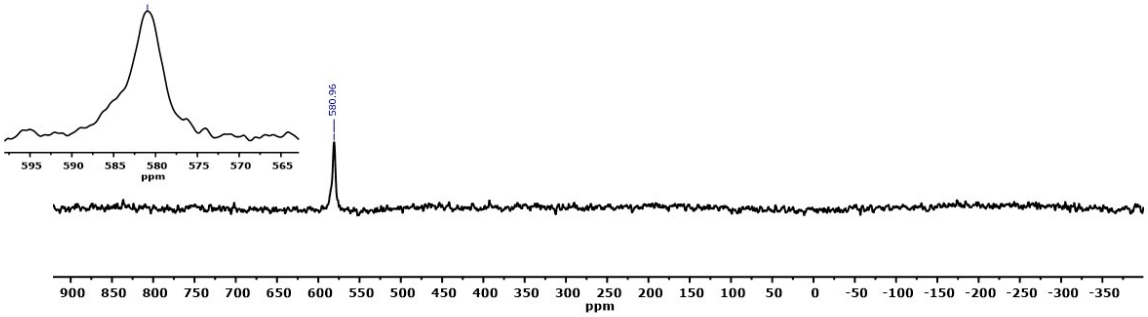

Figure S6. ^{15}N NMR spectrum of (DIM)Ni(NO_2)$_2$ in CD_2Cl_2

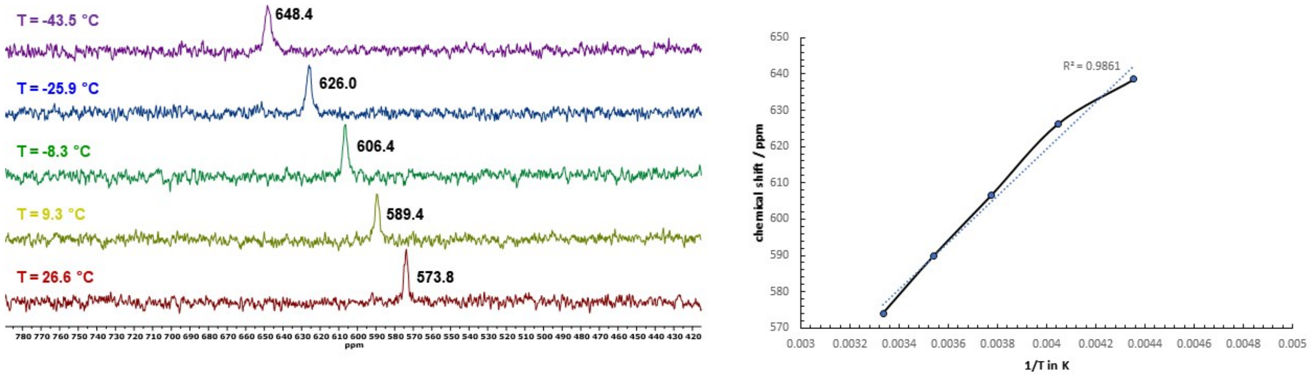

Figure S7. Variable temperature ^{15}N NMR spectra of (DIM)Ni(NO_2)$_2$ in CD_2Cl_2 showing a strong dependence of temperature on chemical shift (left) and Curie-Weiss plot showing linear dependence of the chemical shift of the paramagnetic compound with respect to inverse temperature (right)

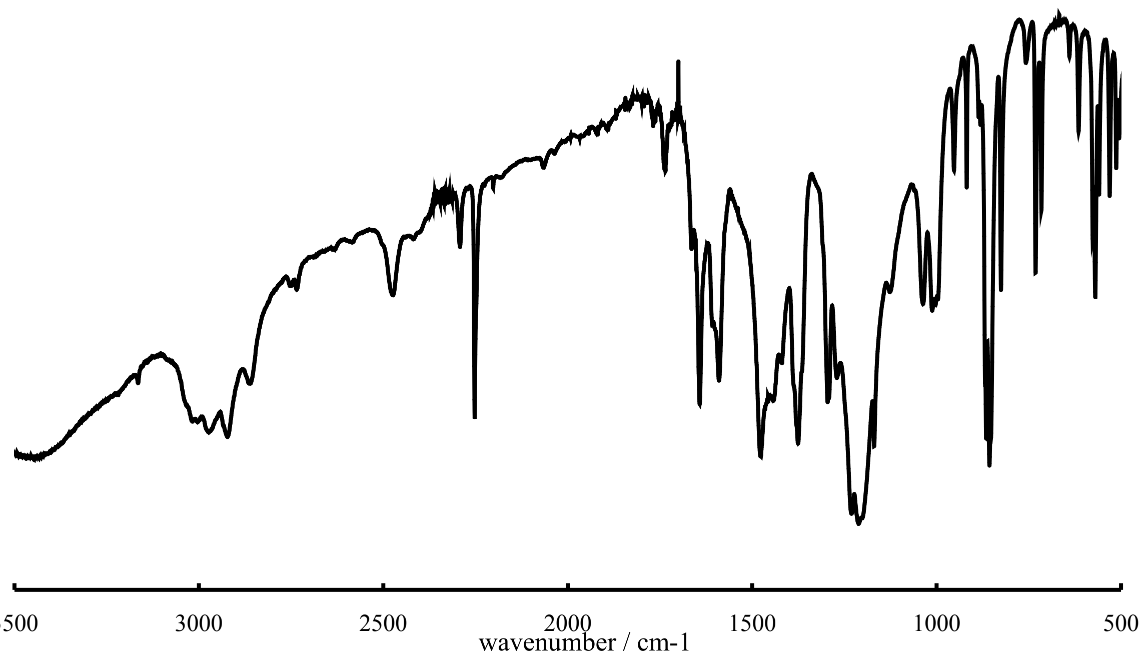

Figure S8: FT-IR spectrum of (DIM)Ni(NO_2)$_2$

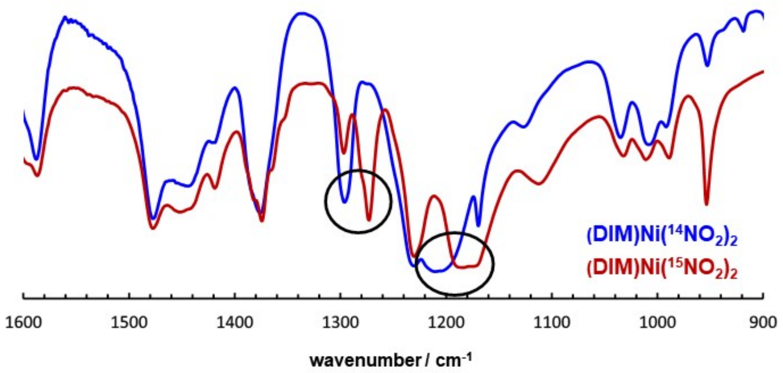

Figure S9. FT-IR spectral overlay of isotopically labelled (DIM)Ni($^{15}NO_2$)$_2$ with unlabelled (DIM)Ni(NO_2)$_2$, showing diagnostic shifts of the nitrite stretches

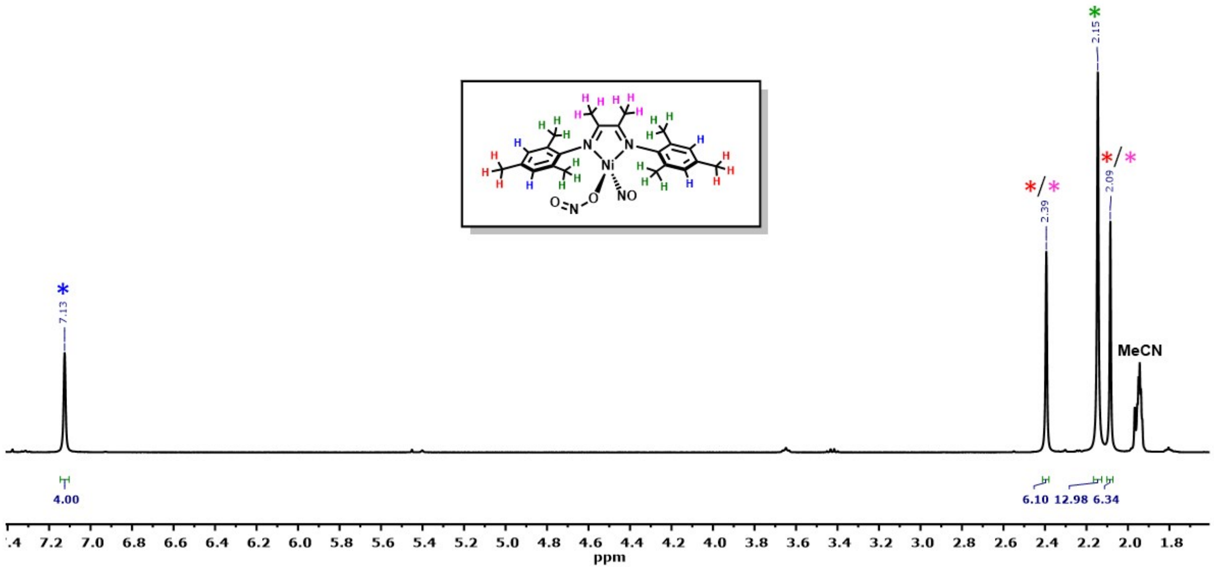

Figure S10. ^{1}H NMR spectrum of (DIM)Ni(NO)(ONO) in CD_3CN

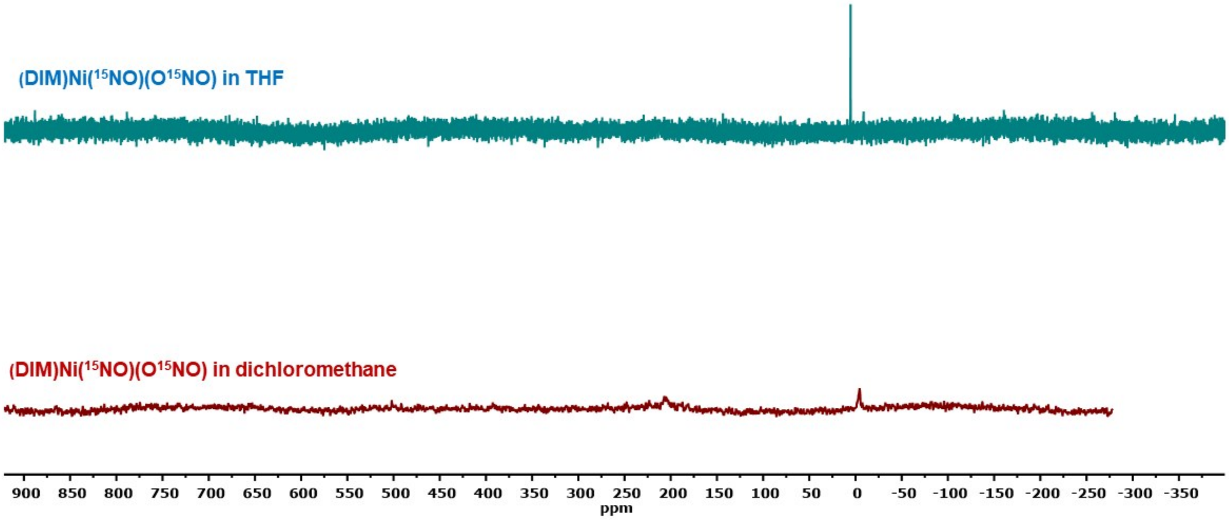

Figure S11. ^{15}N NMR spectrum of (DIM)Ni(^{15}NO)($O^{15}NO$) in dichloromethane (bottom, red) and THF (blue, top). One signal was observed in THF, the same signal was observed in dichloromethane, along with a broad, low intensity signal at 206 ppm. The broad, low intensity signal is well resolved at -42 °C (Manuscript, Figure 5b). The coordinating solvent increases the rate of nitrito ligand exchange.

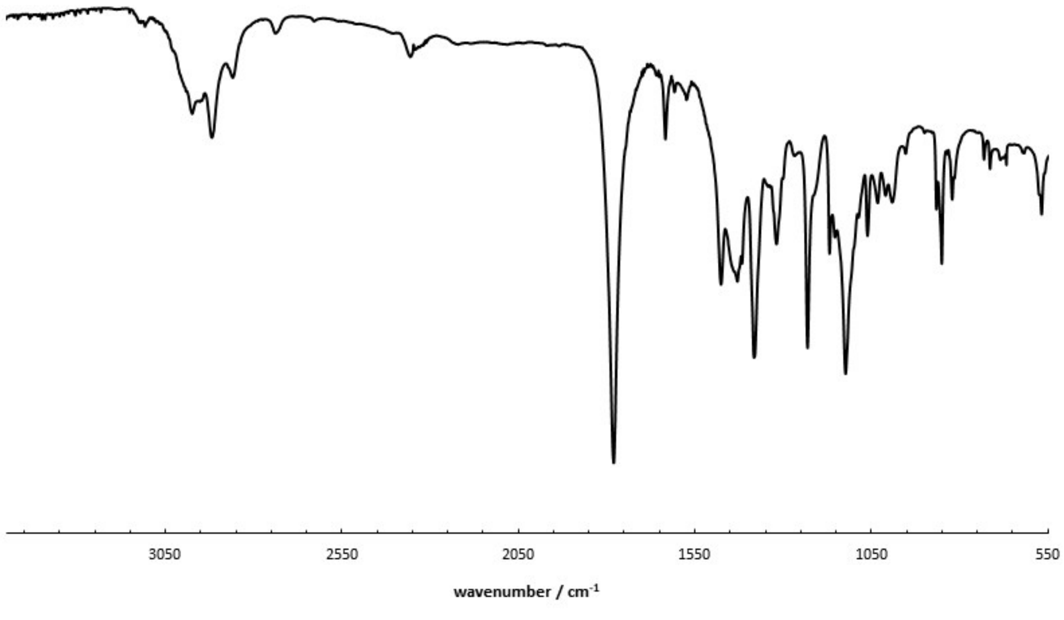

Figure S12. IR spectrum (KBr) of (DIM)Ni(NO)(ONO)

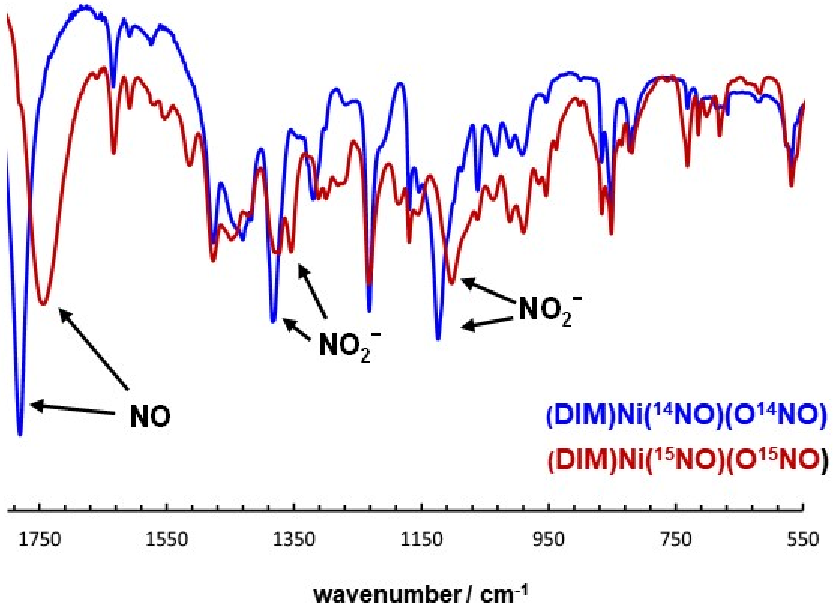

Figure S13. FT-IR spectral overlay of isotopically labelled (DIM)Ni(^{15}NO)(O^{15}NO) with unlabelled (DIM)Ni(NO)(ONO), showing the diagnostic shifts for the isotopically labelled compound nitrosyl and nitrite stretches

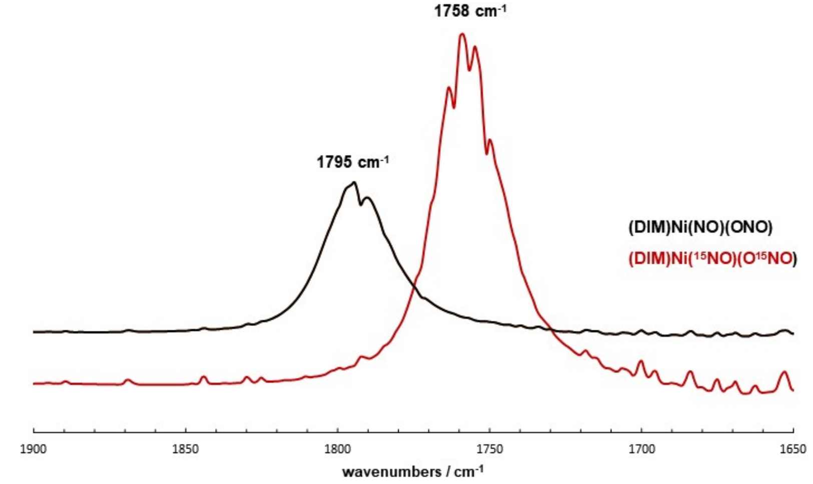

Figure S14. Solution phase FT-IR spectra overlay in dichloromethane of the nitrosyl region for isotopically labelled (DIM)Ni(^{15}NO)(O^{15}NO) and unlabelled (DIM)Ni(NO)(ONO)

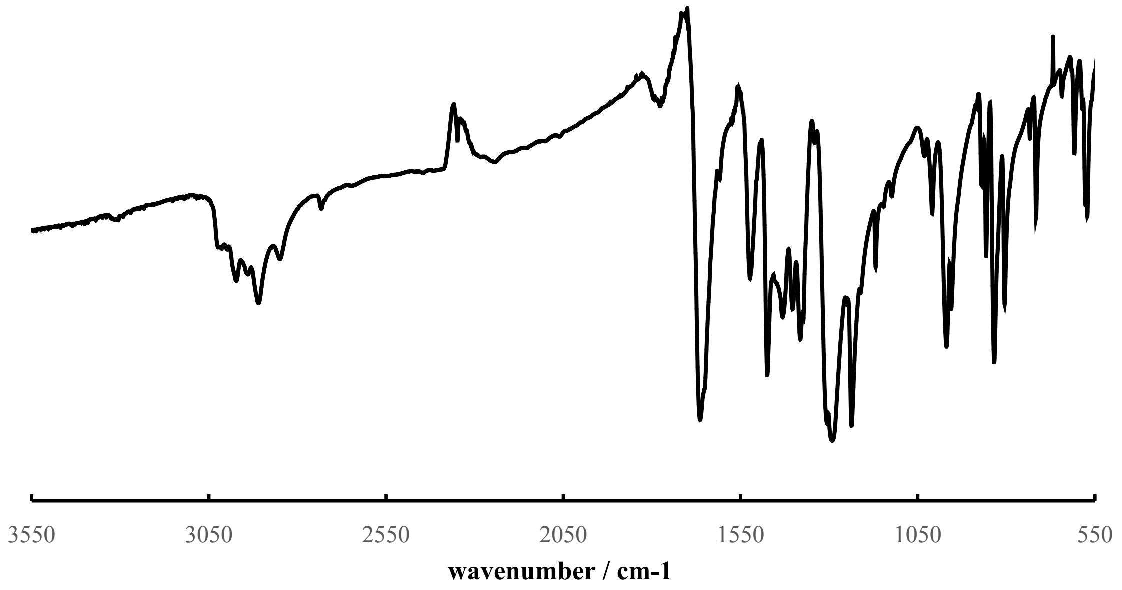

Figure S15: FT-IR spectrum of [(DIM)Ni(NO)]$_2$

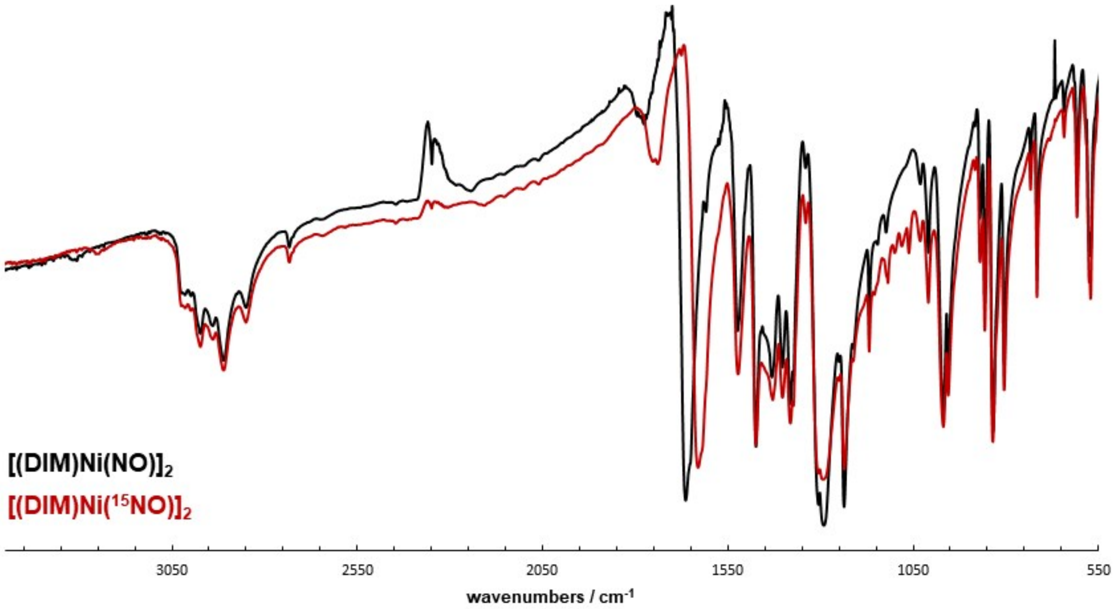

Figure 16. FT-IR spectrum of [(DIM)Ni(NO)]$_2$ overlaid with isotopically labelled [(DIM)Ni(^{15}NO)]$_2$

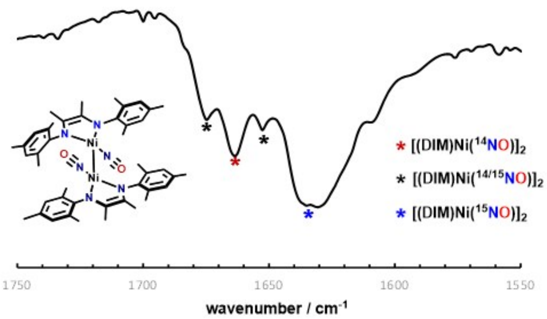

	DFT	Experiment
14/14	1707	1664
15/14	1711<br>1675	1675<br>1652
15/15	1670	1633

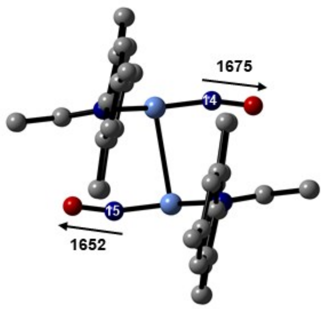

Figure S17. FT-IR spectrum of dimeric $[(DIM)Ni(NO)]_2$ synthesized from deoxygenation of $(DIM)Ni(NO)(O^{15}NO)$, showing three isotopomers of the dimer: $[(DIM)Ni(^{14}NO)]_2$, $[(DIM)Ni(^{15}NO)]_2$, and $[(DIM)Ni(^{14/15}NO)]_2$. The mixed labelled dimer, $[(DIM)Ni(^{14/15}NO)]_2$, has two distinct nitrosyl stretches due to the rigorous symmetry of the dimer being broken. The shifts of the experimental isotopomers are in good agreement with the calculated IR stretches, which predicts that the ^{14}N nitrosyl in $[(DIM)Ni(^{14/15}NO)]_2$, should be at a higher energy stretching frequency than the unlabelled $[(DIM)Ni(^{14}NO)]_2$ complex, consistent with experiment.

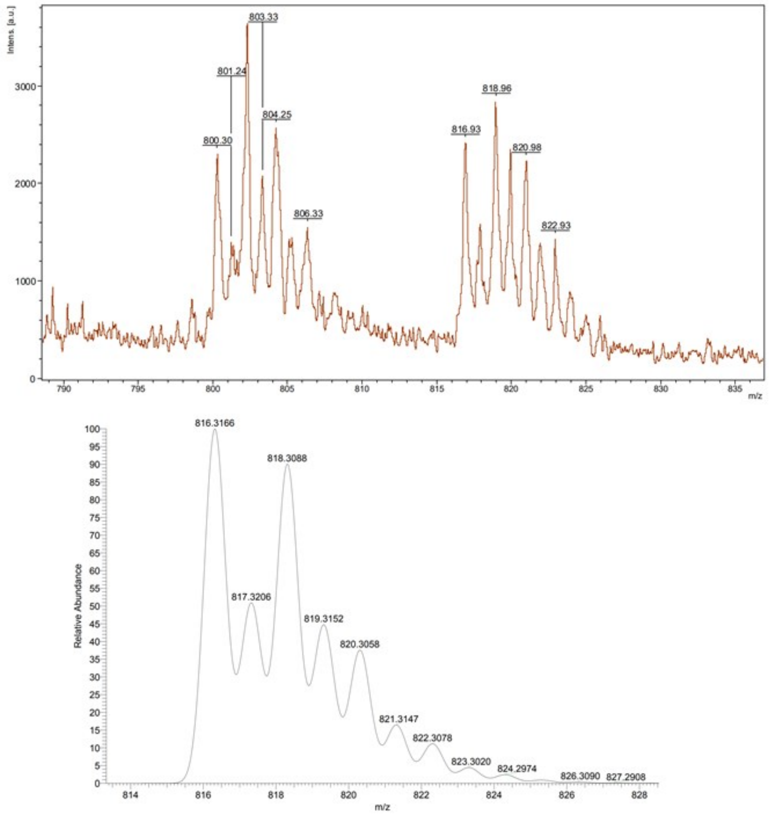

Figure S18. MALDI-TOF spectrum of $[(DIM)Ni(NO)]_2$ (top) with simulated spectrum (bottom) for the intact molecular ion. The m/z at 800.30 in the experimental spectrum corresponds to $[(DIM)Ni(NO)]_2$-O^+, indicating the loss of an oxygen from the dimer.

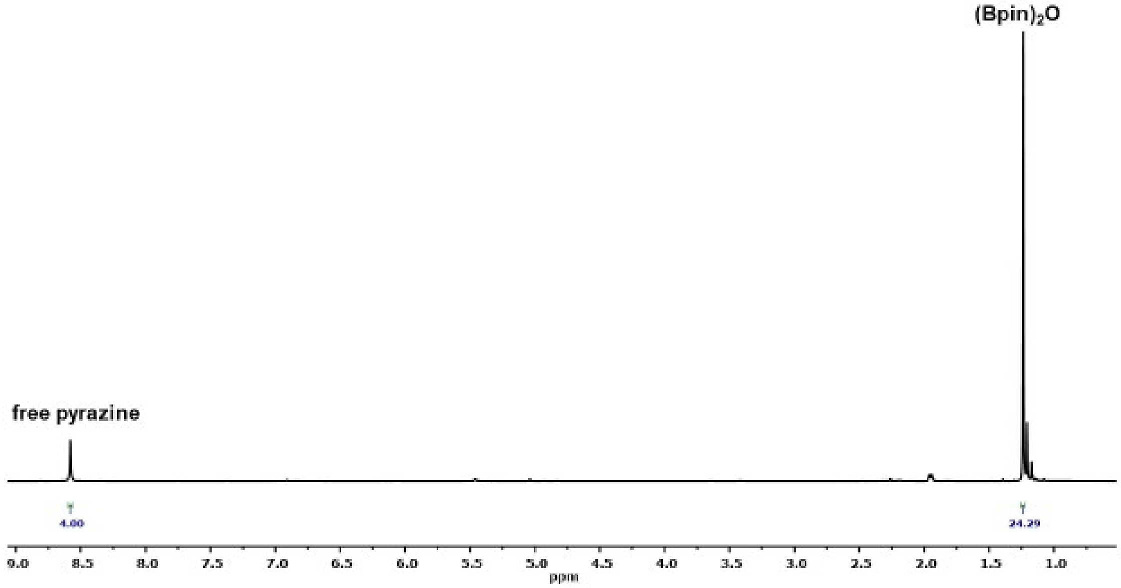

Figure S19. Reaction of (DIM)Ni(NO)(NO_2) with (Bpin)$_2$Pz in a 2:3 mole ratio in CD_3CN to form [(DIM)Ni(NO)]$_2$. All starting material has been consumed, with no excess borylating reagent present. All [(DIM)Ni(NO)]$_2$ has precipitated from solution, leaving only pyrazine and boryl ether in the ^{1}H NMR in an equimolar ratio.

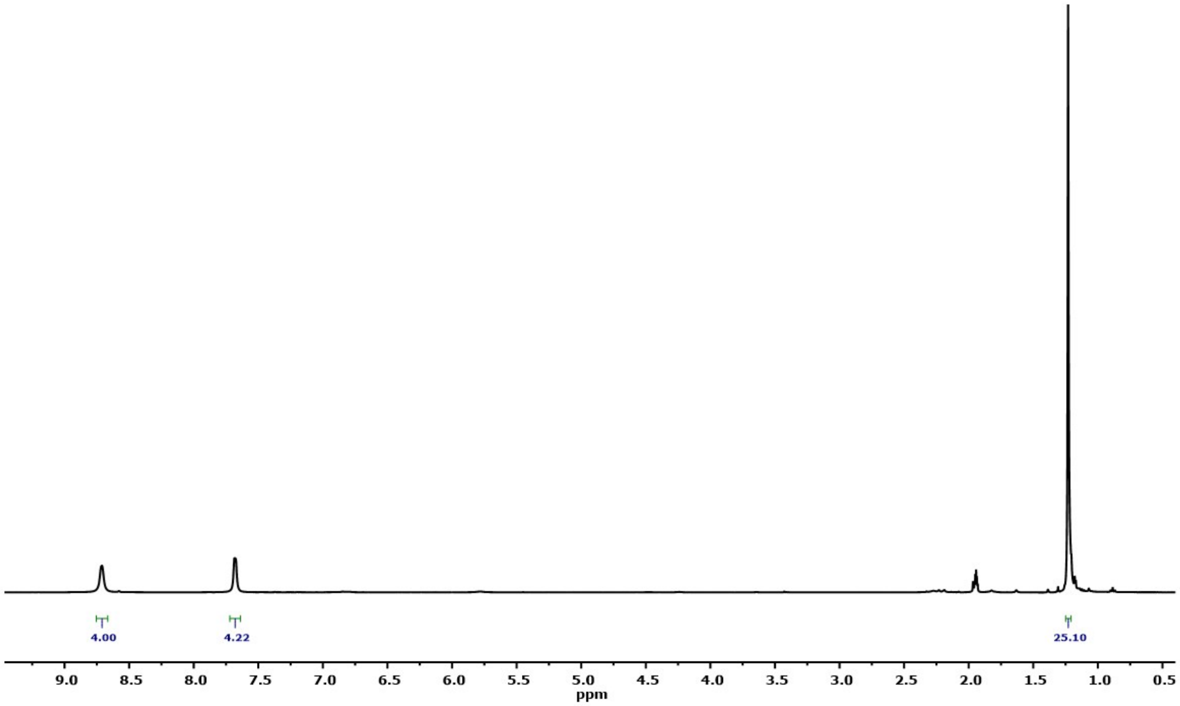

Figure S20. Reaction of (DIM)Ni(NO)(ONO) with (Bpin)$_2$Bpy in a 2:3 mole ratio in CD_3CN to form [(DIM)Ni(NO)]$_2$. All starting material has been consumed, with no excess borylating reagent present. All [(DIM)Ni(NO)]$_2$ has crashed out of solution, leaving only 4,4'-bipyridine and boryl ether in the ^{1}H NMR in a 1:1 ratio.

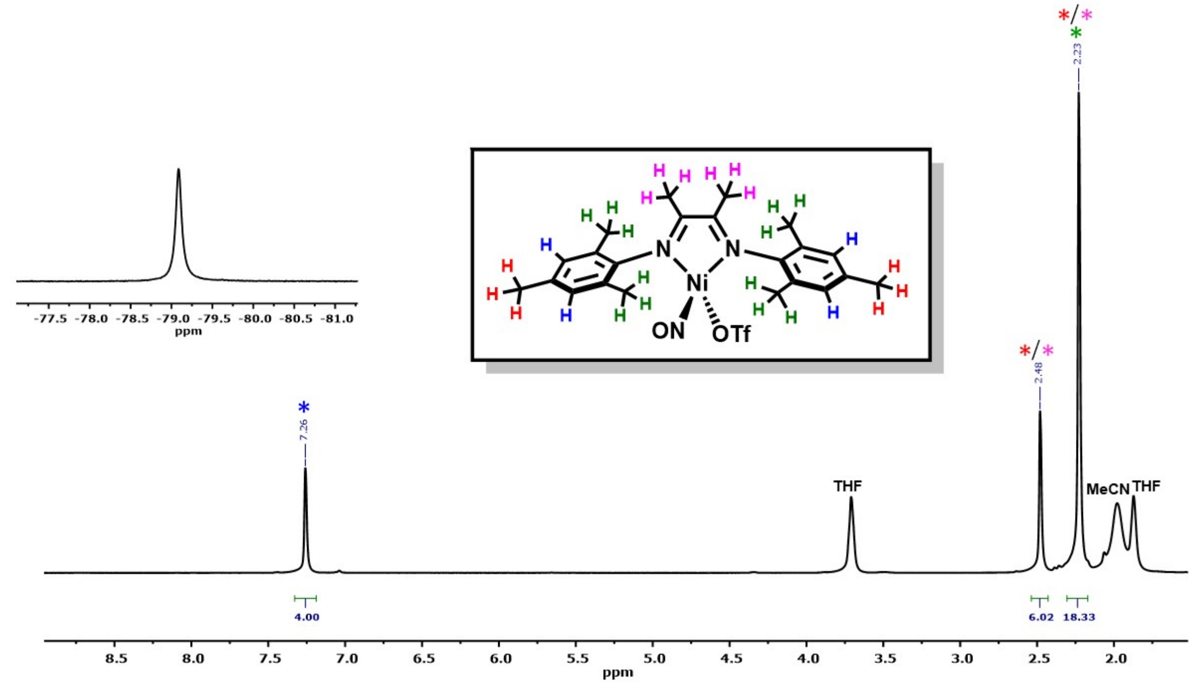

Figure S21. ^{1}H NMR spectrum and ^{19}F NMR spectrum (inset) of (DIM)Ni(NO)(OTf) in CD_3CN

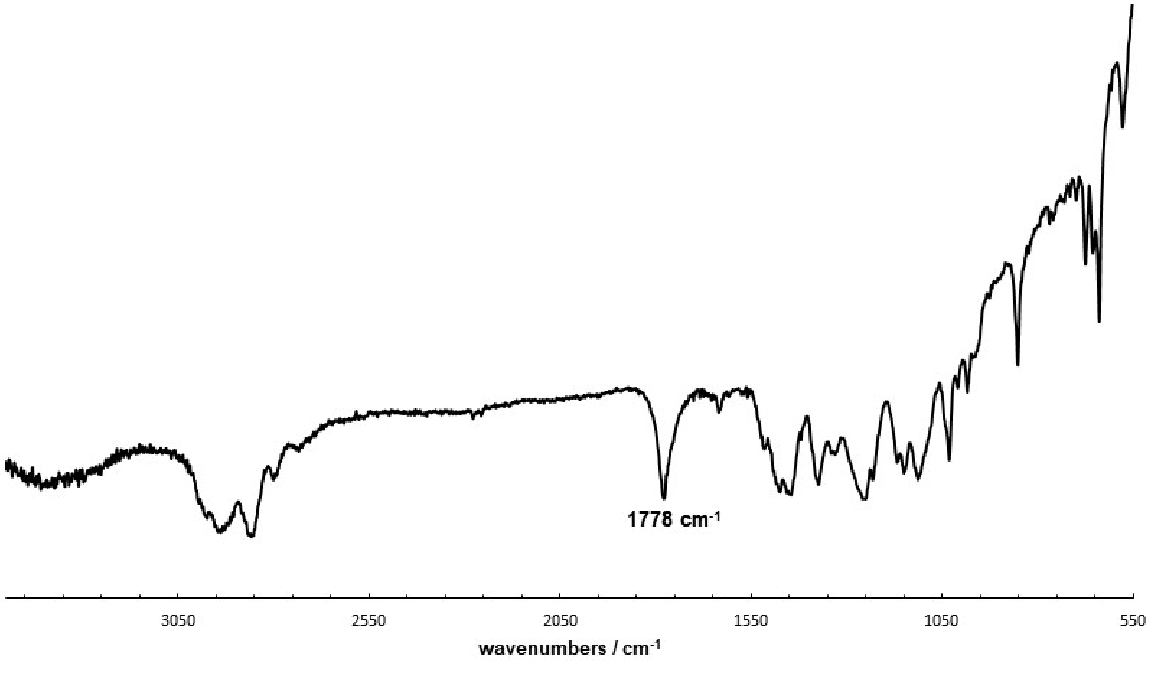

Figure 22. FT-IR spectrum of (DIM)Ni(NO)(OTf)

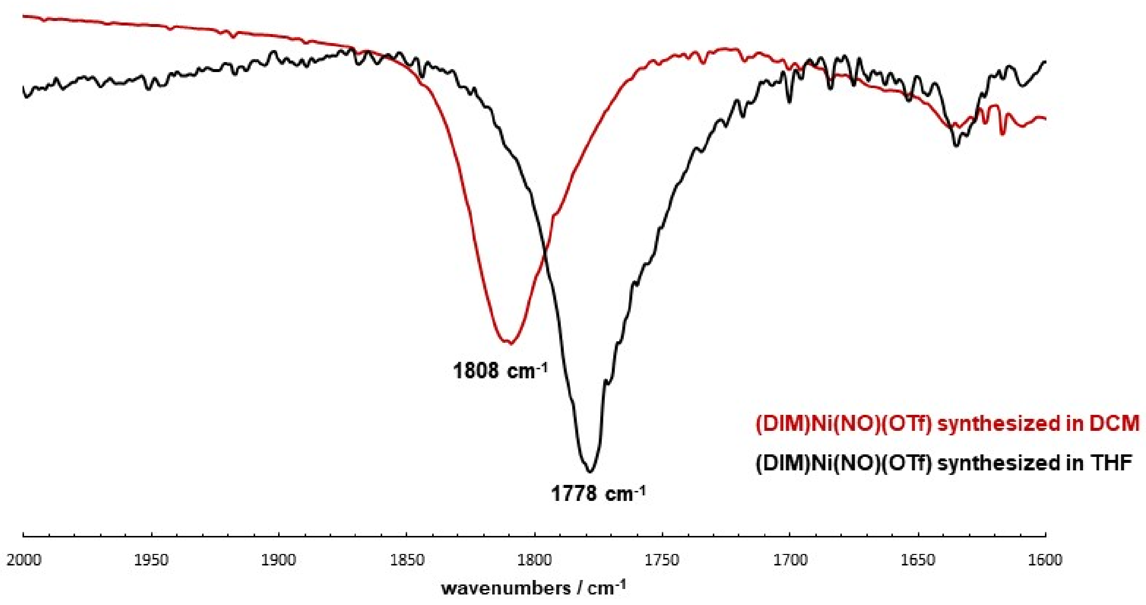

Figure S23. FT-IR spectrum of (DIM)Ni(NO)(OTf) synthesized using THF (black) and using only dichloromethane (red), showing a shift in the nitrosyl stretch when using a non-coordinating solvent.

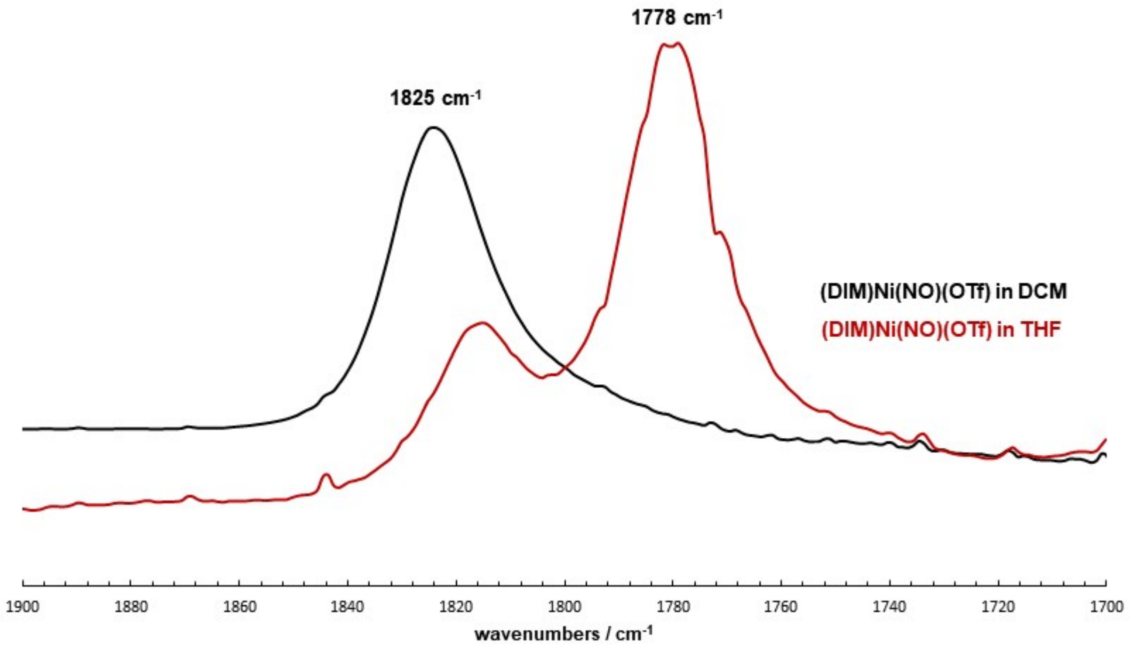

Figure 24. Solution phase FT-IR spectrum of (DIM)Ni(NO)(OTf) synthesized using THF (red) and using only dichloromethane (black), showing a shift in the nitrosyl stretch when using a non-coordinating solvent. The presence of two nitrosyl stretches in THF solvent indicates an equilibrium between THF and triflate coordinated to the nickel center.

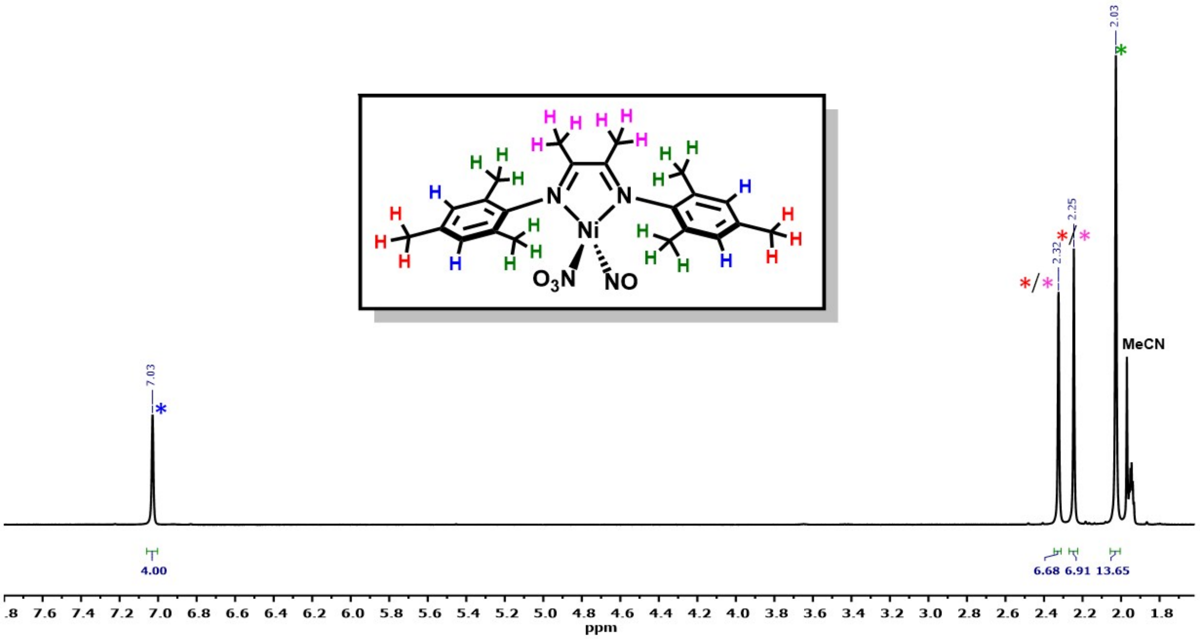

Figure S25. ^{1}H NMR spectrum of (DIM)Ni(NO)(ONO_2) in CD_3CN

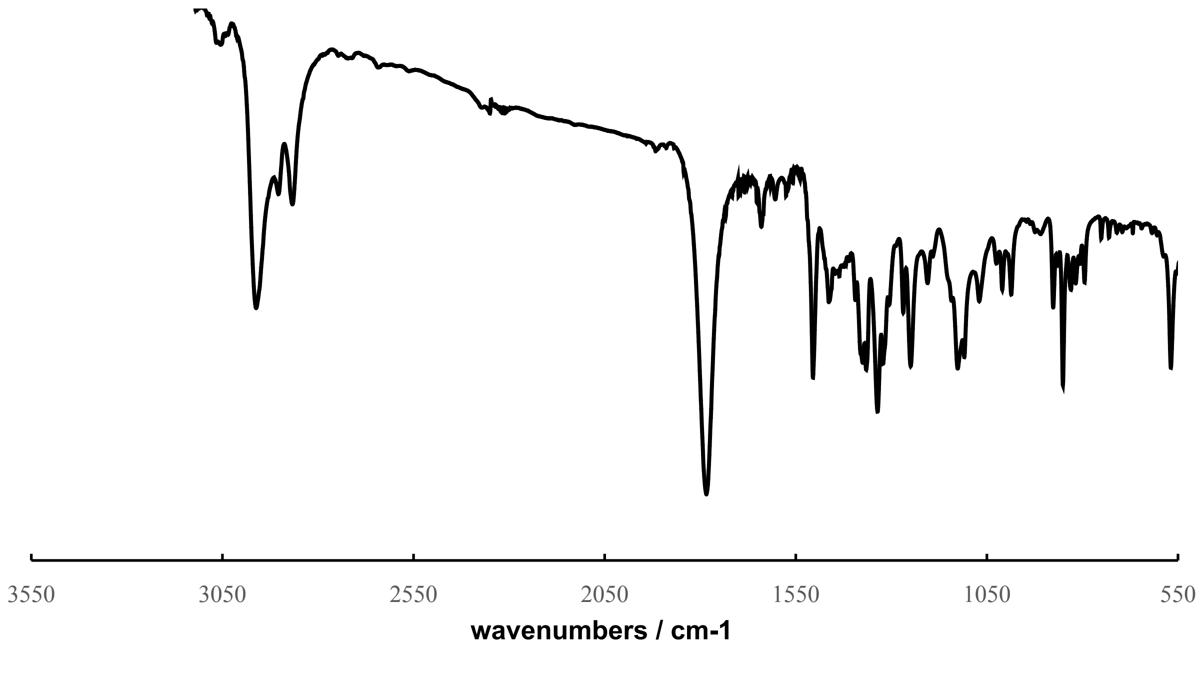

Figure S26. FT-IR spectrum of (DIM)Ni(NO)(ONO_2) (KBr)

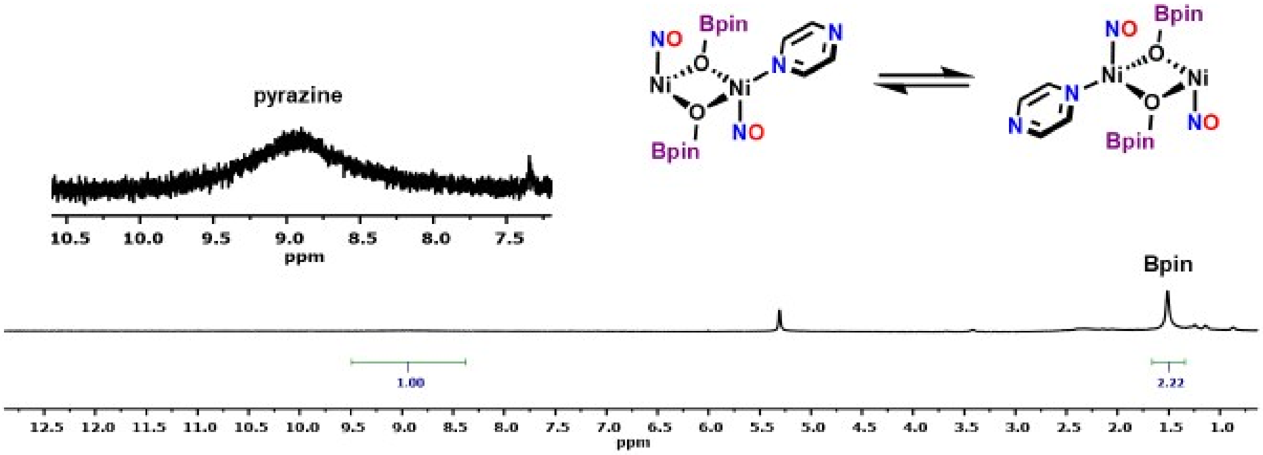

Figure S27: ^{1}H NMR of $(OBPin)_2Ni_2(NO)_2(Pz)$ in CD_2Cl_2. Dynamic exchange of the pyrazine causes broadening of the expected pyrazine resonances. The sample was washed with ether (2 x 3 mL) and pentane (2 x 3 mL) to remove any organic byproducts, and the O(Bpin) chemical shift is different from those of $(Bpin)_2Pz$ and $(Bpin)_2O$ in CD_2Cl_2.

In spite of being polymeric, $Ni_2(NO)_2(OBpin)_2(Pz)$ dissolves in THF, DCM and MeCN. The ^{1}H NMR spectrum in CD_2Cl_2 shows one signal for coordinated pyrazine and one signal for the pinacolate methyls, but the pyrazine signal is unusually broad and suggests some dynamic character. The simplest explanation is that the dissolved species is the repeat unit, $Ni_2(NO)_2(OBpin)_2(\kappa^1\text{-}Pz)$, and that some dynamic process averages the two inequivalent pyrazine chemical shifts for monodentate pyrazine coordinated to only one end of the $Ni_2(NO)_2(OBpin)_2$ unit, which therefore has one three-coordinate and one four-coordinate nickel. We suggest that either a dissociative or an associate process causes exchange of free and coordinated pyrazine nitrogen, and this process occurs at an intermediate exchange rate under our measurement conditions.

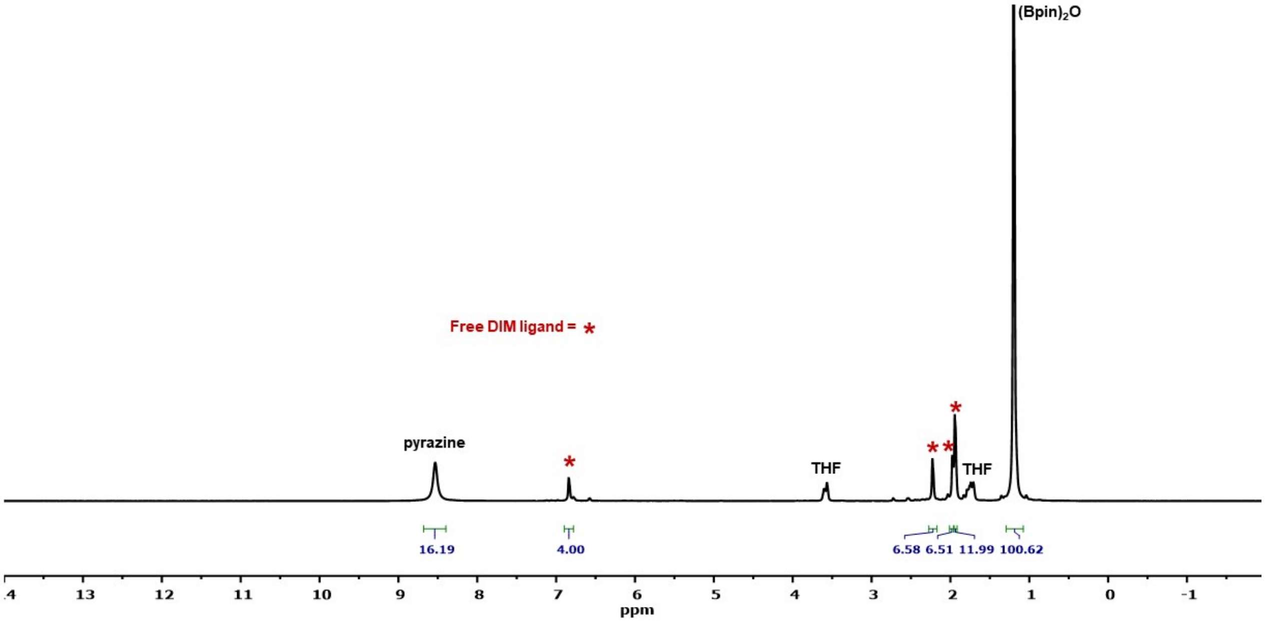

Figure S28. ^{1}H NMR spectrum of the reaction between $(DIM)Ni(NO_2)_2$ and $(Bpin)_2Pz$ executed in d_8-THF at 80 °C , showing the formation of pyrazine, boryl ether, and free DIM.

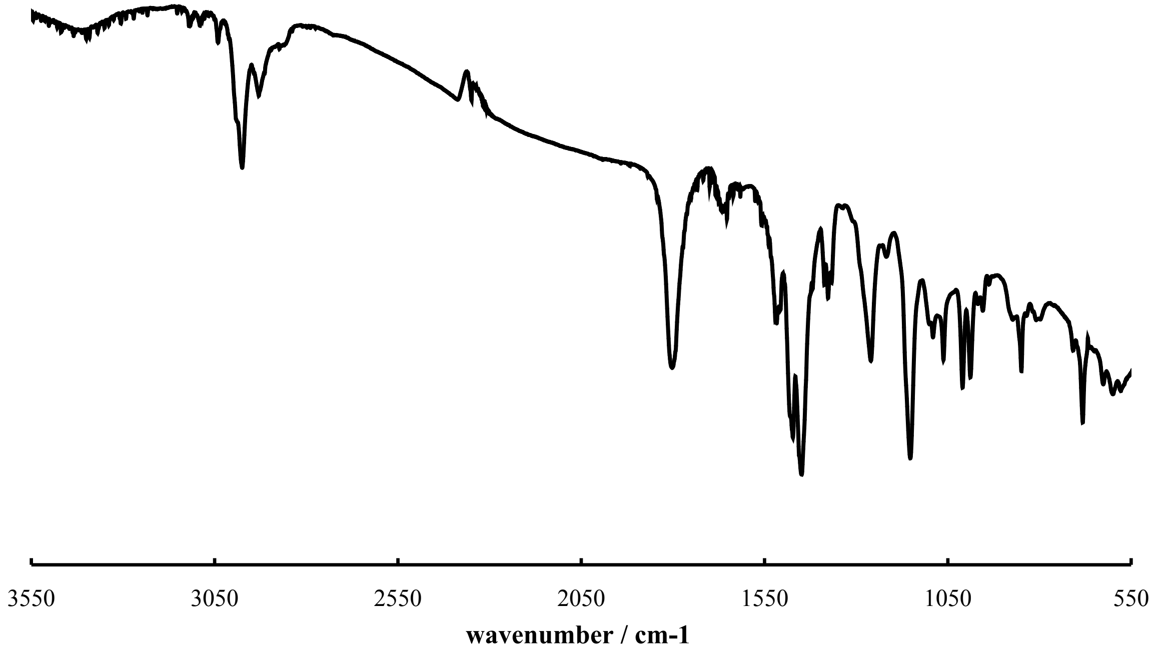

Figure S29: FT-IR spectrum of $(OBPin)_2Ni_2(NO)_2(Pz)$

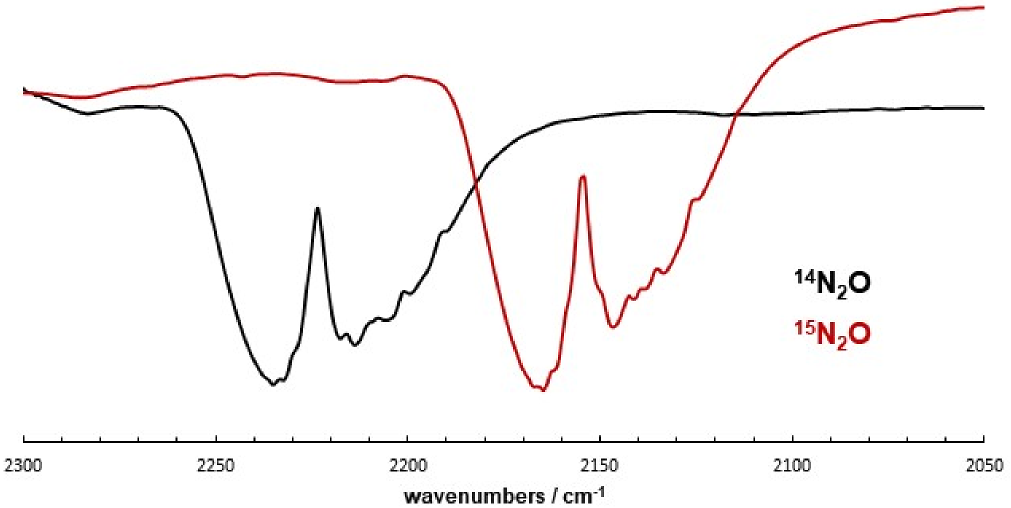

Figure S30. Gas phase FT-IR spectrum of $^{14}N_2O$ formed from deoxygenation of (DIM)Ni(NO)(ONO) (black trace) and $^{15}N_2O$ formed from deoxygenation of (DIM)Ni(^{15}NO)(O^{15}NO) (red trace)

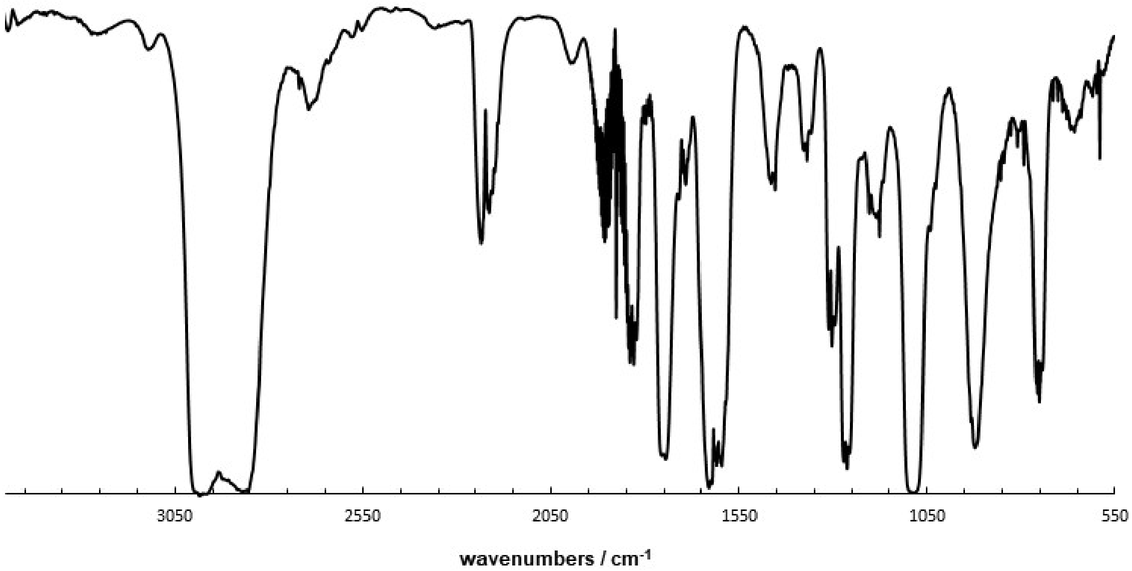

Figure S31. Gas phase FT-IR spectrum of the headspace of the reaction between $[(DIM)Ni(NO)]_2$ and excess NO gas as a THF slurry, showing the diagnostic N_2O stretch at 2224 cm^{-1} as well as excess nitric oxide at 1865 cm^{-1}.

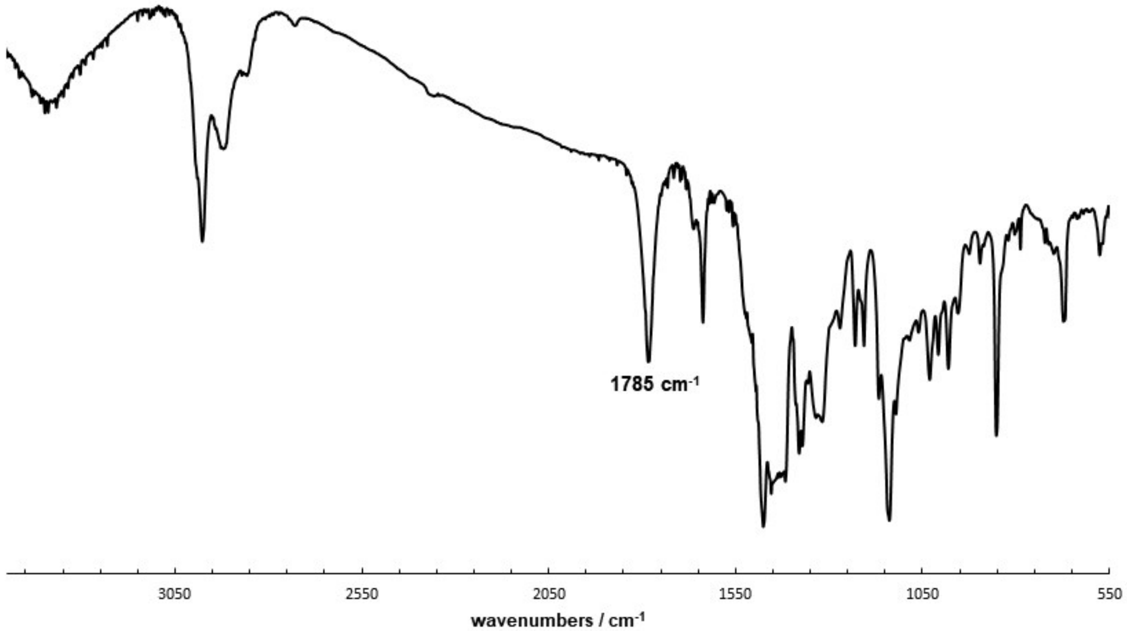

Figure S32. FT-IR spectrum of solid isolated from the reaction of $[(DIM)Ni(NO)]_2$ with excess nitric oxide, showing the diagnostic nitrosyl stretch for (DIM)Ni(NO)(ONO) at 1785 cm^{-1}.

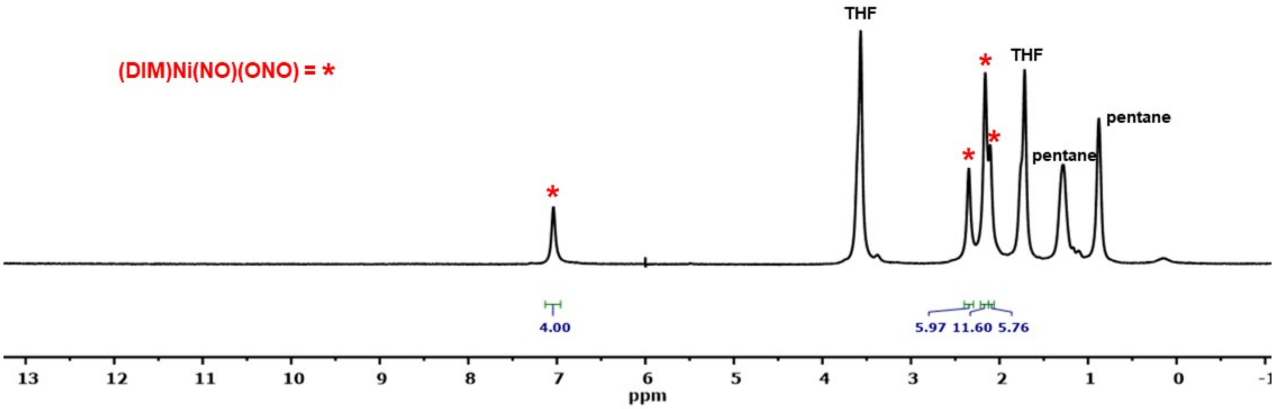

Figure S33. ^{1}H NMR spectrum of solid isolated, after pentane washing, from the reaction of $[(DIM)Ni(NO)]_2$ with excess nitric oxide, showing the diagnostic ^{1}H NMR chemical shifts for (DIM)Ni(NO)(ONO) in d_8-THF

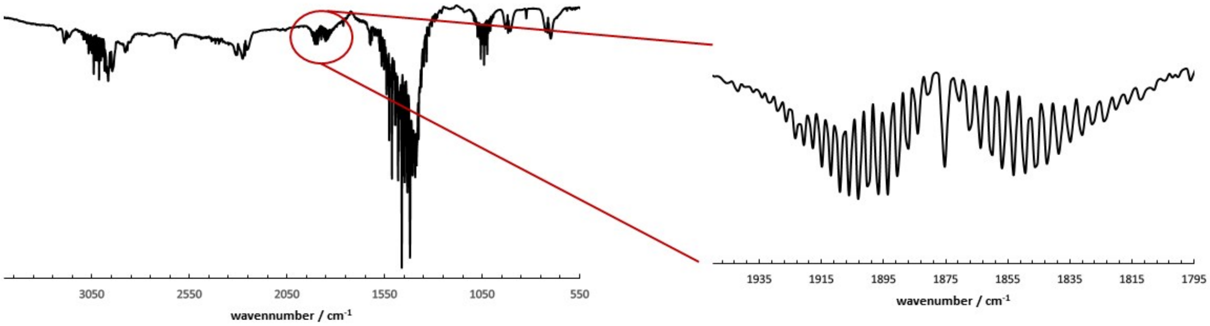

Figure S34. Gas phase FT-IR spectrum of the volatiles after the reaction between (DIM)Ni(NO)(ONO) and $AgNO_2$, showing the formation of NO gas. Other signals in the spectrum correspond to acetonitrile.

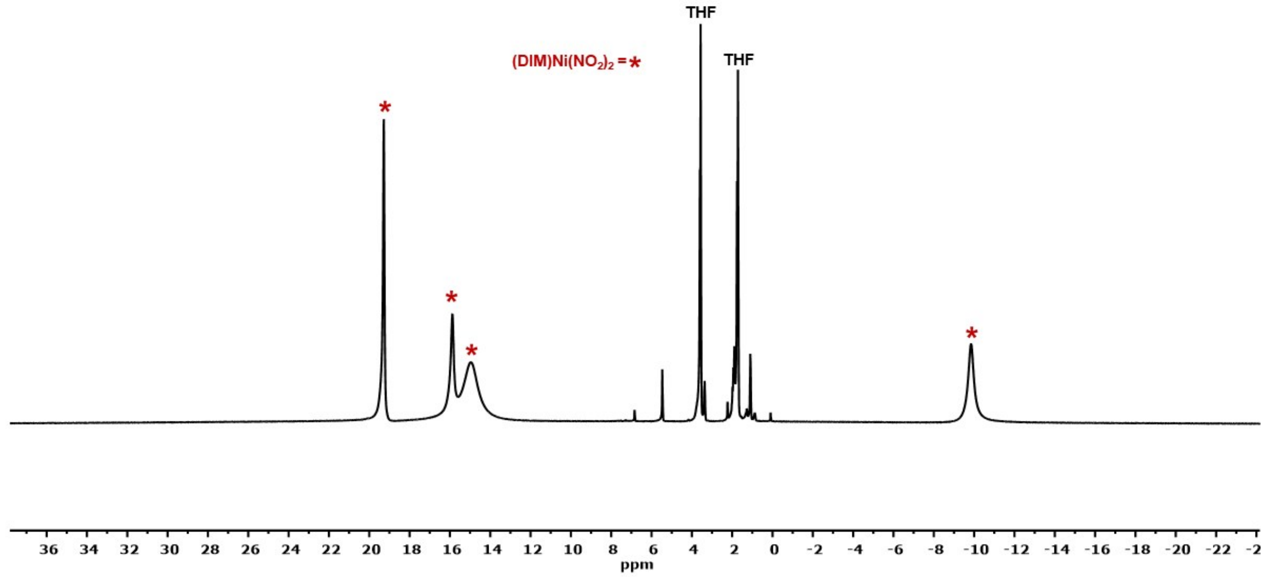

Figure S35. ¹H NMR spectrum in d_8-THF of the reaction between (DIM)Ni(NO)(ONO) and $AgNO_2$, showing the formation of (DIM)Ni$(NO_2)_2$

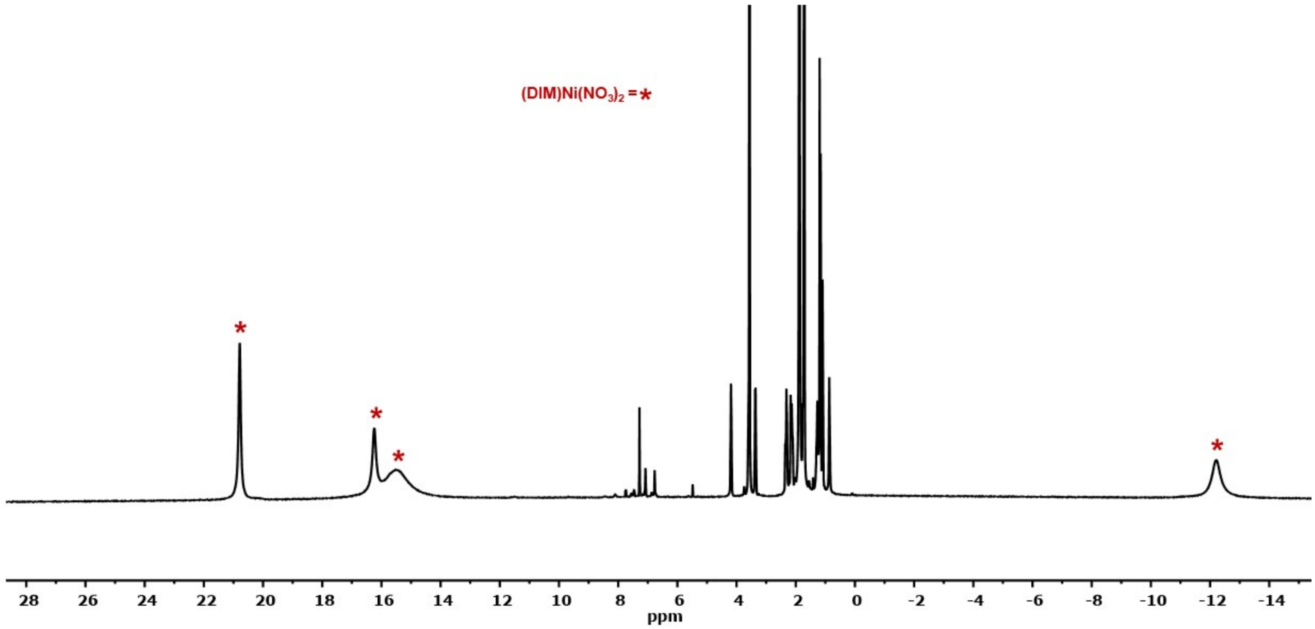

Figure S36. ¹H NMR spectrum in CD_3CN of the reaction between [(DIM)Ni(NO)]$_2$ and excess $AgNO_3$, showing the formation of (DIM)Ni$(NO_3)_2$

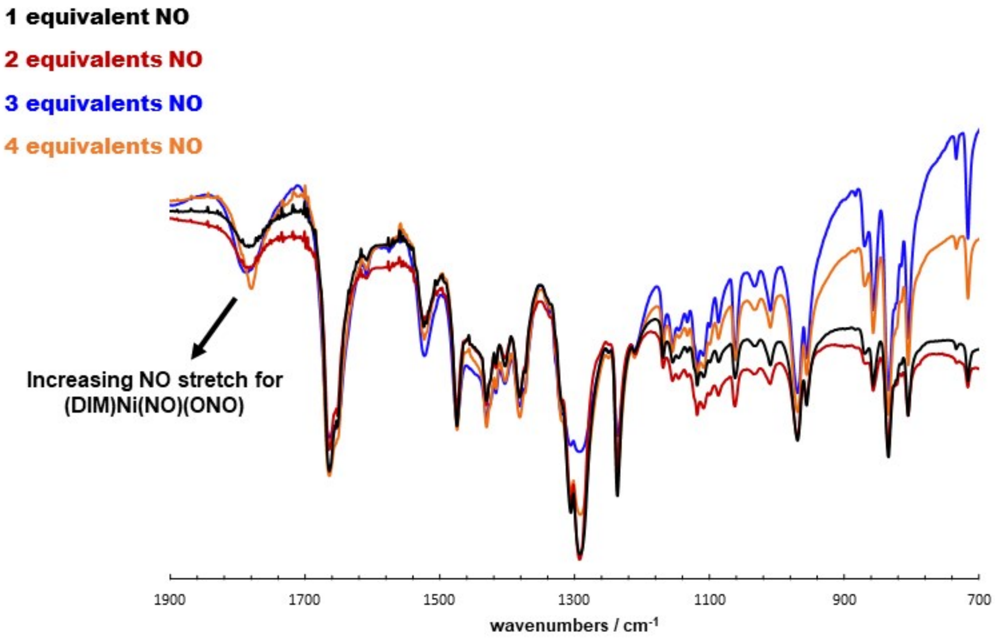

Figure S37. FT-IR spectra of solid-state reaction between $[(DIM)Ni(NO)]_2$ and nitric oxide (KBr press)

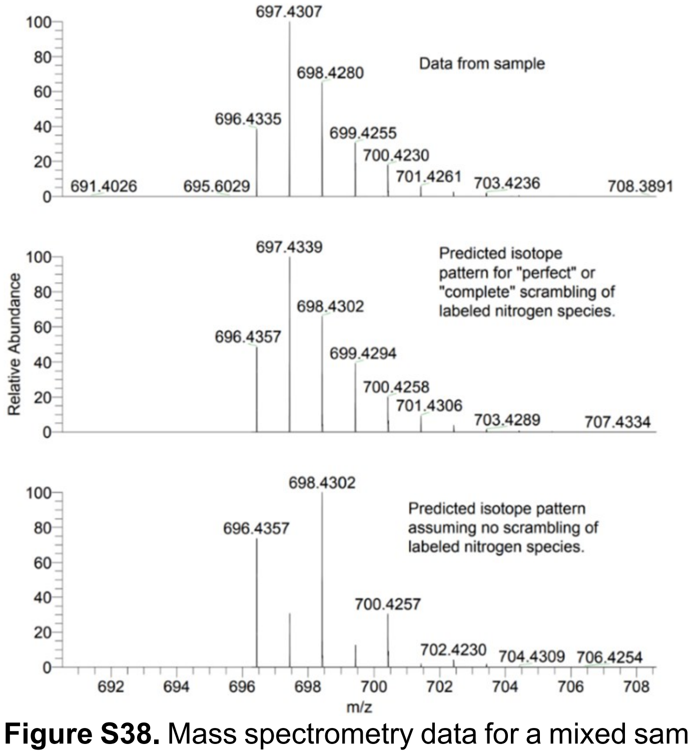

Figure S38. Mass spectrometry data for a mixed sample in a 1:1 mole ratio of (DIM)Ni(NO)(ONO) and (DIM)Ni(^{15}NO)(O^{15}NO) in THF. The m/z observed is for the intact molecular complex plus tetrabutylammonium as the cation. The isotopic pattern from the experimental spectrum matches with the predicted spectrum for isotopic scrambling, yielding a 1:1:1:1 ratio of (DIM)Ni(^{15}NO)(O^{15}NO), (DIM)Ni(^{14}NO)(O^{15}NO), (DIM)Ni(^{15}NO)(O^{14}NO), and (DIM)Ni(^{14}NO)(O^{14}NO).

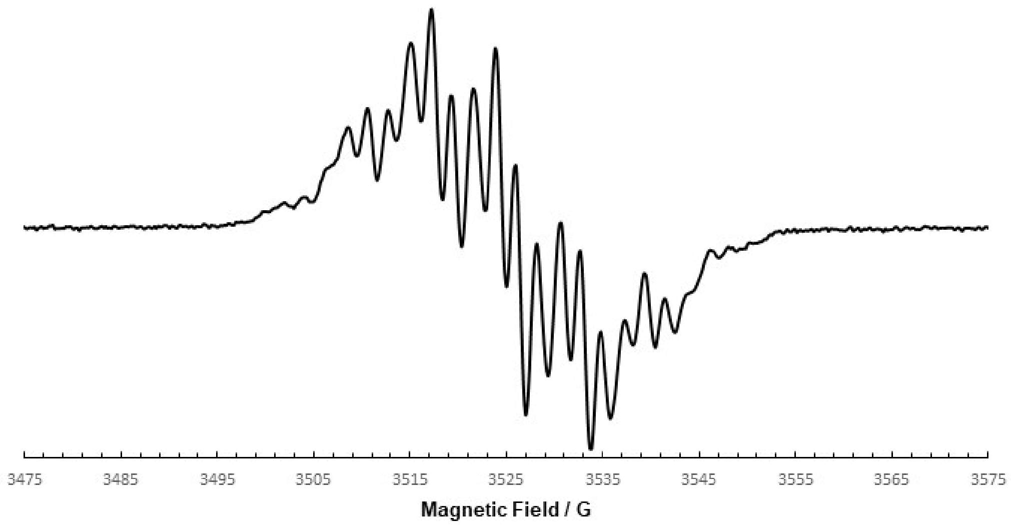

Figure S39. X-band EPR spectrum of the reaction between (DIM)Ni(NO)(ONO) and Cp_2*Co in THF, showing the same spectrum that was collected after OSET from $(Bpin)_2$Bpy to (DIM)Ni(NO)(ONO)

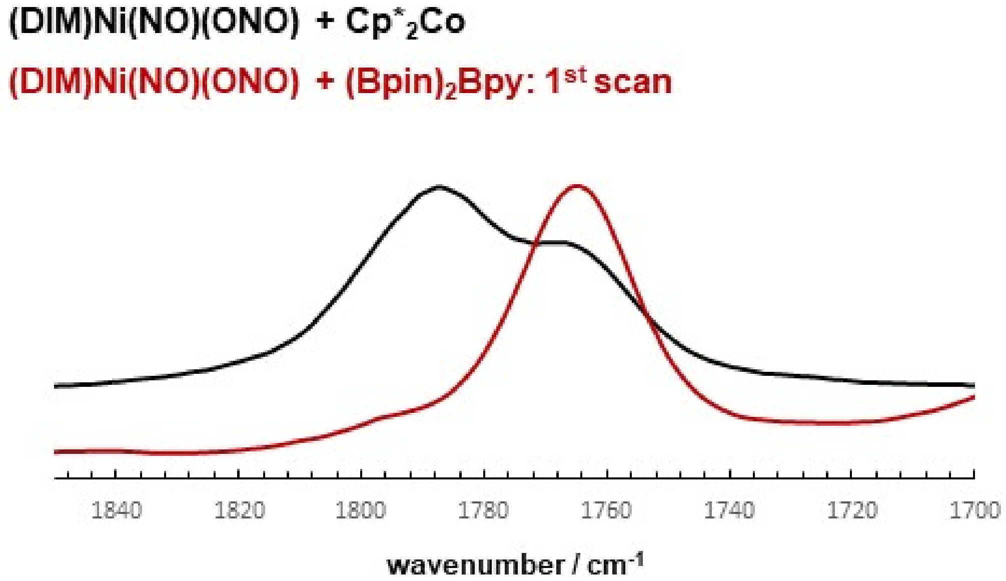

Figure S40. Solution IR in THF of (DIM)Ni(NO)(ONO) after addition of Cp_2*Co (black trace) overlaid with the first solution IR taken immediately after addition of $(Bpin)_2$Bpy to (DIM)Ni(NO)(ONO), indicating that the same species is formed from each OSET event.

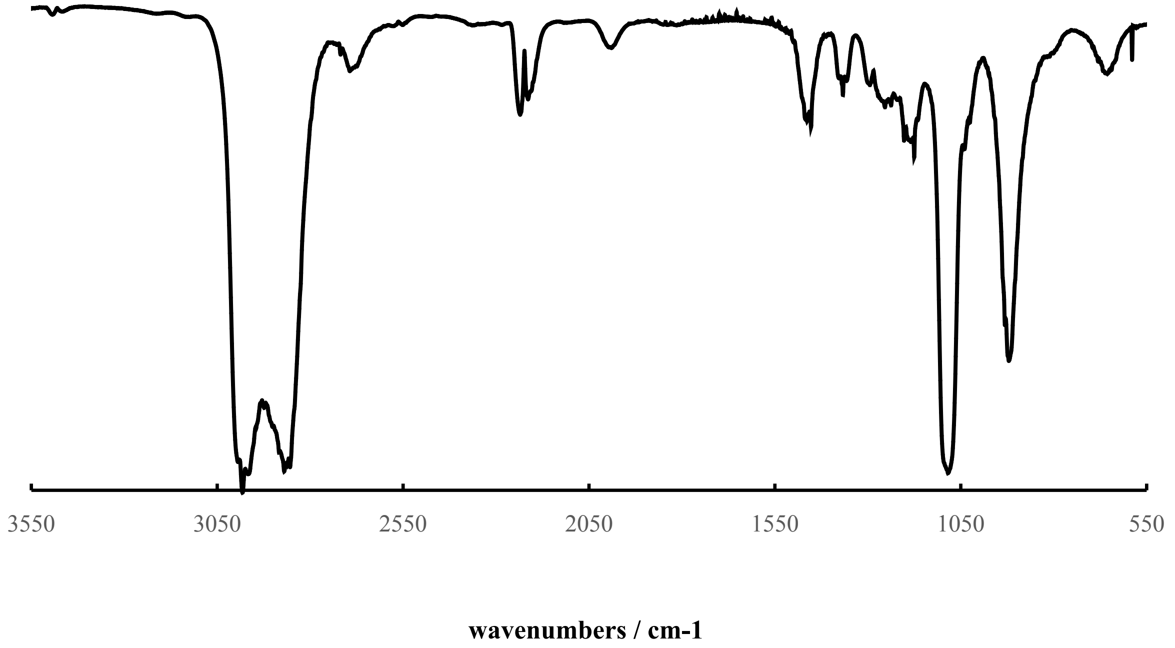

Figure S41. Vacuum transfer of the volatiles from the reaction of (DIM)Ni(NO_2)$_2$ with (Bpin)$_2$Pz in a 2:3 mole ratio at 80 °C. Characteristic N_2O stretch is at 2224 cm^{-1}, other significant peaks are due to THF that was transferred from the headspace of the reaction.

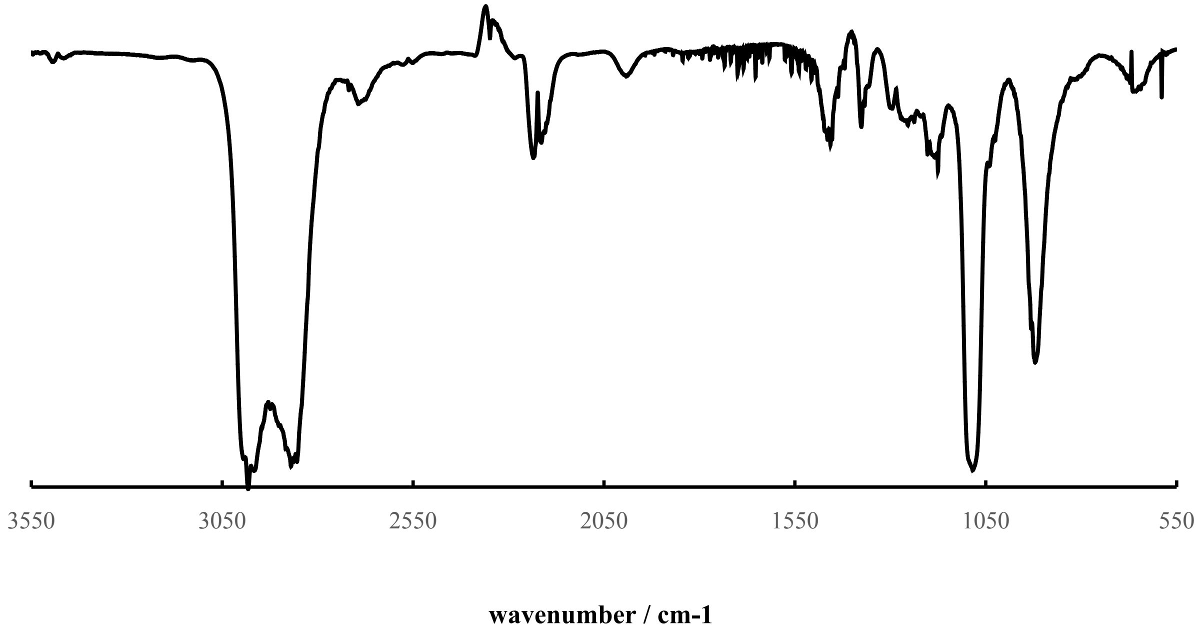

Figure S42. Vacuum transfer of the volatiles from the reaction of (DIM)Ni(NO_2)$_2$ with (Bpin)$_2$bpy in a 2:3 mole ratio at 80 °C. Characteristic N_2O stretch is at 2224 cm^{-1}, other significant peaks are due to THF that was transferred from the headspace of the reaction.

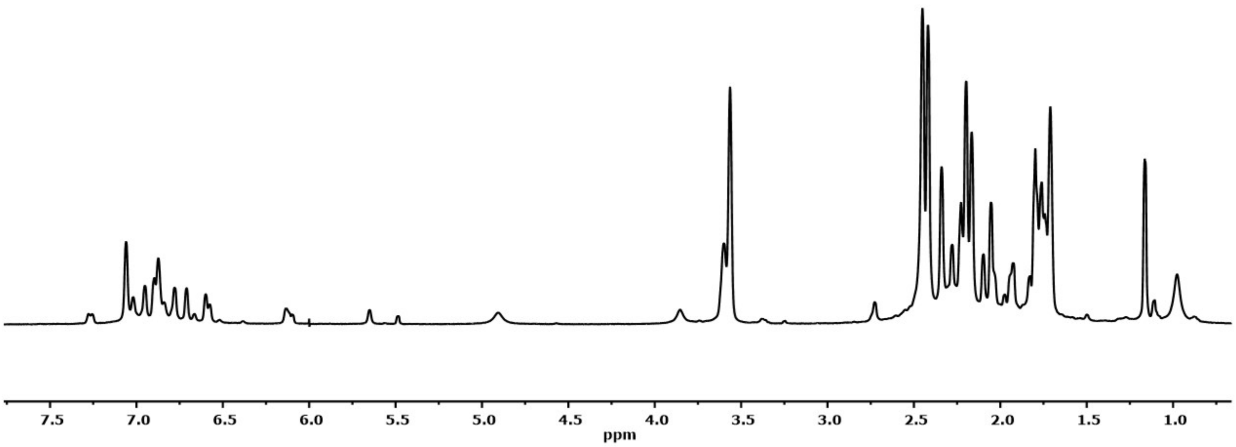

Figure S43. ^{1}H NMR of (DIM)Ni(NO)(ONO) after heating at 80 °C in d_8-THF for 3 hours

XPS

XPS data is summarized in Figure S44. Comparison of the bis-nitrate and bis-nitrite complexes in Fig S44b shows a 4 eV shift to lower binding energy, consistent with pentavalent nitrogen being more difficult to ionize than trivalent nitrogen. The corresponding peaks at 399.8 in both the bis-nitrate and bis-nitrite can be attributed to the di-imine ligand nitrogens. When the bis-nitrate and bis-nitrite complexes are compared to the dimer, [(DIM)Ni(NO)]$_2$ (Fig S44a-c), there is a 0.7 eV shift of the Ni 2p binding energy to a lower binding energy, consistent with a more reduced nickel metal center in the dimer. Furthermore, the nitrosyl nitrogen is resolved from the ligand based nitrogens as a shoulder on the larger di-imine nitrogen peak. The binding energy for the nitrosyl nitrogen matches the binding energy for the nitrite nitrogens in the bis-nitrite complex, supporting a cationic nitrosyl with a nitrogen oxidation state of +3. The O1s peak of [(DIM)Ni(NO)]$_2$ is shifted by 0.57 eV to lower binding energy in comparison to the nitrate and nitrite starting materials. This can be rationalized by a large degree of back donation into the linear nitrosyl π^* orbital, which is supported by the low NO stretching frequency.

Fig S44 d-f compares the dimer to (DIM)Ni(NO)(NO_2), and the Ni2p region does not appear to be shifted; however, there is a diminishing of the satellite peaks. The N1s region for (DIM)Ni(NO)(NO_2) is asymmetric and broad, which suggests multiple types of nitrogen are present, despite lower resolution than in the other samples. The shoulder tailing towards higher binding energies indicates oxidized nitrogen, either arising from nitrite or the linear nitrosyl ligand. The O1s region for (DIM)Ni(NO)(NO_2) deviates greatly from symmetric, indicating incipient resolution of multiple types of oxygen. The relative intensity of the shoulder at higher binding energies is 1:2.

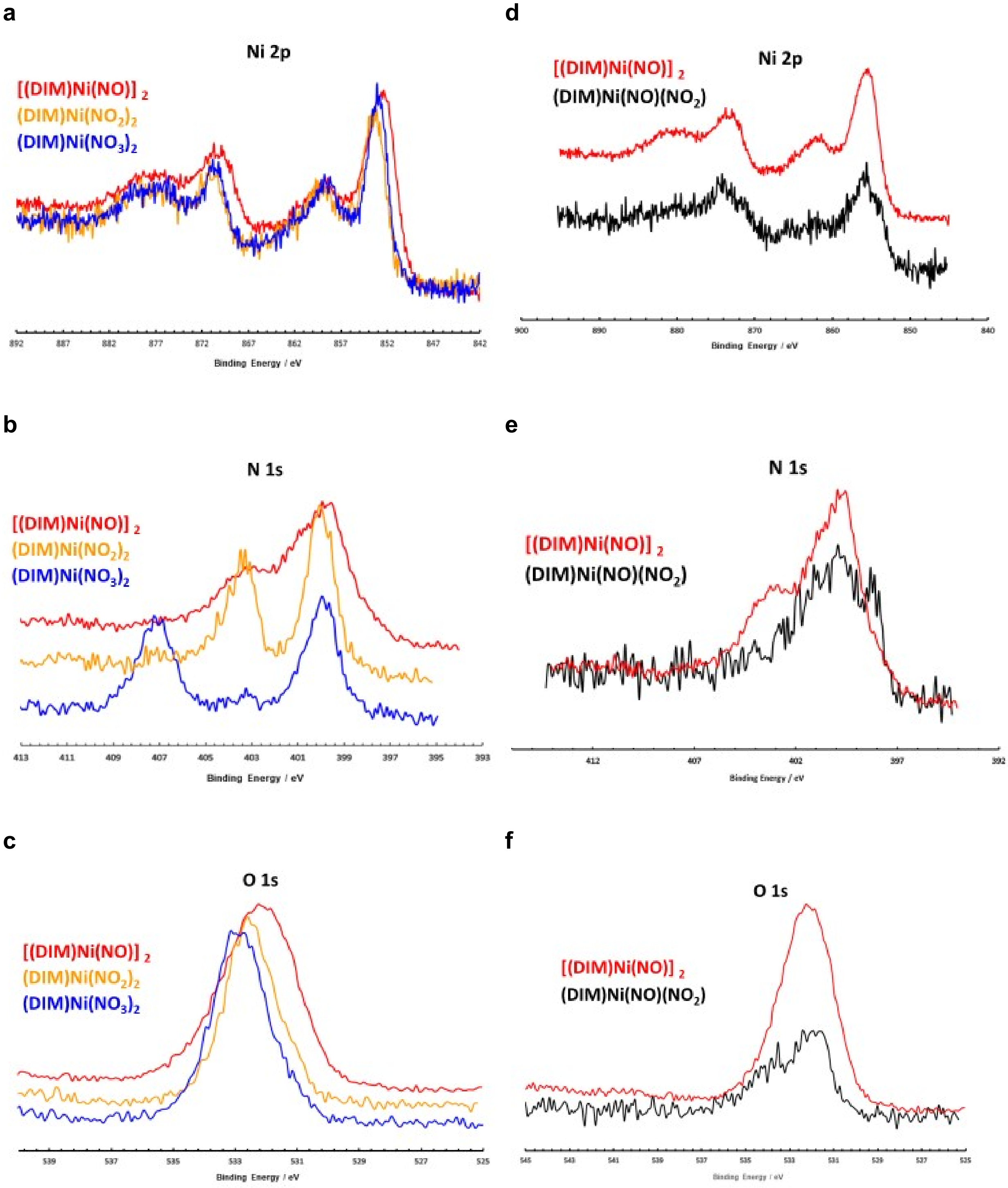

Figure S44. High resolution XPS data a) Ni2p region for $[(DIM)Ni(NO)]_2$ (red), $(DIM)Ni(NO_2)_2$ (orange), and $(DIM)Ni(NO_3)_2$ (blue) then b) N1s region and c) O1s region for same species. d) Ni2p region for $[(DIM)Ni(NO)]_2$ (red) and $(DIM)Ni(NO)(NO_2)$ (black) then e) N1s region and f)

O1s region for same species.

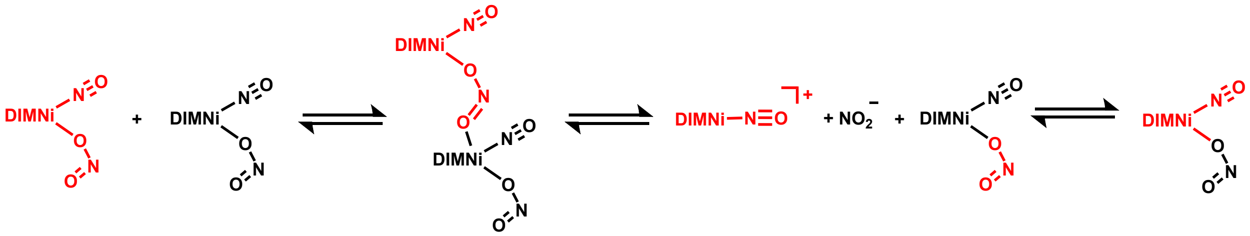

Scheme S1. Proposed nitrito exchange with (DIM)Ni(NO)(ONO). A nitrito ligand can act as a nucleophile towards an adjacent (DIM)Ni(NO)(ONO) molecule, encouraging nitrite loss and transferring the nitrito ligand. Based on our ^{15}N NMR data, this process is more facile in a coordinating solvent, suggesting that solvent coordination could also encourage nitrite loss.

Table S1. Bond lengths within the DIM NCCN backbone for compounds reported here.

Complex	C-C bond (Å)	C-N bonds (Å)
(DIM)Ni(NO)(ONO)	1.498	1.292, 1.283
(DIM)Ni(NO_2)$_2$	1.515	1.302, 1.291
[(DIM)Ni(NO)]$_2$	1.436	1.328, 1.311
(DIM)Ni(NO_3)$_2$	1.502	1.285, 1.283

Typical bond lengths for C=N in an unreduced α-diimine ligand: 1.27-1.28
Typical bond lengths for C-C single bond in unreduced α-diimine ligand: 1.49-1.51
Typical bond lengths for C=N in a singly reduced α-diimine ligand: 1.32-1.35
Typical bond lengths for C-N in a doubly reduced α-diimine ligand: 1.40-1.42
Typical bond lengths for C-C single bond in a doubly reduced α-diimine ligand: 1.35-1.40

Typical bond lengths presented above[14] are in line with increased electron density in the α-diimine ligand for the [(DIM)Ni(NO)]$_2$ dimer compared to the other nickel complexes reported in the paper. All complexes other than the dimer have C-C and C=N bond lengths consistent with a neutral, unreduced ligand.

Crystallographic data.

Data for (DIM)Ni(NO_3)$_2$ (MSC #19120) CCDC 2083107

General details

A light green crystal (plate, approximate dimensions 0.31 × 0.19 × 0.09 mm^3) was placed onto the tip of a MiTeGen pin and mounted on a Bruker Venture D8 diffractometer equipped with a PhotonIII detector at 100.0 K.

Data collection

The data collection was carried out using Mo Kα radiation (λ = 0.71073 Å, graphite monochromator) with a frame time of 0.75 seconds and a detector distance of 50 mm. A collection strategy was calculated and complete data to a resolution of 0.75 Å with a redundancy of 3 were collected. A total of 816 frames were collected. The total exposure time was 0.17 hours. The frames were integrated with the Bruker SAINT[4] software package using a narrow-frame algorithm. The integration of the data using a monoclinic unit cell yielded a total of 54924 reflections to a

maximum θ angle of 28.32° (0.75 Å resolution), of which 12914 were independent (average redundancy 4.253, completeness = 99.4%, R_{int} = 17.84%, R_{sig} = 18.52%) and 7264 (56.25%) were greater than $2\sigma(F^2)$. The final cell constants of a = 14.981(2) Å, b = 13.932(2) Å, c = 25.176(3) Å, β = 97.521(4)°, volume = 5209.4(12) $Å^3$, are based upon the refinement of the XYZ-centroids of reflections above 20 σ(I). Data were corrected for absorption effects using the Multi-Scan method (SADABS).[5] The calculated minimum and maximum transmission coefficients (based on crystal size) are 0.7640 and 0.9220. Please refer to Table S2 for additional crystal and refinement information.

Structure solution and refinement

The space group P 1 21/c 1 was determined based on intensity statistics and systematic absences. The structure was solved using the SHELX suite of programs[6] and refined using full-matrix least-squares on F^2 within the OLEX2 suite.[7] An intrinsic phasing solution was calculated, which provided most non-hydrogen atoms from the E-map. Full-matrix least squares / difference Fourier cycles were performed, which located the remaining non-hydrogen atoms. All non-hydrogen atoms were refined with anisotropic displacement parameters. The hydrogen atoms were placed in ideal positions and refined as riding atoms with relative isotropic displacement parameters. The final full matrix least squares refinement converged to R1 = 0.0942 and wR2 = 0.2307 (F^2, all data). The goodness-of-fit was 1.028. On the basis of the final model, the calculated density was 1.419 g/cm^3 and F(000), 2322 e^-.

Table S2: Crystal data and structure refinement for $(DIM)Ni(NO_3)_2$

Empirical formula	C45.25 H58.50 Cl2.50 N8 Ni2 O12	
Formula weight	1112.54	
Crystal color, shape, size	light green plate, 0.31 × 0.19 × 0.09 mm^3	
Temperature	100.0 K	
Wavelength	0.71073 Å	
Crystal system, space group	Monoclinic, P 1 21/c 1	
Unit cell dimensions	a = 14.981(2) Å	α = 90°.
	b = 13.932(2) Å	β = 97.521(4)°.
	c = 25.176(3) Å	γ = 90°.
Volume	5209.4(12) $Å^3$	
Z	4	
Density (calculated)	1.419 g/cm^3	
Absorption coefficient	0.917 mm^{-1}	
F(000)	2322	
**Data collection**		
Diffractometer	Bruker VENTURE D8	
Theta range for data collection	2.231 to 28.319°.	
Index ranges	-19<=h<=19, -13<=k<=18, -33<=l<=33	
Reflections collected	54924	
Independent reflections	12914 [R_{int} = 0.1784]	
Observed Reflections	7264	
Completeness to theta = 25.242°	99.8 %	
**Solution and Refinement**		
Absorption correction	Semi-empirical from equivalents	
Max. and min. transmission	0.7457 and 0.5949	
Solution	Intrinsic methods	
Refinement method	Full-matrix least-squares on F^2	
Weighting scheme	$w = [\sigma^2 Fo^2 + AP^2 + BP]^{-1}$, with	

	P = (Fo^2+ 2 Fc^2)/3, A = 0.0765, B = 0.0000
Data / restraints / parameters	12914 / 630 / 648
Goodness-of-fit on F^2	1.028
Final R indices [I>2σ(I)]	R1 = 0.0942, wR2 = 0.1956
R indices (all data)	R1 = 0.1601, wR2 = 0.2307
Extinction coefficient	n/a
Largest diff. peak and hole	0.998 and -0.994 e.Å^{-3}

Data for (DIM)Ni(NO_2)$_2$ (MSC #19121) CCDC 2083108

General details

Single crystals suitable for X-ray diffraction were grown by vapor diffusion of pentane into a tetrahydrofuran solution. A light brown crystal (plate, approximate dimensions 0.14 × 0.11 × 0.07 mm^3) was placed onto the tip of a MiTeGen pin and mounted on a Bruker Venture D8 diffractometer equipped with a Photon III detector at 100.0 K.

Data collection

The data collection was carried out using Mo Kα radiation (λ = 0.71073 Å, graphite monochromator) with a frame time of 5 s for low angle frames and 40 s for high angle frames. The detector distance was 130 mm. A collection strategy was calculated and complete data to a resolution of 0.75 Å with a redundancy of 7 were collected. A total of 2728 frames were collected. The total exposure time was 21.43 hours. The frames were integrated with the Bruker SAINT[4] software package using a narrow-frame algorithm. The integration of the data using an orthorhombic unit cell yielded a total of 66234 reflections to a maximum θ angle of 25.26° (0.83 Å resolution), of which 8294 were independent (average redundancy 7.986, completeness = 99.0%, Rint = 18.19%, Rsig = 9.59%) and 6583 (79.37%) were greater than 2σ(F2). The final cell constants of a = 13.866(3) Å, b = 14.341(3) Å, c = 23.383(4) Å, volume = 4649.8(15) Å^3, are based upon the refinement of the XYZ-centroids of reflections above 20 σ(I). The calculated minimum and maximum transmission coefficients (based on crystal size) are 0.8880 and 0.9420. Data were corrected for absorption effects using the Multi-Scan method (SADABS).[5] Please refer to Table S3 for additional crystal and refinement information.

Structure solution and refinement

The space group Pca2$_1$ was determined based on intensity statistics and systematic absences. The structure was solved using the SHELX suite of programs[6] and refined using full-matrix least-squares on F^2 within the OLEX2 suite.[7] An intrinsic phasing solution was calculated, which provided most non-hydrogen atoms from the E-map. Full-matrix least squares / difference Fourier cycles were performed, which located the remaining non-hydrogen atoms. All non-hydrogen atoms were refined with anisotropic displacement parameters. The hydrogen atoms were placed in ideal positions and refined as riding atoms with relative isotropic displacement parameters. The final full matrix least squares refinement converged to R1 = 0.0620 and wR2 = 0.1196 (F^2, all data). The goodness-of-fit was 1.051. On the basis of the final model, the calculated density was 1.346 g/cm^3 and F(000), 1984 e^-.

Table S3: Crystal data and structure refinement for (DIM)Ni(NO_2)$_2$

Empirical formula	C44 H56 N8 Ni2 O8
Formula weight	942.38
Crystal color, shape, size	light brown plate, 0.14 × 0.11 × 0.07 mm^3
Temperature	100.0 K
Wavelength	0.71073 Å
Crystal system, space group	Orthorhombic, Pca2$_1$

Unit cell dimensions	a = 13.866(3) Å	α = 90°.
	b = 14.341(3) Å	β = 90°.
	c = 23.383(4) Å	γ = 90°.
Volume	4649.5(15) $Å^3$	
Z	4	
Density (calculated)	1.346 g/cm^3	
Absorption coefficient	0.869 mm^{-1}	
F(000)	1984	
**Data collection**		
Diffractometer	Bruker VENTURE D8	
Theta range for data collection	2.221 to 25.257°.	
Index ranges	-16<=h<=15, -17<=k<=17, -27<=l<=27	
Reflections collected	66234	
Independent reflections	8294 [Rint = 0.1819]	
Observed Reflections	6583	
Completeness to theta = 25.242°	99.2 %	
**Solution and Refinement**		
Absorption correction	Semi-empirical from equivalents	
Max. and min. transmission	0.7452 and 0.5785	
Solution	Intrinsic methods	
Refinement method	Full-matrix least-squares on F^2	
Weighting scheme	$w = [\sigma^2Fo^2 + AP^2 + BP]^{-1}$, with	
	$P = (Fo^2 + 2 Fc^2)/3$, A = 0.0137, B = 9.2433	
Data / restraints / parameters	8294 / 1 / 576	
Goodness-of-fit on F^2	1.051	
Final R indices [I>2σ(I)]	R1 = 0.0620, wR2 = 0.1114	
R indices (all data)	R1 = 0.0858, wR2 = 0.1196	
Absolute structure parameter	-0.005(16)	
Extinction coefficient	n/a	
Largest diff. peak and hole	0.642 and -0.693 $eÅ^{-3}$	
**Twin Details**		
Type, twin law	pseudomerohedral, -1 0 0, 0 1 0, 0 0 -1	
Twin element, domain ratio	180° rotation in reciprocal space about 0 1 0, 53.3 : 47.3	

Data for (DIM)Ni(NO)(ONO) (MSC #20009) CCDC 2083111

General details

A green crystal (approximate dimensions 0.25 × 0.12 × 0.11 mm^3) was placed onto the tip of a 0.05 mm diameter glass capillary and mounted on a Bruker Venture D8 diffractometer equipped with a PhotonIII detector at 100(2) K.

Data collection

The data collection was carried out using Mo Kα radiation (graphite monochromator) with a frame time of 2 seconds and a detector distance of 4.00 cm. A collection strategy was calculated and complete data to a resolution of 0.71 Å with a redundancy of 3.4 were collected. Five major sections of frames were collected with 0.50° ω and ϕ scans. A total of 1640 frames were collected. The total exposure time was 1.27 hours. The frames were integrated with the Bruker SAINT software package[4] using a narrow-frame algorithm. The integration of the data using a monoclinic unit cell yielded a total of 51978 reflections to a maximum θ angle of 28.32° (0.75 Å resolution), of which 5558 were independent (average redundancy 9.352, completeness = 99.8%, R_{int} = 7.45%, R_{sig} = 4.20%) and 4368 (78.59%) were greater than 2σ(F^2). The final cell constants of a = 11.6061(7) Å, b = 16.8020(11) Å, c = 12.3282(8) Å, β = 111.731(2)°, volume = 2233.2(2) $Å^3$, are based upon the refinement of the XYZ-centroids of 9002 reflections above 20 σ(I) with 6.014° <

2θ < 56.34°. Data were corrected for absorption effects using the Multi-Scan method (SADABS).[5] The ratio of minimum to maximum apparent transmission was 0.900. The calculated minimum and maximum transmission coefficients (based on crystal size) are 0.8070 and 0.9080. Please refer to Table S4 for additional crystal and refinement information.

Structure solution and refinement

The space group $P2_1/n$ was determined based on intensity statistics and systematic absences. The structure was solved and refined using the SHELX suite of programs.[6] An intrinsic-methods solution was calculated, which provided most non-hydrogen atoms from the E-map. Full-matrix least squares / difference Fourier cycles were performed, which located the remaining non-hydrogen atoms. All non-hydrogen atoms were refined with anisotropic displacement parameters. The hydrogen atoms were placed in ideal positions and refined as riding atoms with relative isotropic displacement parameters. The final anisotropic full-matrix least-squares refinement on F^2 with 279 variables converged at R1 = 4.30%, for the observed data and wR2 = 10.85% for all data. The goodness-of-fit was 1.114. The largest peak in the final difference electron density synthesis was 0.460 $e^-/Å^3$ and the largest hole was -0.515 $e^-/Å^3$ with an RMS deviation of 0.088 $e^-/Å^3$. On the basis of the final model, the calculated density was 1.354 g/cm^3 and F(000), 960 e^-

Table S4: Crystal data and structure refinement for $(DIM)Ni(NO_2)(NO)$

Empirical formula	C22 H28 N4 Ni O3	
Formula weight	455.19	
Crystal color, shape, size	green block, 0.25 × 0.12 × 0.11 mm^3	
Temperature	100(2) K	
Wavelength	0.71073 Å	
Crystal system, space group	Monoclinic, $P2_1/n$	
Unit cell dimensions	a = 11.6060(7) Å	α = 90°.
	b = 16.8019(11) Å	β = 111.731(2)°.
	c = 12.3281(8) Å	γ = 90°.
Volume	2233.2(2) $Å^3$	
Z	4	
Density (calculated)	1.354 Mg/m^3	
Absorption coefficient	0.899 mm^{-1}	
F(000)	960	
**Data collection**		
Diffractometer	Venture D8, Bruker	
Source	IμS 3.0, Incoatec	
Theta range for data collection	2.060 to 28.316°.	
Index ranges	-15<=h<=15, -22<=k<=22, -16<=l<=14	
Reflections collected	51982	
Independent reflections	5558 [Rint = 0.0745]	
Observed Reflections	4368	
Completeness to theta = 25.242°	100.0 %	
**Solution and Refinement**		
Absorption correction	Semi-empirical from equivalents	
Max. and min. transmission	0.7457 and 0.6718	
Solution	Intrinsic methods	
Refinement method	Full-matrix least-squares on F^2	
Weighting scheme	$w = [\sigma^2Fo^2 + AP^2 + BP]^{-1}$, with	
	$P = (Fo^2 + 2 Fc^2)/3$, A = 0.0348, B = 3.2020	
Data / restraints / parameters	5558 / 0 / 279	
Goodness-of-fit on F^2	1.114	

Final R indices [I>2σ (I)]	R1 = 0.0430, wR2 = 0.0969
R indices (all data)	R1 = 0.0602, wR2 = 0.1086
Largest diff. peak and hole	0.456 and -0.513 e.Å^{-3}

Data for [(DIM)Ni(NO)]$_2$ (MSC #20016) CCDC 2083110

General details

A teal crystal (approximate dimensions 0.021 × 0.005 × 0.004 mm^3) was placed onto the tip of a MiTeGen loop and mounted on a Bruker Venture D8 diffractometer equipped with a PhotonIII detector at 100(2) K.

Data collection

The data collection was carried out using Mo Kα radiation (graphite monochromator) with a frame time of 40 seconds and a detector distance of 5.00 cm. A collection strategy was calculated and complete data to a resolution of 0.83 Å with a redundancy of 6 were collected. Six major sections of frames were collected with 0.50º ω and ϕ scans. A total of 1844 frames were collected. The total exposure time was 18.54 hours. The frames were integrated with the Bruker SAINT software package[4] using a narrow-frame algorithm. The integration of the data using a triclinic unit cell yielded a total of 12098 reflections to a maximum θ angle of 25.26° (0.83 Å resolution), of which 3478 were independent (average redundancy 3.478, completeness = 97.8%, R_{int} = 20.83%, R_{sig} = 28.27%) and 1648 (47.38%) were greater than 2σ(F^2). The final cell constants of a = 8.631(6) Å, b = 9.896(6) Å, c = 12.698(8) Å, α = 67.393(17)°, β = 84.22(2)°, γ = 78.271(17)°, volume = 980.0(11) Å^3, are based upon the refinement of the XYZ-centroids of 1234 reflections above 20 σ(I) with 4.532° < 2θ < 39.55°. Data were corrected for absorption effects using the Multi-Scan method (SADABS[5]). The ratio of minimum to maximum apparent transmission was 0.552. The calculated minimum and maximum transmission coefficients (based on crystal size) are 0.9790 and 0.9960. Please refer to Table S5 for additional crystal and refinement information.

Structure solution and refinement

The space group P-1 was determined based on intensity statistics and the lack of systematic absences. The structure was solved and refined using the SHELX suite of programs.[6] An intrinsic-methods solution was calculated, which provided most non-hydrogen atoms from the E-map. Full-matrix least squares / difference Fourier cycles were performed, which located the remaining non-hydrogen atoms. All non-hydrogen atoms were refined with anisotropic displacement parameters. The hydrogen atoms were placed in ideal positions and refined as riding atoms with relative isotropic displacement parameters. The final anisotropic full-matrix least-squares refinement on F^2 with 253 variables converged at R1 = 12.38%, for the observed data and wR2 = 34.84% for all data. The goodness-of-fit was 0.968. The largest peak in the final difference electron density synthesis was 2.711 e$^-$/Å^3 and the largest hole was -1.158 e$^-$/Å^3 with an RMS deviation of 0.160 e$^-$/Å^3. On the basis of the final model, the calculated density was 1.387 g/cm^3 and F(000), 434 e$^-$.

Table S5: Crystal data and structure refinement for [(DIM)Ni(NO)]$_2$

Empirical formula	C22 H28 N3 Ni O	
Formula weight	409.18	
Crystal color, shape, size	teal needle, 0.021 × 0.005 × 0.004 mm^3	
Temperature	100(2) K	
Wavelength	0.71073 Å	
Crystal system, space group	Triclinic, P-1	
Unit cell dimensions	a = 8.631(6) Å	α = 67.393(17)°.
	b = 9.896(6) Å	β = 84.22(2)°.

	c = 12.698(8) Å	γ = 78.271(17)°.
Volume	979.9(11) Å^3	
Z	2	
Density (calculated)	1.387 Mg/m^3	
Absorption coefficient	1.007 mm^{-1}	
F(000)	434	

Data collection

Diffractometer	Venture D8, Bruker
Source	IμS 3.0, Incoatec
Detector	Photon III
Theta range for data collection	2.266 to 25.262°.
Index ranges	-10<=h<=10, -11<=k<=11, -15<=l<=15
Reflections collected	12098
Independent reflections	3478 [R$_{int}$ = 0.2083]
Observed Reflections	1648
Completeness to theta = 25.242°	98.0 %

Solution and Refinement

Absorption correction	Semi-empirical from equivalents
Max. and min. transmission	0.7452 and 0.4112
Solution	Intrinsic methods
Refinement method	Full-matrix least-squares on F^2
Weighting scheme	w = [σ^2Fo^2+ AP^2]$^{-1}$, with P = (Fo^2+ 2 Fc^2)/3, A = 0.1533
Data / restraints / parameters	3478 / 211 / 253
Goodness-of-fit on F^2	0.968
Final R indices [I>2σ(I)]	R1 = 0.1238, wR2 = 0.2828
R indices (all data)	R1 = 0.2105, wR2 = 0.3484
Largest diff. peak and hole	2.711 and -1.158 e.Å^{-3}

Data for (DIM)Ni(NO)(OTf) (MSC #21006) CCDC 2083109

General details

A green crystal (plate, approximate dimensions 0.22 × 0.1 × 0.02 mm^3) was placed onto the tip of a MiTeGen pin and mounted on a Bruker Venture D8 diffractometer equipped with a PhotonIII detector at 133.0 K.

Data collection

The data collection was carried out using Mo Mo Kα radiation (λ = 0.71073 Å, graphite monochromator) with a frame time of 5 seconds and a detector distance of 40 mm. A collection strategy was calculated and complete data to a resolution of 0.77 Å with a redundancy of 5.3 were collected. The frames were integrated with the Bruker SAINT[4] software package using a narrow-frame algorithm. The integration of the data using a monoclinic unit cell yielded a total of 92275 reflections to a maximum θ angle of 27.48° (0.77 Å resolution), of which 6025 were independent (average redundancy 15.315, completeness = 100.0%, Rint = 11.85%, Rsig = 4.31%) and 4579 (76.00%) were greater than 2σ(F^2). The final cell constants of a = 11.4858(3) Å, b = 19.4001(6) Å, c = 12.4864(4) Å, β = 108.9590(10)°, volume = 2631.35(14) Å^3, are based upon the refinement of the XYZ-centroids of 9950 reflections above 20 σ(I) with 4.686° < 2θ < 54.14°. Data were corrected for absorption effects using the Multi-Scan method (SADABS).[5] The ratio of minimum to maximum apparent transmission was 0.887. The calculated minimum and maximum transmission coefficients (based on crystal size) are 0.8320 and 0.9830. Please refer to Table S6

for additional crystal and refinement information.

Structure solution and refinement

The space group P 1 21/n 1 was determined based on intensity statistics and systematic absences. The structure was solved using the SHELX suite of programs[6] and refined using full-matrix least-squares on F^2 within the OLEX2 suite.[7] An intrinsic phasing solution was calculated, which provided most non-hydrogen atoms from the E-map. Full-matrix least squares / difference Fourier cycles were performed, which located the remaining non-hydrogen atoms. All non-hydrogen atoms were refined with anisotropic displacement parameters. The hydrogen atoms were placed in ideal positions and refined as riding atoms with relative isotropic displacement parameters. The final full matrix least squares refinement converged to R1 = 0.0471 and wR2 = 0.1069 (F^2, all data). The goodness-of-fit was 1.039. On the basis of the final model, the calculated density was 1.400 g/cm^3 and F(000), 1153 e^-. The structure has disorder, with Ni coordinated to a triflate molecule as the major disorder component (0.97) and to a chlorine anion as the minor disorder component (0.03).

Table S6: Crystal data and structure refinement for (DIM)Ni(NO)(OTf)

Empirical formula	C22.97 H28 Cl0.03 F2.90 N3 Ni O3.90 S0.97	
Formula weight	554.56	
Crystal color, shape, size	green plate, 0.22 × 0.1 × 0.02 mm^3	
Temperature	133.0 K	
Wavelength	0.71073 Å	
Crystal system, space group	Monoclinic, P 1 21/n 1	
Unit cell dimensions	a = 11.4858(3) Å	α = 90°.
	b = 19.4001(6) Å	β = 108.9590(10)°.
	c = 12.4864(4) Å	γ = 90°.
Volume	2631.35(14) Å^3	
Z	4	
Density (calculated)	1.400 g/cm^3	
Absorption coefficient	0.870 mm^{-1}	
F(000)	1153	
**Data collection**		
Diffractometer	Bruker VENTURE D8	
Theta range for data collection	2.019 to 27.485°.	
Index ranges	-14<=h<=14, -25<=k<=25, -16<=l<=16	
Reflections collected	92275	
Independent reflections	6025 [R_{int} = 0.1185]	
Observed Reflections	4579	
Completeness to theta = 25.242°	100.0 %	
**Solution and Refinement**		
Absorption correction	Semi-empirical from equivalents	

Max. and min. transmission	0.7456 and 0.6610
Solution	Intrinsic methods
Refinement method	Full-matrix least-squares on F^2
Weighting scheme	$w = [\sigma^2Fo^2 + AP^2 + BP]^{-1}$, with
	$P = (Fo^2 + 2\ Fc^2)/3$, A = 0.0403, B = 2.8604
Data / restraints / parameters	6025 / 0 / 328
Goodness-of-fit on F^2	1.039
Final R indices [I>2□(I)]	R1 = 0.0471, wR2 = 0.0982
R indices (all data)	R1 = 0.0700, wR2 = 0.1069
Extinction coefficient	n/a
Largest diff. peak and hole	0.532 and -0.407 e.$Å^{-3}$

Data for $(OBPin)_2Ni_2(NO)_2(Pz)$ (MSC #19123) CCDC 2083112

General details
A teal crystal (approximate dimensions 0.32 × 0.08 × 0.07 mm^3) was placed onto the tip of a 0.05 mm diameter glass capillary and mounted on a Bruker Venture D8 diffractometer equipped with a PhotonIII detector at 100(2) K.

Data collection
The data collection was carried out using Mo Kα radiation (graphite monochromator) with a frame time of 17 seconds and a detector distance of 50 mm. A collection strategy was calculated and complete data to a resolution of 0.77 Å with a redundancy of 5.6 were collected. Four major sections of frames were collected with 0.50° ω and ϕ scans. A total of 1018 frames were collected. The total exposure time was 4.81 hours. The frames were integrated with the Bruker SAINT software package[4] using a narrow-frame algorithm. The integration of the data using a monoclinic unit cell yielded a total of 21147 reflections to a maximum θ angle of 27.53° (0.77 Å resolution), of which 3630 were independent (average redundancy 5.826, completeness = 99.6%, R_{int} = 6.87%, R_{sig} = 5.04%) and 2816 (77.58%) were greater than $2\sigma(F^2)$. The final cell constants of a = 12.209(2) Å, b = 7.5518(13) Å, c = 17.864(3) Å, β = 106.243(5)°, volume = 1581.3(5) $Å^3$, are based upon the refinement of the XYZ-centroids of 3676 reflections above 20 σ(I) with 4.725° < 2θ < 49.42°. Data were corrected for absorption effects using the Multi-Scan method (SADABS).[5] The ratio of minimum to maximum apparent transmission was 0.843. The calculated minimum and maximum transmission coefficients (based on crystal size) are 0.6910 and 0.9180. Please refer to Table S7 for additional crystal and refinement information.

Structure solution and refinement
The space group $P2_1/n$ was determined based on intensity statistics and systematic absences. The structure was solved and refined using the SHELX suite of programs.[6] An intrinsic-methods solution was calculated, which provided most non-hydrogen atoms from the E-map. Full-matrix least squares / difference Fourier cycles were performed, which located the remaining non-hydrogen atoms. All non-hydrogen atoms were refined with anisotropic displacement parameters. The hydrogen atoms were placed in ideal positions and refined as riding atoms with relative isotropic displacement parameters. The final anisotropic full-matrix least-squares refinement on F^2 with 194 variables converged at R1 = 4.67%, for the observed data and wR2 = 11.08% for all data. The goodness-of-fit was 1.033. The largest peak in the final difference electron density synthesis was 1.351 $e^-/Å^3$ and the largest hole was -0.663 $e^-/Å^3$ with an RMS deviation of 0.088 $e^-/Å^3$. On the basis of the final model, the calculated density was 1.444 g/cm^3 and F(000), 724 e^-

Table S7: Crystal data and structure refinement for (OBPin)Ni(NO)(Pz)

Empirical formula	C24 H44 B2 N4 Ni2 O10	
Formula weight	687.67	
Crystal color, shape, size	teal needle, 0.32 × 0.08 × 0.07 mm^3	
Temperature	100(2) K	
Wavelength	0.71073 Å	
Crystal system, space group	Monoclinic, $P2_1/n$	
Unit cell dimensions	a = 12.209(2) Å	α = 90°.
	b = 7.5518(13) Å	β = 106.243(5)°.
	c = 17.864(3) Å	γ = 90°.
Volume	1581.4(5) $Å^3$	
Z	2	
Density (calculated)	1.444 Mg/m^3	
Absorption coefficient	1.247 mm^{-1}	
F(000)	724	
Data collection		
Diffractometer	Venture D8, Bruker	
Detector	PhotonIII	
Theta range for data collection	2.363 to 27.532°.	
Index ranges	-15<=h<=14, -9<=k<=9, -18<=l<=23	
Reflections collected	21147	
Independent reflections	3630 [R_{int} = 0.0687]	
Observed Reflections	2816	
Completeness to theta = 25.242°	99.9 %	
Solution and Refinement		
Absorption correction	Semi-empirical from equivalents	
Max. and min. transmission	0.7456 and 0.6282	
Solution	Intrinsic methods	
Refinement method	Full-matrix least-squares on F^2	
Weighting scheme	$w = [\sigma^2Fo^2 + AP^2 + BP]^{-1}$, with	
	$P = (Fo^2 + 2 Fc^2)/3$, A = 0.0502, B = 1.1965	
Data / restraints / parameters	3630 / 0 / 194	
Goodness-of-fit on F^2	1.033	
Final R indices [$I>2\sigma(I)$]	R1 = 0.0467, wR2 = 0.1018	
R indices (all data)	R1 = 0.0669, wR2 = 0.1108	
Largest diff. peak and hole	1.351 and -0.663 e.$Å^{-3}$	

Goodness-of-fit = $[\Sigma[w(F_o^2 - F_c^2)^2]/N_{observns} - N_{params})]^{1/2}$, all data.

$R1 = \Sigma(|F_o| - |F_c|) / \Sigma |F_o|$. $wR2 = [\Sigma[w(F_o^2 - F_c^2)^2] / \Sigma [w(F_o^2)^2]]^{1/2}$.

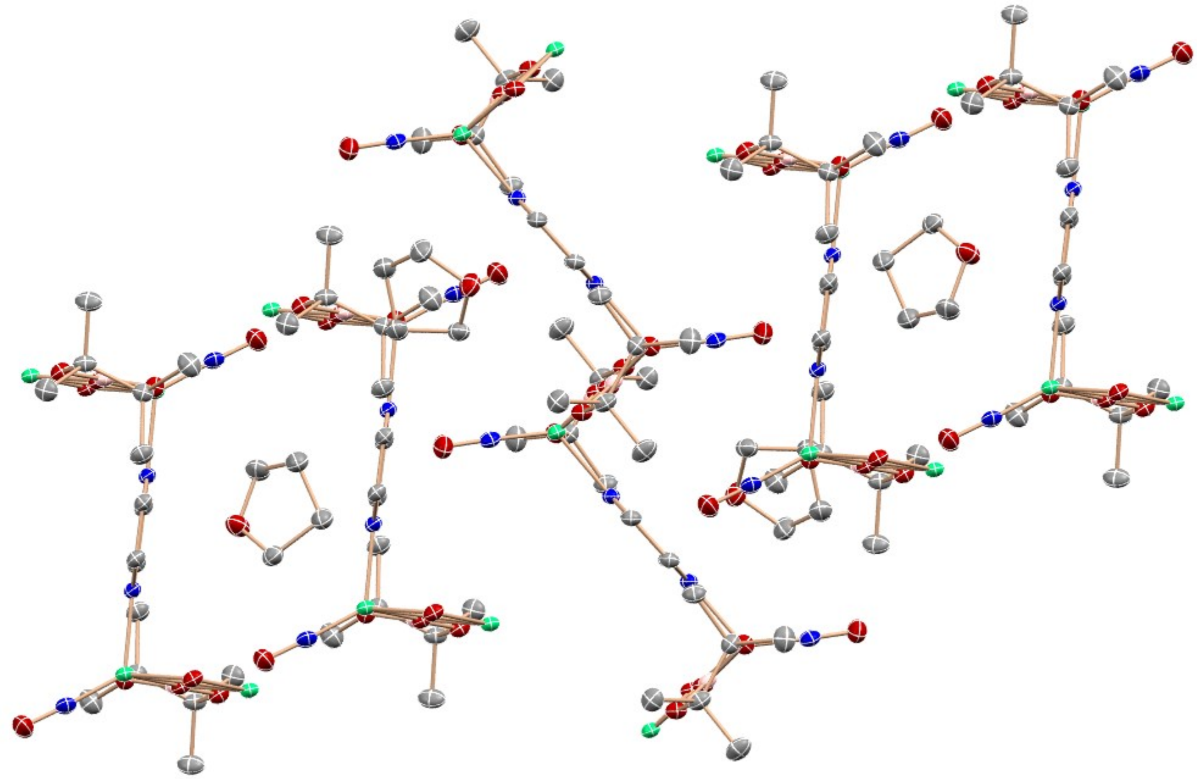

Figure S45: Crystal packing of $(OBPin)_2Ni_2(NO)_2(Pz)$ viewed along the a axis

Data for $(OBPin)_2Ni_2(NO)_2(Bpy)$ (MSC #19181) CCDC 2083113

General details

A blue crystal (approximate dimensions 0.23 × 0.18 × 0.02 mm^3) was placed onto the tip of a 0.05 mm diameter glass capillary and mounted on a Bruker Venture D8 diffractometer equipped with a PhotonIII detector at 123(2) K.

Data collection

The data collection was carried out using Mo Kα radiation (graphite monochromator) with a frame time of 3 seconds and a detector distance of 4.00 cm. A collection strategy was calculated and complete data to a resolution of 0.71 Å with a redundancy of 11 were collected. Eight major sections of frames were collected with 0.50° ω and ϕ scans. A total of 1823 frames were collected. The total exposure time was 0.93 hours. The frames were integrated with the Bruker SAINT software package[4] using a narrow-frame algorithm. The integration of the data using a monoclinic unit cell yielded a total of 48522 reflections to a maximum θ angle of 30.06° (0.71 Å resolution), of which 4277 were independent (average redundancy 11.345, completeness = 99.9%, R_{int} = 7.82%, R_{sig} = 3.75%) and 3269 (76.43%) were greater than $2\sigma(F^2)$. The final cell constants of a = 10.2092(5) Å, b = 11.9426(6) Å, c = 11.9808(5) Å, β = 91.560(2)°, volume = 1460.21(12) $Å^3$, are based upon the refinement of the XYZ-centroids of 9722 reflections above 20 σ(I) with 4.816° < 2θ < 57.28°. Data were corrected for absorption effects using the Multi-Scan method (SADABS).[5] The ratio of minimum to maximum apparent transmission was 0.880. The calculated minimum and maximum transmission coefficients (based on crystal size) are 0.7480 and 0.9740. Please refer to Table S8 for additional crystal and refinement information.

Structure solution and refinement

The space group $P2_1/c$ was determined based on intensity statistics and systematic absences. The structure was solved and refined using the SHELX suite of programs.[6] An intrinsic-methods solution was calculated, which provided most non-hydrogen atoms from the E-map. Full-matrix least squares / difference Fourier cycles were performed, which located the remaining non-hydrogen atoms. All non-hydrogen atoms were refined with anisotropic displacement parameters. The hydrogen atoms were placed in ideal positions and refined as riding atoms with relative isotropic displacement parameters. The final anisotropic full-matrix least-squares refinement on F^2 with 176 variables converged at R1 = 3.98%, for the observed data and wR2 = 9.63% for all data. The goodness-of-fit was 1.040. The largest peak in the final difference electron density synthesis was 0.650 $e^-/Å^3$ and the largest hole was -0.726 $e^-/Å^3$ with an RMS deviation of 0.095 $e^-/Å^3$. On the basis of the final model, the calculated density was 1.409 g/cm^3 and F(000), 644 e^-

Table S8: Crystal data and structure refinement for $(OBPin)_2Ni_2(NO)_2(Bpy)$

Empirical formula	C11 H16 B N2 Ni O4	
Formula weight	309.78	
Crystal color, shape, size	blue plate, 0.23 × 0.18 × 0.02 mm^3	
Temperature	123(2) K	
Wavelength	0.71073 Å	
Crystal system, space group	Monoclinic, $P2_1/c$	
Unit cell dimensions	a = 10.2092(5) Å	α = 90°.
	b = 11.9426(6) Å	β = 91.560(2)°.
	c = 11.9808(5) Å	γ = 90°.
Volume	1460.21(12) $Å^3$	
Z	4	
Density (calculated)	1.409 Mg/m^3	
Absorption coefficient	1.337 mm^{-1}	
F(000)	644	
**Data collection**		
Diffractometer	Venture D8, Bruker	
Detector	Photon III	
Theta range for data collection	2.408 to 30.060°.	
Index ranges	-14<=h<=14, -16<=k<=16, -16<=l<=16	
Reflections collected	48522	
Independent reflections	4277 [Rint = 0.0782]	
Observed Reflections	3269	
Completeness to theta = 25.242°	99.9 %	
**Solution and Refinement**		
Absorption correction	Semi-empirical from equivalents	
Max. and min. transmission	0.7460 and 0.6568	
Solution	Intrinsic methods	
Refinement method	Full-matrix least-squares on F^2	
Weighting scheme	$w = [\sigma^2Fo^2 + AP^2 + BP]^{-1}$, with	
	$P = (Fo^2 + 2 Fc^2)/3$, A = 0.0334, B = 2.0731	
Data / restraints / parameters	4277 / 0 / 176	
Goodness-of-fit on F^2	1.040	
Final R indices [$I>2\sigma(I)$]	R1 = 0.0398, wR2 = 0.0858	
R indices (all data)	R1 = 0.0596, wR2 = 0.0963	
Largest diff. peak and hole	0.650 and -0.726 e.$Å^{-3}$	

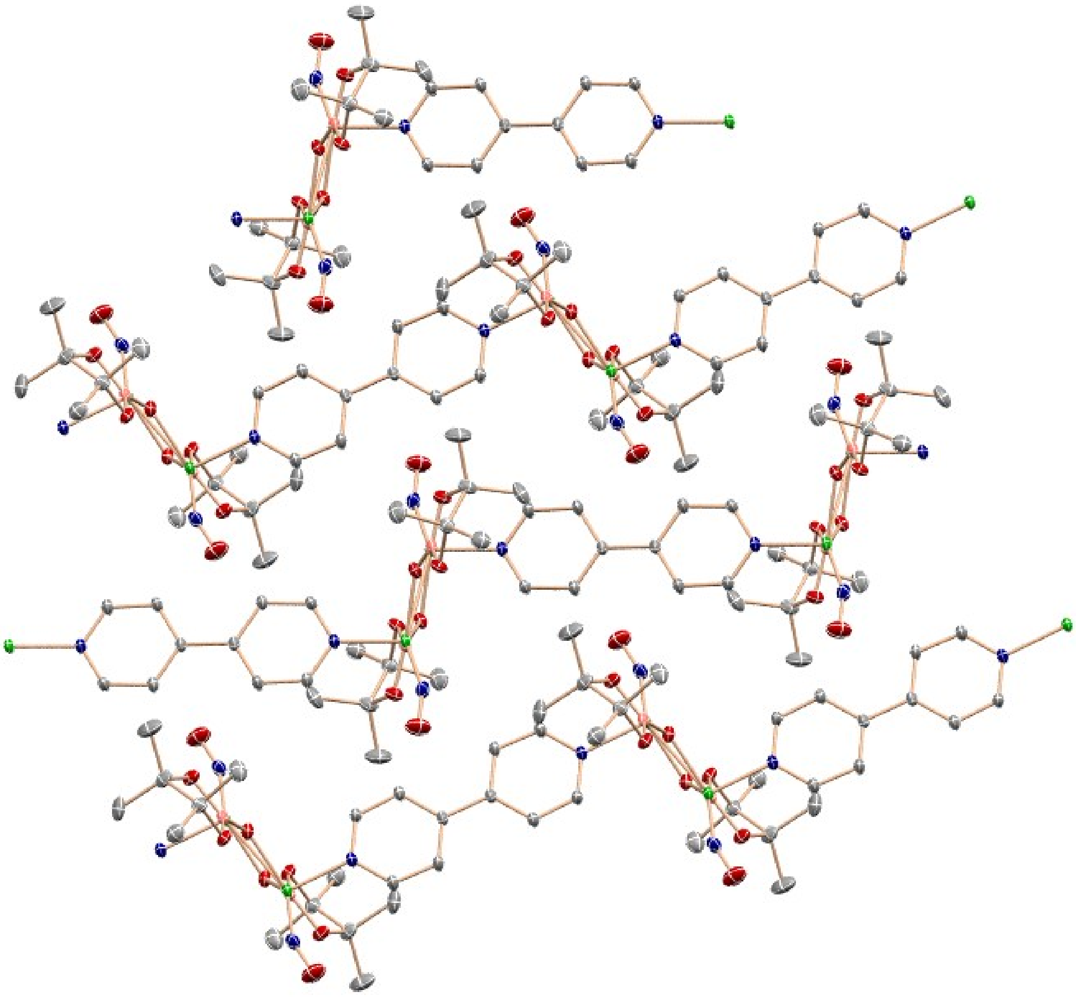

Fig S46. Crystal packing of $(OBPin)_2Ni_2(NO)_2(Bpy)$ viewed along the a axis

DFT

Computational Details

DFT[8] calculations were carried out using Gaussian 16[9]. Geometry optimizations were performed at the B3LYP-D3BJ/6-31G(d,p) level of theory[7] followed by single point energy calculations at the B3LYP-D3BJ/6-311G(d,p) level of theory. All optimized structures were confirmed to be minima by analyzing the harmonic frequencies.[11-13] Optimized structures have been attached as .mol files for viewing using the appropriate software (Mercury, Gaussian, ChemCraft, etc.)

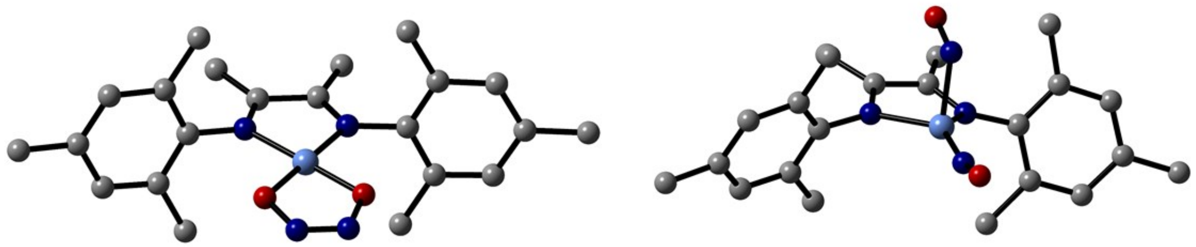

0 kcal/mol

+38 kcal/mol

Fig S47. Optimized geometries and relative energies for isomers of empirical formula (DIM)Ni(N_2O_2). The hyponitrite species is lower in energy; however, both the hyponitrite and the dinitrosyl are thermodynamically downhill with respect to (DIM)Ni(NO)(ONO) + $(Bpin)_2$Pz. Although the hyponitrite is lower in energy, our experimental results suggest that N-N bond formation does not happen between oxyanions at the same metal center.

(DIM)Ni$(NO)_2$ was geometry optimized as a singlet state both from two linear, and one bent and one linear starting structures and both optimized to the same structure, one bent and one nearly (∠NiNO 166°) linear. The bent NO occupies an apical position in a trigonal *pyramidal* structure with the linear NO nearly in the NiDIM plane. The linear NO has an N-Ni distance shorter by 0.2 Å than to the bent NO. In sum this electronic structure is adequately described as planar (DIM)Ni(NO) radical with bent NO apical, hence 18 valence electrons with one anionic and one cationic nitrosyl. The bent/linear nature of the calculated dinitrosyl complex may help facilitate N-N bond formation, with the linear nitrosyl having electrophilic character and the bent nitrosyl having nucleophilic character.

The (DIM)Ni$(NO)_2$ dinitrosyl triplet is lower in energy than the singlet state by 7.3 kcal/mol. Although the singlet has one bent and one linear nitrosyl, the triplet species is pseudo-two fold symmetric, with each Ni-N-O angle ~139°, which is in line with the expectation for neutral NO. From the corresponding orbital diagram as well as the spin density plot, there is considerable amount of unpaired spin located on the nitrosyl ligands. We hypothesize that the radical character on the nitrosyl ligands could play an important role in the N-N coupling step to form N_2O.

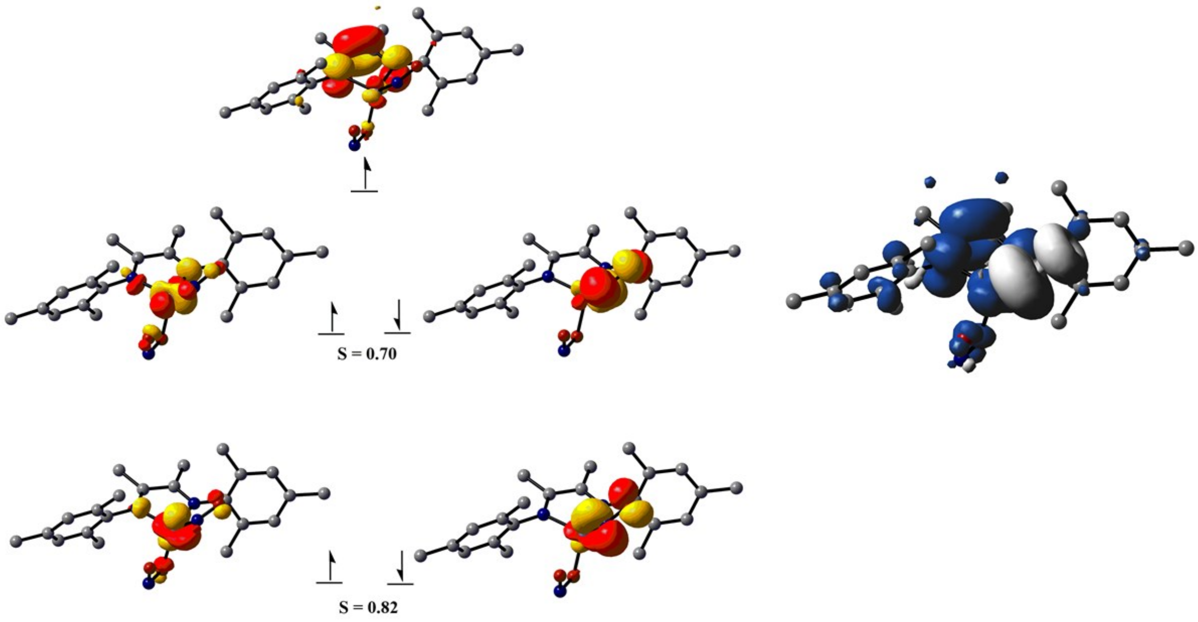

Figure S48. Corresponding orbital diagram (left) and spin density plot (right) for $[(DIM)Ni(NO)(ONO)]^{-}$. Corresponding orbitals plotted with isovalue of 0.05 au, and spin density is plotted with an isovalue of 0.002 au.

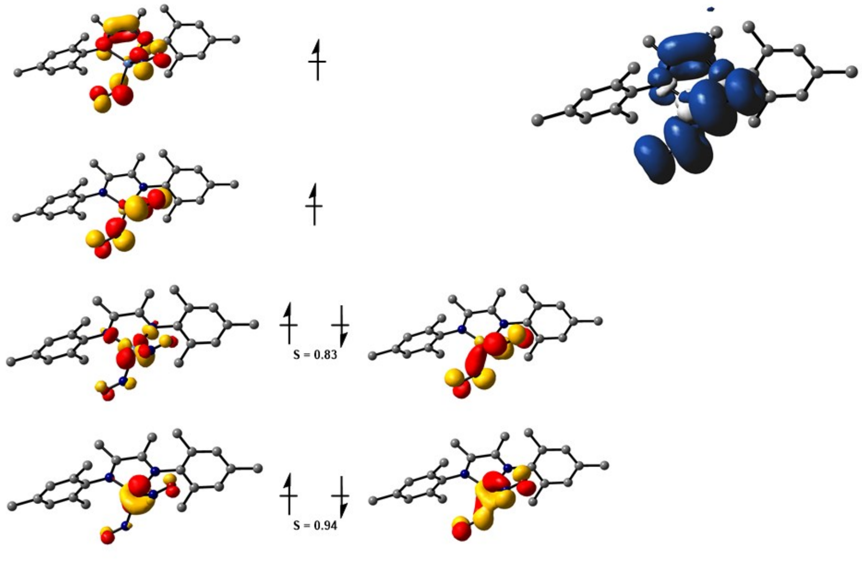

Figure S49. Corresponding orbital diagram (left) and spin density plot (right) for triplet $(DIM)Ni(NO)_2$. Corresponding orbitals plotted with isovalue of 0.05 au, and spin density is plotted with an isovalue of 0.002 au.

References

1. Ohmura, T.; Morimasa, Y.; Suginome, M. *J. Am. Chem. Soc.* 2015, **137**, 2852-2855.
2. Oshima, K.; Ohmura, T.; Suginome, M. *Chem. Commun*. 2012, **48**, 8571-8573.
3. Suryaraman, M. G.; Viswanathan, A. *J. Chem. Educ.* 1949, **26**, 594-596.
4. SAINT, Bruker Analytical X-Ray Systems, Madison, WI, current version.
5. SADABS, Bruker Analytical X-Ray Systems, Madison, WI, current version.
6. G. M. Sheldrick, Acta Cryst. A64, 112 - 122 (2008). Sheldrick, G.M. (2015). Acta Cryst. A71, 3-8.
7. O. V. Dolomanov, L. J. Bourhis, R. J. Gildea, J. A. K. Howard and H. Puschmann, J. Appl. Crystallogr., 2009, 42, 339–341.
8. Parr, R.G.; Yang, W. Density-functional theory of atoms and molecules; Oxford University Press: New York, 1989.
9. Gaussian 16, Revision B.01, M. J. Frisch, G. W. Trucks, H. B. Schlegel, G. E. Scuseria, M. A. Robb, J. R. Cheeseman, G. Scalmani, V. Barone, G. A. Petersson, H. Nakatsuji, X. Li, M. Caricato, A. V. Marenich, J. Bloino, B. G. Janesko, R. Gomperts, B. Mennucci, H. P. Hratchian, J. V. Ortiz, A. F. Izmaylov, J. L. Sonnenberg, D. Williams-Young, F. Ding, F. Lipparini, F. Egidi, J. Goings, B. Peng, A. Petrone, T. Henderson, D. Ranasinghe, V. G. Zakrzewski, J. Gao, N. Rega, G. Zheng, W. Liang, M. Hada, M. Ehara, K. Toyota, R. Fukuda, J. Hasegawa, M. Ishida, T. Nakajima, Y. Honda, O. Kitao, H. Nakai, T. Vreven, K. Throssell, J. A. Montgomery, Jr., J. E. Peralta, F. Ogliaro, M. J. Bearpark, J. J. Heyd, E. N. Brothers, K. N. Kudin, V. N. Staroverov, T. A. Keith, R. Kobayashi, J. Normand, K. Raghavachari, A. P. Rendell, J. C. Burant, S. S. Iyengar, J. Tomasi, M. Cossi, J. M. Millam, M. Klene, C. Adamo, R. Cammi, J. W. Ochterski, R. L. Martin, K. Morokuma, O. Farkas, J. B. Foresman, and D. J. Fox, Gaussian, Inc., Wallingford CT, 2016.
10. (a) Vosko, S. H.; Wilk, L.; Nusair, M. *Can. J. Phys*. 1980, **58**, 1200. (b) Lee, C.; Yang, W.; Parr, R.G. Phys. Rev. B 1988, **37**, 785. (c) Becke, A. D. *J. Chem. Phys*. 1993, **98**, 5648. (d) Stephens, P.J.; Devlin, F. J.; Chabalowski, C. F.; Frisch, M.J. *J. Phys. Chem*. 1994, **98**, 11623
11. Becke, A.D. *J. Chem. Phys*., 1993, **98**, 1372.
12. (a) Schlegel, H. B.; McDouall, J. J. in Computational Advances in Organic Chemistry; Oeretir, C; Csizmadia, I. G., Eds.; Kluwer Academic: Amsterdam, The Netherlands, 1991. (b) Bauernschmitt, R.; Ahlrichs, R. *J. Chem. Phys*. 1996, **104**, 9047.
13. Schlegel, H. B. *WIREs Comput. Mol. Sci*. 2011, **1**, 790.
14. Kraft, S. J.; Williams, U. J.; Daly, S. R.; Schelter, E. J.; Kozimor, S. A.; Boland, K. S.; Kikkawa, J. M.; Forrest, W. P.; Christensen, C. N.; Schwarz, D. E.; Fanwick, P. E.; Clark, D. L.; Conradson, S. D.; Bart, S. C. *Inorg. Chem*., 2011, **50**, 9838-9848.
